# The Diversity of Lipopeptides in the Pseudomonas syringae Complex Parallels Phylogeny and Sheds Light on Structural Diversification during Evolutionary History

**DOI:** 10.1128/spectrum.01456-22

**Published:** 2022-10-26

**Authors:** Alexandre Bricout, Cindy E. Morris, Charlotte Chandeysson, Matthieu Duban, Corinne Boistel, Gabrielle Chataigné, Didier Lecouturier, Philippe Jacques, Valérie Leclère, Alice Rochex

**Affiliations:** a Université de Lille, Université de Liège, UMRt BioEcoAgro 1158-INRAE, Métabolites Secondaires d’Origine Microbienne, Charles Viollette Institute, Lille, France; b Agence de la transition écologique (ADEME), Angers, France; c INRAE, Pathologie Végétale, Montfavet, France; d Université de Liège, Université de Lille, UMRt BioEcoAgro 1158-INRAE, Métabolites Secondaires d’Origine Microbienne, TERRA Teaching and Research Centre, Gembloux Agro-Bio Tech, Gembloux, Belgium; Shenzhen Bay Laboratory

**Keywords:** classification, factins, lipopeptide BGC, lipopeptides, mycins, peptins, *Pseudomonas syringae*, secondary metabolites

## Abstract

Pseudomonas spp. colonize diverse aquatic and terrestrial habitats and produce a wide variety of secondary metabolites, including lipopeptides. However, previous studies have often examined a limited number of lipopeptide-producing strains. In this study, we performed a systematic analysis of lipopeptide production across a wide data set of strains of the Pseudomonas syringae complex (724) by using a combined bioinformatics, mass spectrometry, and phylogenetics approach. The large P. syringae complex, which is composed of 13 phylogroups, is known to produce factins (including syringafactin-like lipopeptides), mycins (including syringomycin-like lipopeptides), and peptins (such as syringopeptins). We found that 80.8% of P. syringae strains produced lipopeptides and that factins were the most frequently produced (by 96% of the producing strains). P. syringae strains were either factin monoproducers or factin, mycin, and peptin coproducers or lipopeptide nonproducers in relation to their phylogenetic group. Our analyses led to the discovery of 42 new lipopeptides, bringing the number of lipopeptides identified in the P. syringae complex to 75. We also highlighted that factins have high structural resemblance and are widely distributed among the P. syringae complex, while mycins and peptins are highly structurally diverse and patchily distributed.

**IMPORTANCE** This study provides an insight into the P. syringae metabolome that emphasizes the high diversity of lipopeptides produced within the P. syringae complex. The production profiles of strains are closely related to their phylogenetic classification, indicating that structural diversification of lipopeptides parallels the phylogeny of this bacterial complex, thereby further illustrating the inherent importance of lipopeptides in the ecology of this group of bacteria throughout its evolutionary history. Furthermore, this overview of P. syringae lipopeptides led us to propose a refined classification that could be extended to the lipopeptides produced by other bacterial groups.

## INTRODUCTION

Lipopeptides are secondary metabolites whose production has been reported for several bacterial genera, including *Streptomyces* (1), *Burkholderia* (2), *Bacillus* (3), and Pseudomonas (4), and for some filamentous fungi (5, 6). These amphiphilic molecules, composed of a hydrophobic fatty acid tail linked to a hydrophilic peptide chain, manifest biosurfactant activities and impact several biological mechanisms, mainly bacterial motility, antimicrobial activity, pathogenicity to plants, and plant systemic resistance induction. Lipopeptides help microorganisms to survive and thrive by facilitating habitat colonization, nutrient uptake, and competition with other organisms. Lipopeptides are assembled by nonribosomal peptide synthetases (NRPSs), which are large multienzymatic complexes encoded by biosynthetic gene clusters (BGCs). NRPSs are structured into modules, one for each amino acid (AA) (or monomer) of the peptide chain. Each module is divided into domains that catalyze different reactions. The essential domains are the adenylation (A) domain, responsible for AA recognition and activation, the thiolation (T) domain, for thioester ligation to the NRPS during the assembly process, and the condensation (C) domain, for peptide bond formation. A thioesterase domain (Te domain) is usually present in the last module to ensure release and in some cases cyclization of the peptide neosynthesized. In the case of lipopeptide NRPSs, a starter C domain located at the beginning of the assembly chain attaches the first AA to the fatty acid, which can be of different lengths, degrees of hydroxylation, and saturation. More specifically, the synthesis of lipopeptides by Pseudomonas spp. involves a dual C/E domain that is in charge of both epimerization of an l-AA into the d-AA racemer and peptide bond catalysis. NRPSs generate a high diversity of lipopeptides that are gathered into families depending on the composition and length of the peptide chain. However, no systematic classification or nomenclature is established to classify and name lipopeptides whatever the producing organism, which sometimes introduces confusion.

Pseudomonas syringae bacteria are known to produce factins (which are linear lipo-octapeptides, such as syringafactins), mycins (which are cyclic lipononapeptides, such as syringomycins), and peptins (which are cyclic lipopeptides with a longer peptide chain, such as syringopeptins bearing 22 or 25 AA (7). Questions about the ecological and evolutionary processes that lead to the structural diversity of lipopeptides are raised and could be investigated by exploring the lipopeptide diversity within this group of organisms, which are closely related genetically yet diversified in their ecological tendencies. Members of the Pseudomonas syringae complex have emerged as excellent models in this regard for several reasons: (i) this is a large phylogenetic complex composed of 13 phylogroups (8, 9), (ii) this species complex inhabits the surface of plants and a wide range of terrestrial and aquatic habitats that could provide different selective forces for lipopeptide evolution (10, 11), (iii) hundreds of agricultural and environmental strains representing the full diversity of this group are readily available from a single source, maintained at INRAE in Montfavet, France, and (iv) lipopeptide diversity and prevalence within the P. syringae complex have probably not been fully elucidated, especially given that most studies have focused on a small number of agricultural and phylogroup 2 strains.

## RESULTS AND DISCUSSION

### Deciphering the P. syringae lipopeptide production frequency and structural diversity with a combined approach of mass spectrometry and bioinformatics.

**FIG 1:**
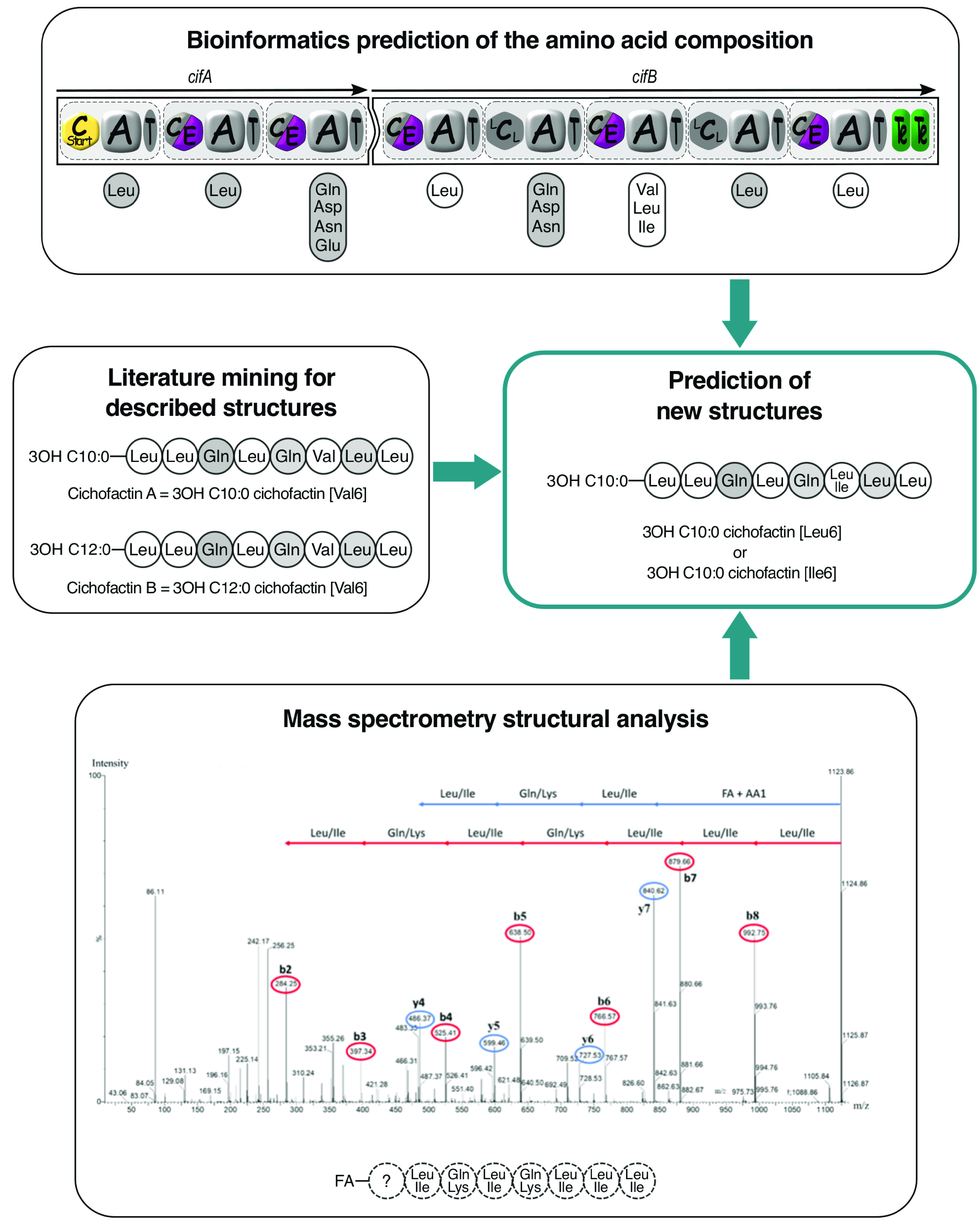
Combination of different approaches leading to new lipopeptide discovery. As an example, the workflow is applied to cichofactin produced by *P. cichorii* CFBP4407. Monomers with undefined isomery are represented by dotted circles. l-Monomers are in white; d-monomers are in gray.

To investigate lipopeptide production and diversity within the P. syringae complex, we studied a total of 724 strains (Table S1) collected from plants and different environmental habitats and representative of the phylogenetic diversity of this bacterial complex as they are distributed in the 13 phylogroups of the P. syringae complex. (See Fig. 7 for the phylogeny of the P. syringae complex reported by Berge et al. [8], reproduced in this paper.) We analyzed the strains with a combined approach using mass spectrometry (MS) and bioinformatics. The use of high-throughput matrix-assisted laser desorption ionization–time of flight (MALDI-TOF) mass spectrometry allowed us to rapidly analyze the 724 strains and to detect lipopeptide production *in vitro*. The whole genomes of 47 strains distributed in the 13 phylogroups of the P. syringae complex were sequenced (see Table S2 in the supplemental material). Bioinformatics tools allow detection of the presence of lipopeptide NRPS genes by searching for the signature sequences of NRPS domains from complete genomes. Lipopeptide BGCs were detected in 37 out of the 47 sequenced genomes and were then analyzed bioinformatically to predict the AA sequence and d/l-configuration through analysis of the A and C domains. However, some AA cannot be predicted (e.g., dhAbu) or predictions give only groups of several AA (e.g., Val, Leu, and Ile). The structures of the lipopeptides produced by the 37 strains were also analyzed by interpretation of fragment spectra obtained from mass spectrometry. However, in some cases fragmentations were partial, in particular for peptins, which have a stable peptidic cycle (12), or were not possible when lipopeptides were not produced in sufficient amounts. For each mass detected, we predicted the fatty acid and amino acid sequence of the corresponding lipopeptide from bioinformatics predictions and mass spectrometric data obtained for several producing strains (Fig. S1 to S5). Finally, the masses were assigned to known and new forms of factins, mycins, and peptins by comparison with data available from the literature. The strength of the combined use of bioinformatics and mass spectrometry lies in the complementarity of the data obtained, which is illustrated in Fig. 1. This approach was therefore particularly efficient and time saving insofar as it required neither purifying steps nor in-depth analyses to detect many lipopeptides and predict their structures. We found that 585 of the 724 strains of the collection have the capacity to produce lipopeptides, showing that these molecules have a prominent ecological role in the P. syringae complex. We detected a total of 67 lipopeptides, including 42 new lipopeptides (Table 1 and Data Set S1).

**TABLE 1:** Masses detected by MALDI-TOF MS and LC-MS^*a*^

[M + H]^+^	[M + Na]^+^	[M + K]^+^	Putative assignment			Calculated mass (Da)	No. of producing strains
			LP family	LP subfamily	LP		
			Factins				562
1,082.7	1,104.7	1,120.7		Syringafactin	Syringafactin A	1,081.74	429
1,096.8	1,118.8	1,134.8			Syringafactin B	1,095.75	369
1,096.8	1,118.8	1,134.8			Syringafactin C	1,095.75	369
1,110.8	1,132.8	1,148.8			Syringafactin D	1,109.77	182
1,124.8	1,146.8	1,162.8			Syringafactin E	1,123.78	117
1,124.8	1,146.8	1,162.8			Syringafactin F	1,123.78	117
1,109.8	1,131.8	1,147.8		Cichofactin	Cichofactin A	1,108.75	90
1,135.9	1,157.9	1,173.9			*3-OH C_12:1_ cichofactin [Val6]*	1,134.76	2^L^
1,137.8	1,159.8	1,175.8			Cichofactin B	1,136.75	93
1,163.9	1,185.9	1,201.9			*3-OH C_14:1_ cichofactin [Val6]*	1,162.79	2^L^
1,123.8	1,145.8	1,161.8			*3-OH C_10:0_ cichofactin [Leu/Ile6]*	1,122.76	97 + 1^L^
1,151.8	1,173.8	1,189.8			*3-OH C_12:0_ cichofactin [Leu/Ile6]*	1,150.79	97 + 1^L^
NO	1,201.8	1,217.8			*3-OH C_14:0_ cichofactin [Leu/Ile6]*	1,178.83	10
			Mycins				364
1,136.6	1,158.6	1,174.6		Syringotoxin	Syringotoxin B	1,135.55	58
1,152.6	1,174.6	1,190.6			*3,4-OH C_14:0_ syringotoxin*	1,151.55	87
1,164.6	NO	NO			*3-OH C_16:0_ syringotoxin*	1,163.58	17
1,180.6	1,202.6	1,218.6			*3,4-OH C_16:0_ syringotoxin*	1,179.58	57
1,163.6	NO	NO		Syringostatin	*C_14:0_ syringostatin*	1,162.60	20
1,179.6	1,201.6	1,217.6			Syringostatin A	1,178.59	36
1,195.6	1,217.6	1,233.6			Syringostatin B	1,194.59	36
1,207.6	NO	NO			*3-OH C_16:0_ syringostatin*	1,206.63	26
1,223.6	1,245.6	NO			*3,4-OH C_16:0_ syringostatin*	1,222.62	26
1,197.6	1,219.6	1,235.6		Syringomycin	Syringomycin A1	1,196.56	63
1,209.6	NO	NO			*C_12:0_ syringomycin*	1,208.60	195
1,225.6	1,247.6	1,263.6			Syringomycin E	1,224.59	234
1,237.6	NO	NO			*C_14:0_ syringomycin*	1,236.63	129
1,253.6	1,275.6	1,291.6			Syringomycin G	1,252.62	220
1,281.6	NO	NO			*3-OH C_16:0_ syringomycin*	1,280.65	43
1,207.6	NO	NO		Pseudomycin	Pseudomycin B	1,206.59	2
1,223.6	1,245.6	NO			Pseudomycin A	1,222.58	2
1,219.6	NO	NO			*C_16:0_ pseudomycin*	1,218.63	1
1,235.6	1,257.6	1,273.6			Pseudomycin C′	1,234.62	3
1,251.6	1,273.6	1,289.6			Pseudomycin C	1,250.62	4
1,263.7	NO	NO			*3-OH C_18:0_ pseudomycin*	1,262.65	1
1,279.7	NO	NO			*3,4-OH C_18:0_ pseudomycin*	1,278.65	1
1,249.6	NO	NO		Syringomycin-2	*C_16:1_ syringomycin-2*	1,248.63	2
1,265.6	1,287.6	1,303.6			*3-OH C_16:1_ syringomycin-2*	1,264.62	2
1,281.6	NO	NO			*3,4-OH C_16:1_ syringomycin-2*	1,280.62	2
1,293.7	NO	NO			*3-OH C_18:1_ syringomycin-2*	1,292.65	2
1,263,7	1,285.7	1,301.7		Pseudomycin-2	*3-OH C_16:0_ pseudomycin-2*	1,262.65	1
1,291.7	NO	NO			*3-OH C_18:0_ pseudomycin-2*	1,290.68	1
			Peptins				363
1,983.3	NO	NO		Cichopeptin-2	Not determined		7
1,997.3	2,019.3	2,035.3			Not determined		7
2,011.3	2,033.3	2,049.3			*3-OH C_10:0_ cichopeptin-2*	2,010.15	8
2,025.3	2,047.3	2,063.3			Not determined		8
2,063.3	2,085.3	2,101.3		Cichopeptin-3	Not determined		1
2,077.3	2,099.3	2,115.3			*3-OH C_12:1_ cichopeptin-3*	2,076.19	1
2,069.2	2,091.2	2,107.2		Cichorinotoxin	Cichorinotoxin	2,068.19	1
2,144.2	2,166.2	2,182.2		Syringopeptin 22	Syringopeptin 22A	2,143.20	109
2,172.2	2,194.2	2,210.2			Syringopeptin 22B	2,171.24	102
2,146.3	2,168.3	2,184.3		Syringopeptin 22-2	*3-OH C_10:0_ syringopeptin 22-2*	2,145.22	6
2,174.4	NO	NO			*3-OH C_12:0_ syringopeptin 22-2*	2,173.25	2
2,160.2	2,182.2	2,198.2		Syringopeptin 22-3	*3-OH C_10:0_ syringopeptin 22-3*	2,159.24	8
2,188.2	2,210.2	2,226.2			*3-OH C_12:0_ syringopeptin 22-3*	2,187.27	7
2,160.2	NO	2,198.2		Syringopeptin 508	*3-OH C_10:0_ syringopeptin 508*	2,159.24	2
2,188.2	2,210.2	2,226.2			Syringopeptin 508A	2,187.27	85
2,216.3	2,238.3	2,254.3			Syringopeptin 508B	2,215.30	81
2,399.4	2,421.4	2,437.4		Syringopeptin 25 or	Syringopeptin 25A or	2,398.36	46
				Syringopeptin 25-2	*3-OH C_10:0_ syringopeptin 25-2*	2,398.36	
2,427.4	2,449.4	2,465.4			Syringopeptin 25B or	2,426.39	17
					*3-OH C_12:0_ syringopeptin 25-2*	2,426.39	
2,413.4	2,435.4	2,451.4		Syringopeptin 25-3	*3-OH C_10:0_ syringopeptin 25-3*	2,412.38	10
2,441.4	2,463.4	2,479.4		Syringopeptin 25-4	*3-OH C_10:0_ syringopeptin 25-4*	2,440.41	8
2,469.4	NO	2,507.4			*3-OH C_12:0_ syringopeptin 25-4*	2,468.44	3
2,443.5	2,465.5	2,481.5		Syringopeptin 25-5	*3-OH C_10:0_ syringopeptin 25-5*	2,442.43	30
2,445.3	2,467.3	2,483.3		Syringopeptin 25-6	*3-OH C_10:0_ syringopeptin 25-6*	2,444.40	51
2,473.3	2,495.3	2,511.3			*3-OH C_12:0_ syringopeptin 25-6*	2,472.44	39

a Assignments of all lipopeptide (LP) mass peaks to known and new subfamilies of factins, mycins, and peptins were based on MALDI-TOF mass spectrometry analysis of the 724 strains (Data Set S1). Assignments of mass peaks to individual LPs were supported from bioinformatics and fragmentation data (Fig. S1 to S5) obtained for 37 LP-producing strains listed in Table S2. New molecules observed in this study are in italics. NO, not observed. A superscript “L” indicates observation in liquid culture supernatants only.

### Widespread factin production in the P. syringae complex.

**FIG 2:**
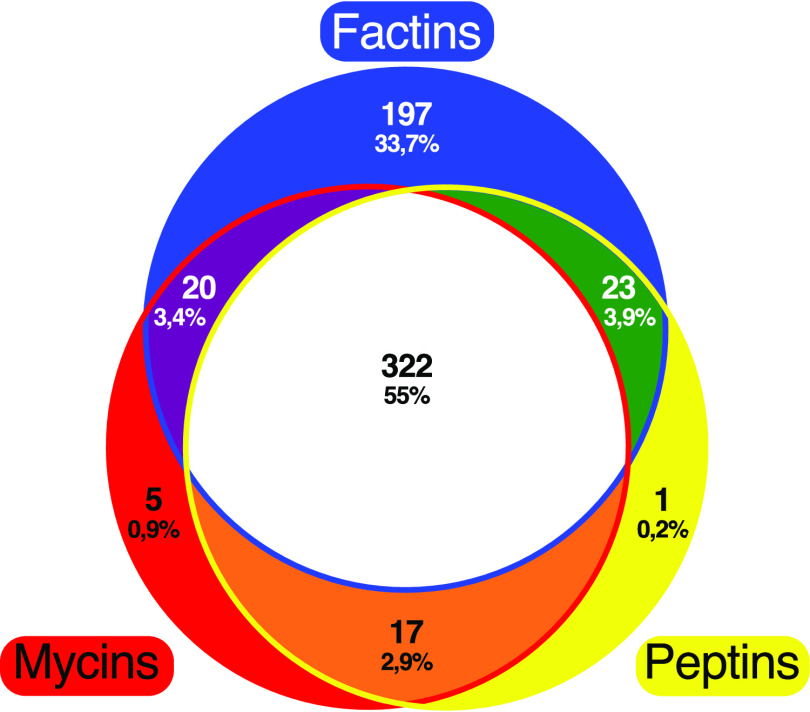
Distribution of lipopeptide families over the 585 producing strains from the P. syringae collection. The blue circle represents factin-producing strains, the red circle represents mycin-producing strains, and the yellow circle represents peptin-producing strains. The number of strains and the percentage over the 585 producing strains in the collection are mentioned in each corresponding area.

Factins were found to be coproduced with both mycins and peptins for more than half of the lipopeptide-producing strains (55%) and were also detected to be produced alone for many producing strains (33.7%). In rarer cases, factins were detected with only mycins (3.4% of the strains) or only peptins (3.9% of the strains) (Fig. 2). Thus, factins appeared to be the most frequently occurring lipopeptides; they are produced by 562 strains, which represents 96% of the lipopeptide-producing strains. In order to better understand the absence of lipopeptide production, we compared BGC detection to lipopeptide production (Table S2). There were multiple instances where BGCs were detected but not the compounds, probably because of poor production or ionization of lipopeptides leading to a signal below the detection threshold of the mass spectrometer and therefore to an underestimation of the number of producing strains. The absence of detection of some lipopeptides could also be due to the absence of the corresponding BGCs. *In silico* analyses of sequenced genomes have demonstrated that at least 10 strains do not harbor any BGC for synthesis of lipopeptides, highlighting that some P. syringae strains are lipopeptide nonproducers (Table S2). Moreover, the genomes of at least 15 strains of the collection harbor a BGC only for factin production, confirming that some P. syringae strains can be factin monoproducers, such as P. syringae pv. tomato strain DC3000, from which factins were identified for the first time (7, 13). Our genomic analysis showed that the sequenced genomes of P. syringae strains harbor no BCGs, one factin BGC, or three lipopeptide BGCs.

**FIG 3:**
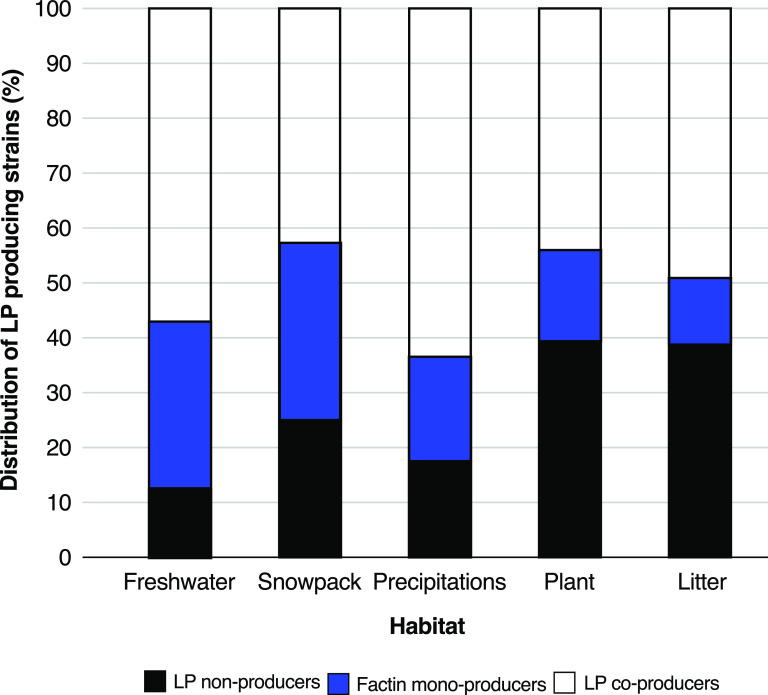
Relationship between the source of isolation and the lipopeptide (LP) production profile of P. syringae strains. The percent distribution of LP-producing strains among the 724 strains of the P. syringae collection is shown.

Factin production has often been ignored or disregarded. Syringafactins have been described only for two strains of P. syringae (13, 14), cichofactins for only about 15 strains of *P. cichorii* (15, 16), and recently virginiafactins for one Pseudomonas sp. strain outside the P. syringae complex (16). Our results therefore demonstrate that production of factins, alone or in combination with mycins and peptins, is actually a widespread trait of P. syringae bacteria (562 factin-producing strains out of 724 strains studied) whatever their habitat. About 85% of the strains isolated from freshwater or precipitation, 75% from snowpack and 60% from plant and litter, were factin monoproducers or lipopeptide coproducers (Fig. 3). It seems that the proportion of nonproducers is higher among strains isolated from plants or litter (about 40%) but lipopeptide-producing strains from plant or litter are mainly producers of the three families, factins, mycins, and peptins. However, they are proportionally less represented in our collection. An in-depth study with a larger number of plant-associated strains should be done to examine the relationship between the source of isolation and the lipopeptide production profile. Factin synthesis has probably been highly maintained in P. syringae during evolution because of the physicochemical properties of factins, which are important for colonization of their environment. Berti et al. (13) and Pauwelyn et al. (15) have shown that factins have biosurfactant activities and enhance bacterial motility through swarming, as factin-deficient mutants lost these activities (13, 15). Swarming also favors plant pathogenicity (17). The strong biosurfactant and hygroscopic activities of factins are also major assets that can promote access to water and nutrients, especially at the bacterium-plant interface (14, 18). For example, they could promote the solubilization of pectin degradation products generated by pectolytic enzymes produced by strains of phylogroup 07 (19). Finally, cichofactins also have antimicrobial properties that certainly favor the development of P. syringae strains at the expense of other competing microorganisms (15). Interestingly, the surfactin-like lipopeptides that are frequently produced by strains of the Bacillus subtilis group (20, 21) present the same properties as factins (e.g., biosurfactant activity and influence on swarming).

### Low diversity of factins versus high diversity of mycins and peptins.

**FIG 4:**
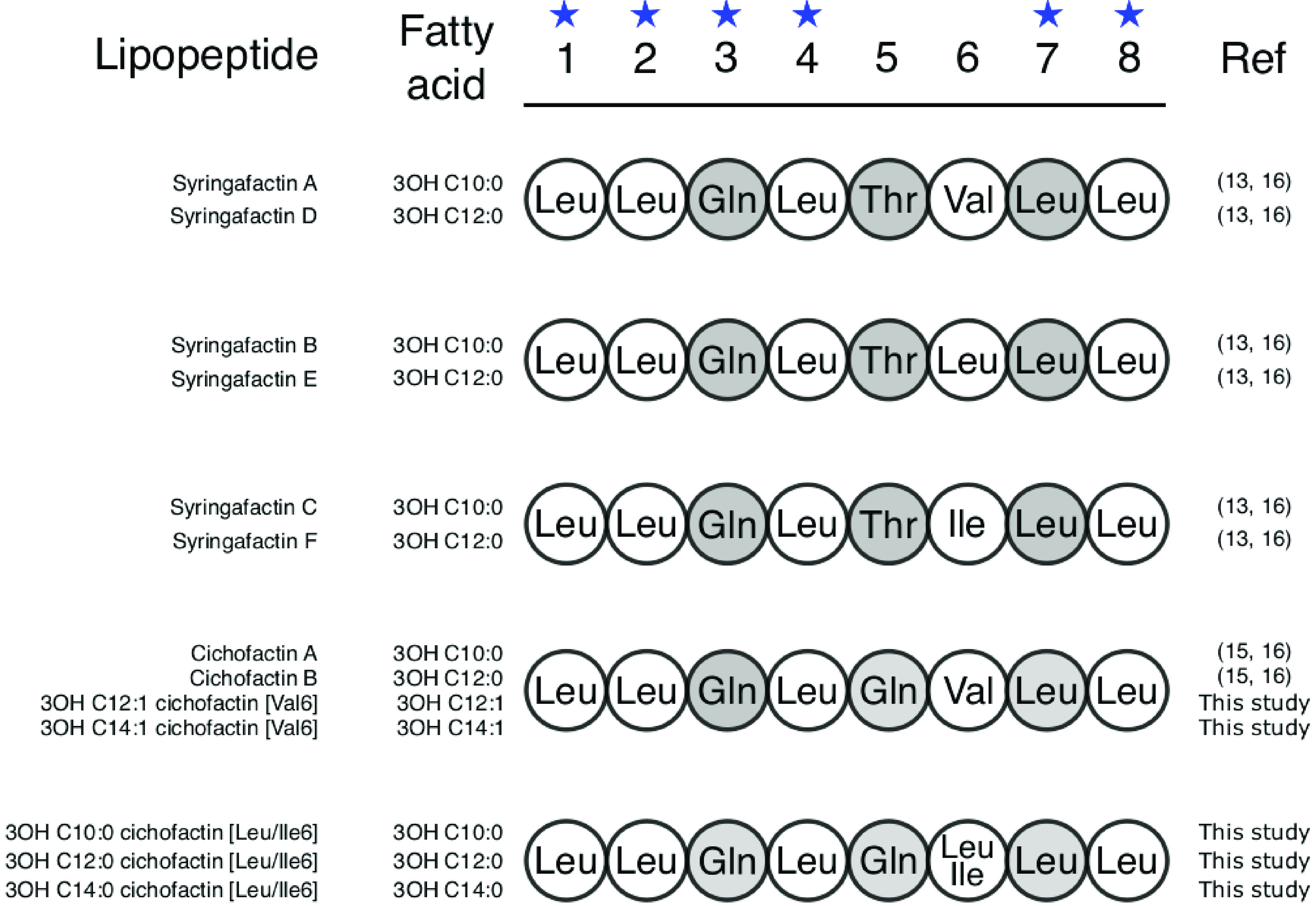
Predicted structures of new factins compared to the previously described factins produced by the P. syringae complex. l-Monomers are in white, d-monomers are in gray. The star above the monomer position number indicates conserved monomers.

Within the factin family, masses corresponding to syringafactin forms A to F (13) were detected together in a large number of the studied strains. In other strains, masses assigned to cichofactins A and B (15) and to 5 new forms of cichofactin were observed to be coproduced (Table 1 and Data Set S1). Cichofactins differ from syringafactins at position 5 of the peptide chain (Fig. 4) due to a difference between the 5th A domains (15). The new cichofactins differ from cichofactins A and B, either on the fatty acid tail or at position 6 of the peptide chain due to the flexibility of the 6th A domain (Fig. 4). It is known that certain A domains are able to recruit structurally related AAs such as valine, leucine, and isoleucine (22–24). The BGCs for factin production analyzed in this study by bioinformatics have the same organization as those previously reported for syringafactins (13) and cichofactins (15). They contained two NRPS genes (*syfA* and *syfB* or *cifA* and *cifB*) encoding two enzymes of 3 and 5 modules (Fig. S1 and S2).

**FIG 5:**
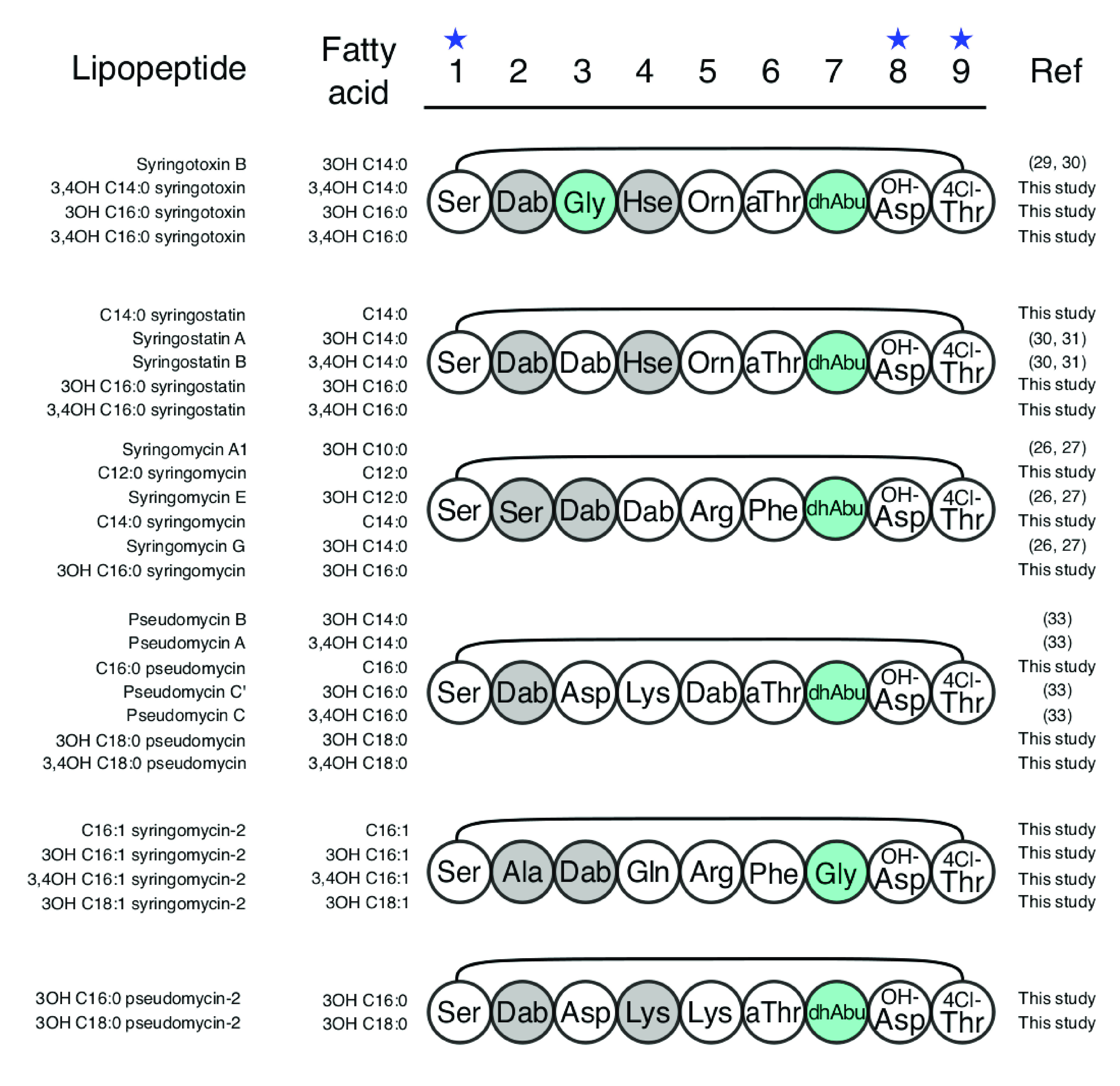
Predicted structures of new mycins compared to the previously described mycins produced by the P. syringae complex. l-Monomers are in white, d-monomers are in gray, and achiral monomers are in blue. The star above the monomer position number indicates conserved monomers.

In the mycin family, the *m/z* corresponding to syringomycins E and G (25–27), which are the most commonly described in the literature, were also the most frequently detected in this study (Table 1 and Data Set S1). In some strains, they were coproduced with syringomycin A1 and three new syringomycins. All other mycins known to be produced by strains of P. syringae, namely, syringotoxin B (28–30), syringostatins A and B (30, 31), and pseudomycins A, B, C, and C′ (32, 33), were also detected. The different forms of a same mycin (e.g., syringostatins A and B) were often coproduced with new forms by a single strain (Table 1 and Data Set S1). The mass differences of 16 Da and 28 Da observed were attributed to variations in size or degree of hydroxylation of the fatty acid (Fig. 5). Further analysis led to the detection of new mycins we called syringomycin-2 and pseudomycin-2, which bring the total of new mycin forms to 18 (Table 1). Syringomycins-2 differ from syringomycin at positions 2, 4, and 7 in the peptide chain and pseudomycins-2 differ from pseudomycins at position 5 (Fig. 4). Bioinformatics analysis of the BGCs for the production of syringomycin and syringomycin-2 detected in this study showed they were organized as previously reported for syringomycins (4). They contained two NRPS genes (*syrE* and *syrB1*) flanked by three genes (*syrP*, *syrB2*, and *syrC*) encoding external enzymes involved in mycin synthesis. The gene *syrE* codes for eight full C-A-T modules and a ninth split module lacking an A domain, and the gene *syrB1* codes for a split A-T module (Fig. S3C and E). The BGCs encoding the NRPSs of other mycins had not been described until now; they contained three NRPS genes (*syrE1*, *syrE2*, and *syrB1*), like those for thanamycin and nunamycin (4, 7), which are mycins produced by Pseudomonas spp. outside the P. syringae complex. The BGCs encoding syringotoxins, syrinstatins, and pseudomycins are organized like that of thanamycin. The gene *syrE1* codes for five full modules, and the gene *syrE2* codes for 4 modules, with the last module lacking an A domain (Fig. S3A, B and D). The BGC encoding pseudomycin-2 is organized like that of nunamycin (34). The genes *syrE1* and *syrE2* code for 5 and 4 modules, which both have the last module lacking an A domain (Fig. S3F). The peptide chains of mycins appear to be more diverse than previously known; they differ in the amino acid sequence between AA 2 and AA 7.

**FIG 6:**
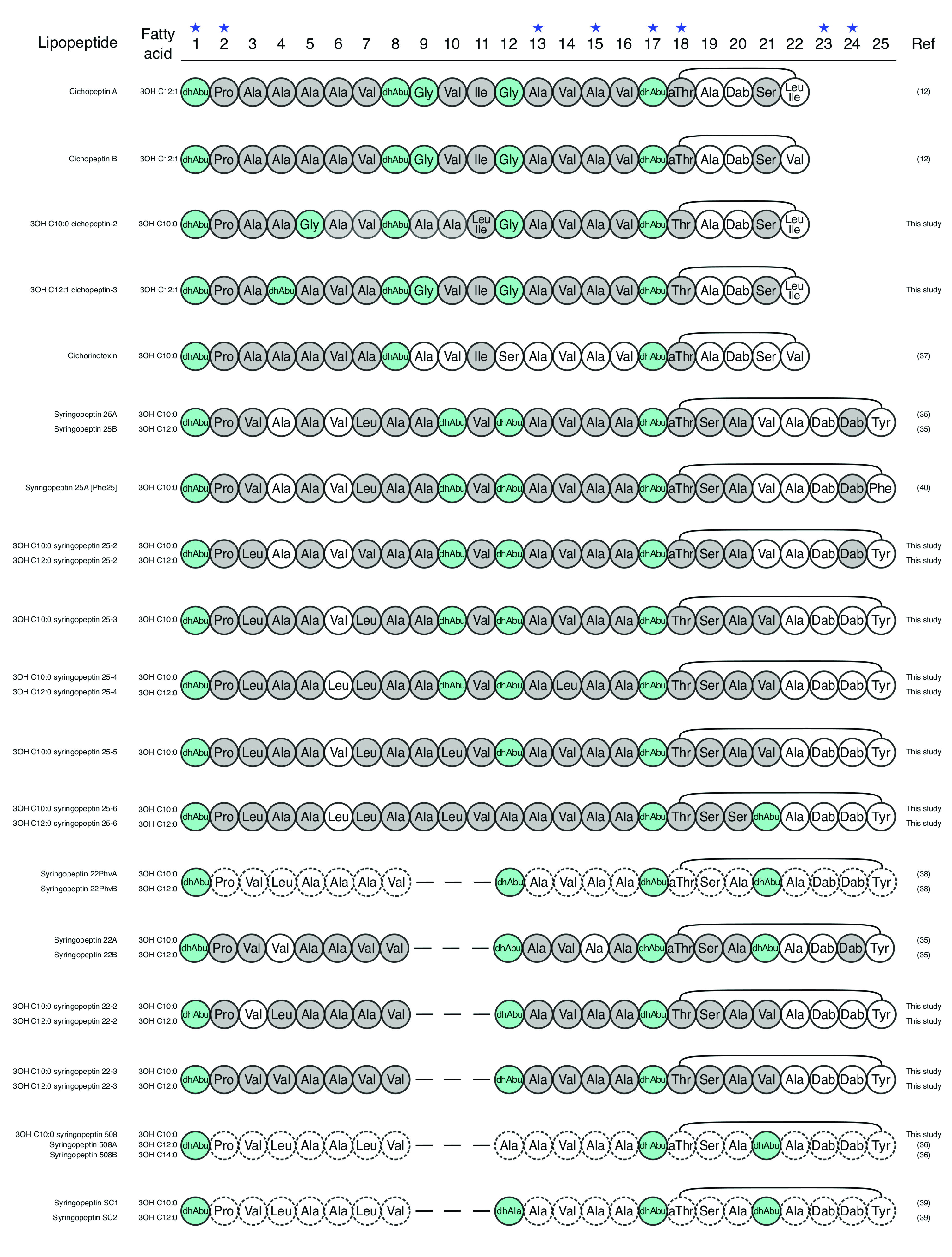
Predicted structures of new peptins compared to the previously described peptins produced by the P. syringae complex. l-Monomers are in white, d-monomers are in gray, and achiral monomers are in blue. The star above the monomer position number indicates conserved monomers.

Regarding the peptin family, syringopeptins 22A and 22B (35) and syringopeptins 508A and 508B (36) were the most frequently observed. Cichorinotoxin (37) and syringopeptins (25A and 25B) (35) as well as 19 new peptins were also detected (Table 1 and Data Set S1). The different forms of a same peptin were often coproduced by the same strain as observed for mycins. The new syringopeptins 22-2 and 22-3 are structurally close to syringopeptins 22Phv and 22, respectively (38). Their only difference with the latter is located at position 18 of the peptide chain (Fig. 6). The new cichopeptins have structures similar to that of cichopeptin A (12), with several AA variations between positions 4 and 10 in the peptide chain (Fig. 6). We could predict complete structures only for new cichopeptins, 3-OH C_10:0_ cichopeptin-2 and 3-OH C_12:1_ cichopeptin-3, as other forms were not produced in amounts sufficient to be characterized. Concerning the new 25 AA peptins, our analyses showed that they have structural resemblance to syringopeptin 25 (35) (Fig. 6). Syringopeptin 25-2 has the same *m/z* as the known syringopeptin 25 and presents AA differences at position 3 and 7. Syringopeptins 25-3, 25-4, and 25-5 differ by the replacement of certain AA by leucine at various positions of the peptide chain: position 3 for syringopeptin 25-3, positions 3 and 10 for syringopeptin 25-4, and positions 3, 6, and 14 for syringopeptin 25-5 (Fig. 6). In contrast, the differences observed between syringopeptin 25-6 and syringopeptin 25 are much greater. They actually differ both in the linear part, at positions 3, 6, 10, and 12 of the peptide chain, and in the cyclic part, at positions 20 and 21 (Fig. 6). Eight other peptins known to be produced by strains of the P. syringae complex, i.e., cichopeptins A and B (12), syringopeptin SC1 and SC2 (1, 2, 39), syringopeptins 22PhvA and -B (38), [Phe^25^]syringopeptin 25 (40), and peptin 31 (41), were not detected. Bioinformatics analyses showed that the organization of the peptin BGCs detected in the studied strains is identical to those previously described (4, 7). They contained three genes (*cipA*, *cipB*, and *cipC* or *sypA*, *sypB*, and *sypC*) encoding enzymes with 9-4-9 modules (cichopeptins and cichorinotoxin) (Fig. S4A and B), 5-5-12 modules (syringopeptins 22) (Fig. S4E and F), and 8-5-12 modules (syringopeptins 25) (Fig. S5B to F). This study reveals that the peptide chains of peptins are highly variable; they differ in particular in the linear part between positions 3 and 12 and in the cyclic part.

Finally, our study allowed detection of 42 new lipopeptides and prediction of structures for 5 factins (Fig. S6A), 18 mycins (Fig. S6B), and 15 peptins out of 19 (Fig. S6C). This brings the number of known lipopeptides in P. syringae to 75 (13 factins, 28 mycins, and 34 peptins) and reveals a notably high structural diversity of lipopeptides for a single bacterial complex; the number of described lipopeptides is now about 160, considering all other Pseudomonas species. The lipopeptide diversity in P. syringae strains could be dependent on the growth medium composition, because of the substrate flexibility of the A domains of some NRPSs (22, 23), and therefore may be higher than that observed. We cultivated the strains on two different solid media (potato dextrose agar [PDA] and syringomycin recovery medium [SRM]) to increase the probability of detecting a high number of lipopeptide forms, but we observed flexibility only on the 6th A domain of factin NRPS. Interestingly, mycins and peptins known to be antimicrobial and phytotoxic show much more structural diversity than factins not only in the P. syringae complex but also in the Pseudomonas fluorescens complex and in the Pseudomonas asplenii group, which contain producers of other mycins, such as cormycins, nunamycins, and thanamycins, and other peptins, such as fuscopeptins, nunapeptin, and sclerosin (7). Variations in the composition and length of both the fatty acid and the peptide chain that were observed between lipopeptides of a given family are likely to influence their level of activity against a given target, as demonstrated for known mycins and peptins (33, 36, 38, 42) and lipopeptides of the viscosin family (43). However, it is arduous to determine to what extent these structural differences are able to modify their interaction with a targeted organism since the antimicrobial and phytotoxic activities of lipopeptides remain poorly documented (44, 45).

### Reclassification of lipopeptides.

The discovery of a high number of new molecules led us to refine the classification of P. syringae lipopeptides and to propose to extend this classification to all lipopeptides. Lipopeptides are usually classified into families (or groups) composed of lipopeptides having close chemical structures in terms of length and composition of the peptide moiety and number of AAs composing the lactone ring. We proposed a supplementary hierarchical classification level that is subfamily. Subfamilies group similar family members that can be produced by a same strain. Strains have the capacity to coproduce different lipopeptides of the same family, as NRPSs can synthesize lipopeptides with different fatty acid tail or peptide moieties due to their inherent flexibility. Hence, lipopeptides that are never coproduced in the same strain are considered to be synthesized from different NRPSs and are classified in different subfamilies. In parallel to this classification, we also propose a new nomenclature which is meant to be descriptive of the lipopeptide structures and that will allow any newly identified lipopeptide to be described and named (Table S3). The name of a new subfamily should be the same as the closest known subfamily in terms of lipopeptide structure and/or NRPS organization, incremented by 1 (e.g., syringopeptins 25, 25-2, 25-3, 25-4, 25-5, and 25-6). Within each subfamily, the names of the different lipopeptides could mention the fatty acid length, degrees of hydroxylation and saturation, and AA variations (e.g., 3-OH C_12:1_ cichofactin [Val6]). For lipopeptides that were already described, we also defined a new name with the new nomenclature as a synonym of the original name.

Among the 42 new lipopeptides detected during this work, 18 were attributed to new members of known subfamilies and 24 were attributed to two new mycin subfamilies (syringomycin-2 and pseudomycin-2) and 9 new peptin subfamilies (syringopeptins 22-2 and 22-3, cichopeptin-2 and -3, and syringopeptins 25-2, 25-3, 25-4, 25-5, and 25-6).

Finally, this classification and nomenclature could thus be used to classify and name any new lipopeptide discovered in future studies.

### Lipopeptide production is correlated with phylogenetic classification of P. syringae strains.

**FIG 7:**
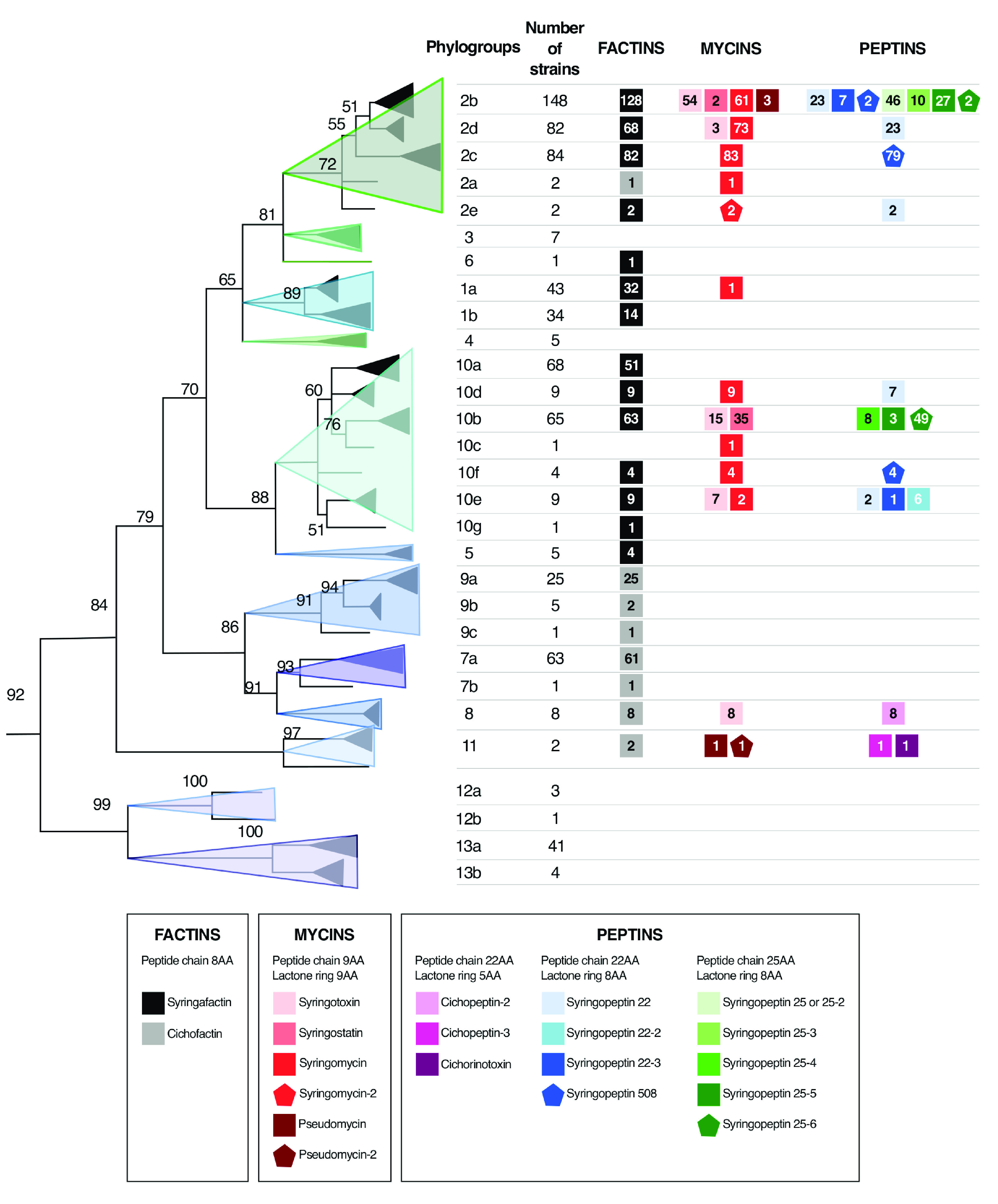
Phylogenetic distribution of lipopeptide subfamilies produced by 724 strains of the P. syringae complex. The phylogenetic tree was constructed by Berge et al. (8) and was reproduced according to the terms of the Creative Commons Attribution License. The tree was constructed on the concatenated sequences of four housekeeping genes of 216 P. syringae strains: *cts* (encoding citrate synthase), *gapA* (glyceraldehyde-3-phosphate dehydrogenase A), *rpoD* (RNA polymerase sigma70 factor), and *gyrB* (gyrase B). Lipopeptide subfamilies are represented with colored squares or pentagons. The number of producing strains is indicated inside each symbol.

Production profiles of strains were related to their phylogenetic classification, which was previously established by Berge et al. (8). Lipopeptide-producing strains belong to phylogroups 1, 2, 5, 6, 7, 8, 9, 10, and 11, whereas none of the strains of the phylogroups 3, 4, 12, and 13 were found to produce lipopeptides (Fig. 7) or to harbor lipopeptide NRPS BGCs in their genome (Table S2). The comparison of the lipopeptide production profile and the phylogenetic tree highlights three important points: (i) syringafactin producers and cichofactin producers form two phylogenetically separated sets sharing a factin-producing common ancestor (with the exception of clade 2a, which probably contains a misclassified strain), (ii) factin monoproducers and three family coproducers belong to distinct phylogroups and clades, and (iii) syringopeptin and cichopeptin producers belong to different phylogroups and are coproduced with syringafactins and cichofactins, respectively. Thus, syringafactin-monoproducing strains belong to phylogroups 1 (a and b) and 5, 6, and 10 (a and g), while cichofactin-monoproducing strains belong to phylogroups 7 (a and b) and 9 (a, b, and c). Syringafactins are produced in combination with mycins and syringopeptins 22 in phylogroups 2 (b, c, d, and e) and 10 (d, e, and f) and with syringopeptins 25 in phylogroups 2b and 10b. Cichofactins are produced in combination with mycins and cichopeptins in phylogroups 8 and 11. Mycins seemed to be less phylogroup dependent however syringomycins and syringostatins were found only in phylogroups 2 and 10.

Factins are highly conserved, as they are found in many phylogroups (phylogroups 1, 2, 5, 6, 7, 8, 9, 10, and 11) and have high structural resemblance. The peptide chains of syringafactins and cichofactins differ in their sequences at position 5, with AA that have similar biochemical properties: threonine (syringafactins) and glutamine (cichofactins), both bearing polar uncharged side chains (Fig. 4). The maintenance of these amino acid sequences through evolution indicates that they are essential for biosurfactant activity of factins. The broad phylogenetic distribution of factins suggests that a factin BGC was present in a common ancestor of the P. syringae species complex. Interestingly, the separation of syringafactins and cichofactins in the phylogenetic tree (Fig. 7) corresponds to the separation of the P. syringae species complex into primary phylogroups (phylogroups 1, 2, 3, 4, 5, 6, and 10) and secondary phylogroups (phylogroups 7, 8, 9, 11, 12, and 13) which are considered quite genetically distinct (46). Thus, syringafactins are widely distributed in the primary phylogroups and cichofactins in the secondary phylogroups. According to studies summarized by Morris and Moury, phylogroup 7 (cichofactin monoproducer) diverged after phylogroup 1 (syringafactin monoproducer) (47), which likely indicates that cichofactin producers diverged after syringafactin producers.

In contrast, mycins and peptins are highly diversified and are found in only four distant phylogroups (phylogroups 2, 8, 10, and 11). It is worth noting that two clades (10a and 10g) in phylogroup 10 do not produce mycins and peptins. The phylogenetic separation of peptins (syringopeptins in the primary group and cichopeptins in the secondary group) suggests that they were acquired by horizontal gene transfer after the divergence of the primary and secondary groups. The patchy distribution of mycins and peptins suggests that several gain/loss events occurred after divergence. The presence of mycin and peptin BGCs in strains belonging to the P. fluorescens lineage supports this hypothesis (7). However, gene acquisition by a common ancestor followed by the loss of mycin and peptin NRPS genes in some phylogroups cannot be discarded. Our results show that the peptide sequences of mycins and peptins are subject to strong diversification both intra- and interphylogroups. To date, evolutionary forces that have shaped the diversity of lipopeptides are largely unknown. Some AAs of mycins (positions 1, 8, and 9) and peptins (positions 1 and 2, 14 and 15, or 17 and 18) are probably highly conserved by negative selection because of their crucial role in the biological activity of these molecules. The variations observed for the other AAs could be due to a neutral evolutionary process or a positive selection that would increase their potency in microbe-microbe or host-microbe interactions.

The differences of lipopeptide production profiles raise also the question of their impact on the pathogenic activity of P. syringae on plants. Mycins and peptins have phytotoxic activity that enhance the phytopathogenic activity of P. syringae strains (48), even if the disease still occurs in their absence. Regarding factins, their phytotoxic activity has not been well studied. Cichofactins seem to be involved in pathogenicity but do not display a direct toxicity to plants (15), while no data are available for syringafactins. However, lipopeptide production profile is not directly correlated with pathogenicity. Many strains of phylogroups 1, 5, 6, and 7, which are factin monoproducers, as well as strains in phylogroups 2 and 10, which coproduce factins, mycins, and peptins, are plant pathogenic (8), but a high proportion of strains in phylogroups 2 and 10, in particular in clades 2c and 10b, have not been shown to be pathogenic in laboratory tests on a cantaloupe indicator plant (8). Concerning strains in clade 2c, they have defective type III secretion systems, suggesting that lipopeptide action in pathogenicity may be mediated by this secretion system (49). Some strains would produce small amount of syringomycin solely for the purpose of accessing plant metabolites without altering their tissues (50). The production of mycins and peptins could increase the extent of host range of the producing strains. Strains belonging to phylogroup 2 (triproducing strains) actually have a wider host range than strains of phylogroup 1 (syringafactin-monoproducing strains) (17). Interestingly, factin-monoproducing strains have a greater number of effector genes for strong virulence factors than do strains producing the three lipopeptide families. Monoproducing strains have certainly adapted their pathogenic strategy by favoring the synthesis of effector proteins to compensate for the absence of mycins and peptins (8, 51, 52).

Finally, the parallel observed between production profiles of strains at the family and subfamily levels and their phylogenetic classification showed the importance of lipopeptides in the ecology of this group of bacteria. It would be interesting to explore these aspects for other groups of lipopeptide-producing bacteria.

## MATERIALS AND METHODS

### Pseudomonas strains.

The 724 strains used in this study are listed in Table S1. They were taken from the collection of INRAE, Montfavet, France, and were previously characterized (8). P. syringae B301D, a producer of factins, mycins, and peptins, was used as a reference strain. This strain was obtained from the DSMZ (German collection of microorganisms; DSM no. 1241). All strains were stored in 40% (vol/vol) glycerol solutions at either −20°C for short-term storage or −80°C for long-term storage.

### Growth conditions for lipopeptide production and cell-free supernatant preparation.

Cultures on solid media were performed on either potato dextrose agar (PDA) or syringomycin recovery medium (SRM) agar plates. SRM is optimized for syringomycin production (53). Bacterial strains were grown on both media for 72 h at 25°C. Cultures in liquid media were performed in SRM under at 25°C agitation (160 rpm). A 48-h colony isolated on tryptic soy agar (TSA) was used to inoculate a 10-mL preculture. After 48 h of incubation, the preculture was used to inoculate a 100-mL culture. After 72 h of incubation, the cultures were centrifuged at 12,860 × *g* for 15 min. The supernatants were then filter sterilized through 0.2-μm polyethersulfone membranes and kept at −20°C until use. Cell-free supernatant of strain *P. cichorii* CFBP4407 was submitted to ultrafiltration to semipurify its lipopeptides. Thus, it was filtrated across a 10-kDa-cutoff membrane in a Vivaspin 20 ultrafiltration tube (Sartorius, Slonehouse, UK), which was centrifuged for 30 min at 4,000 × *g*. The material retained on the filter was then subjected to a diafiltration step to remove small molecules and residual salts.

### Detection and structural analysis of lipopeptides.

Detection of lipopeptides was realized for the 724 strains using matrix-assisted laser desorption ionization–time of flight mass spectrometry (MALDI-TOF MS). Structural analyses of mycins and peptins were performed by MS-MS fragmentation of [M+H]^+^ by MALDI-TOF MS. Structural analyses of factins were performed by MS-MS fragmentation of [M+H]^+^ by high-pressure liquid chromatography (HPLC)-MS because no [M+H]^+^ ions were generated by MALDI-TOF MS.

### (i) MALDI-TOF-MS.

The analyses were performed from whole cells grown on either PDA or SRM solid media by using an Autoflex Speed MALDI-TOF mass spectrometer (Bruker Daltonik, Bremen, Germany) controlled by FlexControl 3.4 software. The masses of the lipopeptides detected for the reference strain P. syringae B301D were used as standards to support assignments. Individual colonies were smeared on a ground steel MALDI target plate from Bruker Daltonik, washed twice with a 0.1% (vol/vol) trifluoroacetic acid (TFA) solution, and covered by a 70% (vol/vol) formic acid solution. Once dried, samples were overlaid with 1 μL of α-cyano-4-hydroxycinnamic acid matrix (10 mg/mL dissolved in H_2_O-acetonitrile-TFA, 50:47.5:2.5 [vol/vol/vol]). Measurements were performed in reflector positive mode between 800 Da and 5,000 Da, each spectrum being the result of a 2,000-laser shot accumulation whose intensity varied from 50% to 70%. Parameters were set as follows: ion source voltage 1, 19 kV; voltage 2, 16.6 kV; lens tension, 8 kV; and pulsed extraction, 120 ns. Ions of interest were fragmented in LIFT mode without collision gas, device parameters being set as follows: ion source voltage 1, 6 kV; voltage 2, 5.3 kV; and lens tension, 3 kV. MS and MS-MS spectra were visualized and analyzed with the FlexAnalysis 3.4 software from Bruker Daltonik. The instrument was calibrated with the peptide calibration standard II from Bruker Daltonik ranging from 700 Da to 3,200 Da (bradykinins 1 to 7, angiotensin I, angiotensin II, substance P, bombesin, renin substrate, adrenocorticotropic hormone (ACTH) (1-17) corticotropin-like intermediate peptide (CLIP), ACTH (18-39) CLIP, and somatostatin).

### (ii) HPLC-MS.

Analyses were performed in cell-free supernatant by using an Acquity H-class HPLC system coupled with a Synapt G2-Si-HESI-quadrupole TOF mass spectrometer (Waters, MA) and piloted by the MassLynx 4.1 software (Waters). For each experiment, 10-μL volumes of samples were injected in a reverse-phase Uptisphere C_18_ column (5 μm, 250 by 3.0 mm; Interchim, Monluçon, France). Molecule elution was carried out with a gradient of solvent A (water acidified with 0.1% [vol/vol] formic acid) and solvent B (acetonitrile acidified with 0.1% [vol/vol] formic acid) at a constant flow rate of 0.6 mL/min (5% solvent B for 5 min, 5% to 100% solvent B over 40 min, and 100% solvent B for 5 min). MS analyses of eluted molecules were carried out in positive ionization, centroid, and sensitive modes with the following parameters: source temperature, 150°C; capillary voltage, 3 kV; and desolvation gas (N_2_) temperature and flow, 300°C and 600 L/h, respectively. MS-MS measurements were realized with the fast data-dependent analysis (DDA) mode in the range of *m/z* 200 to 1,500. Fragmentation was performed by collision-induced dissociation with ramp energies from 10 to 15 V for low-molecular-weight ions and 20 to 100 V for high-molecular-weight ions.

### DNA extraction and sequencing.

The whole genomes of 47 Pseudomonas strains distributed in the 13 phylogroups of the P. syringae complex were sequenced. The 47 strains are listed in Table S2. DNA extraction of bacteria was carried out with the Promega Wizard genomic DNA purification kit (Promega, Madison, WI) according to the manufacturer protocol dedicated to Gram-negative bacterial strains. Whole-genome sequencing was performed by the MicrobesNG company (Birmingham, United Kingdom) with a 30× covering. Briefly, genomic DNA libraries are prepared with the Nextera XT library prep kit (Illumina, San Diego, CA) and then sequenced with the Illumina HiSeq to obtain 250-bp paired sequences. A *de novo* assembly of bacterial genomes was then realized with SPAdes 3.7 software (54). The 47 strains of interest were sequenced with minimal 40× coverage. Genomes with a mean size of 5,918,703 bp were assembled in 12 to 305 contigs with *N*_50_s ranging from 3,235,309 bp to 44,401 bp and with *L*_50_s ranging from 1 to 43 contigs. Lipopeptide BGCs were detected in 37 out of the 47 sequenced genomes; they were fully assembled except the peptin BGCs of three P. syringae strains, CMO0085, TA0018, and USA0050, but this did not affect the amino acid predictions.

### Bioinformatics tools for genome annotation and lipopeptide structure prediction.

Genome sequences were submitted to the antiSMASH 5.0 software (55) to identify lipopeptide BGCs through the detection of signature sequences of NRPS domains (C, A, T, and Te). The peptide sequence of lipopeptides was predicted by analysis of the A domain using the Stachelhaus code (56) and the NRPSPredictor2 program (57) that are both included in the antiSMASH 5.0 software. The d/l configuration of AA was predicted by analysis of C domains (Cstarter, ^L^C_L_, and C/E) (58) using Natural Product Domain Seeker (NaPDoS) software (59). For each strain containing lipopeptide BGCs, we checked for the presence of *sfp*-like gene in the sequenced genomes to ensure that nonribosomal synthesis could be functional. We detected the presence of the *sfp* gene encoding the phosphopantetheine transferase, and the length of the translated sequence was compatible with a functional phosphopantetheine transferase even with the 10 nonproducing strains.

### Data availability.

The mass spectrometry data related to this study are deposited in the repository MassIVE (https://massive.ucsd.edu). MS data are available under the MassIVE accession number MSV000087867, and MS-MS data are available under the MassIVE accession number MSV000089985. All DNA sequences are deposited in the GenBank database; accession numbers are listed in Table S2.

## References

[1] Bekiesch P, Zehl M, Domingo-Contreras E, Martín J, Pérez-Victoria I, Reyes F, Kaplan A, Rückert C, Busche T, Kalinowski J, Zotchev SB. 2020. Viennamycins: lipopeptides produced by a *Streptomyces* sp. J Nat Prod 83:2381–2389. doi:10.1021/acs.jnatprod.0c00152. 32786880 PMC7460545

[2] Esmaeel Q, Pupin M, Kieu NP, Chataigné G, Béchet M, Deravel J, Krier F, Höfte M, Jacques P, Leclère V. 2016. *Burkholderia* genome mining for nonribosomal peptide synthetases reveals a great potential for novel siderophores and lipopeptides synthesis. Microbiologyopen 5:512–526. doi:10.1002/mbo3.347. 27060604 PMC4906002

[3] Kaspar F, Neubauer P, Gimpel M. 2019. Bioactive secondary metabolites from *Bacillus subtilis*: a comprehensive review. J Nat Prod 82:2038–2053. doi:10.1021/acs.jnatprod.9b00110. 31287310

[4] Götze S, Stallforth P. 2020. Structure, properties, and biological functions of nonribosomal lipopeptides from pseudomonads. Nat Prod Rep 37:29–54. doi:10.1039/c9np00022d. 31436775

[5] Abdel-Aziz MM, Al-Omar MS, Mohammed HA, Emam TM. 2020. *In vitro* and *ex vivo* antibiofilm activity of a lipopeptide biosurfactant produced by the entomopathogenic *Beauveria bassiana* strain against *Microsporum canis*. Microorganisms 8:232. doi:10.3390/microorganisms8020232. 32050410 PMC7074774

[6] Hüttel W. 2021. Echinocandins: structural diversity, biosynthesis, and development of antimycotics. Appl Microbiol Biotechnol 105:55–66. doi:10.1007/s00253-020-11022-y. 33270153 PMC7778625

[7] Girard L, Höfte M, De Mot R. 2020. Lipopeptide families at the interface between pathogenic and beneficial *Pseudomonas*-plant interactions. Crit Rev Microbiol 46:397–419. doi:10.1080/1040841X.2020.1794790. 32885723

[8] Berge O, Monteil CL, Bartoli C, Chandeysson C, Guilbaud C, Sands DC, Morris CE. 2014. A user’s guide to a data base of the diversity of *Pseudomonas syringae* and its application to classifying strains in this phylogenetic complex. PLoS One 9:e105547. doi:10.1371/journal.pone.0105547. 25184292 PMC4153583

[9] Hall SJ, Dry IB, Gopurenko D, Whitelaw-Weckert MA. 2019. *Pseudomonas syringae* pv. *syringae* from cool climate Australian grapevine vineyards: new phylogroup PG02f associated with bacterial inflorescence rot. Plant Pathol 68:312–322. doi:10.1111/ppa.12936.

[10] Morris CE, Monteil CL, Berge O. 2013. The life history of *Pseudomonas syringae*: linking agriculture to earth system processes. Annu Rev Phytopathol 51:85–104. doi:10.1146/annurev-phyto-082712-102402. 23663005

[11] Xin X, Kvitko B, He SY. 2018. *Pseudomonas syringae*: what it takes to be a pathogen. Nat Rev Microbiol 16:316–328. doi:10.1038/nrmicro.2018.17. 29479077 PMC5972017

[12] Huang C, Pauwelyn E, Ongena M, Debois D, Leclère V, Jacques P, Bleyaert P, Höfte M. 2015. Characterization of cichopeptins, new phytotoxic cyclic lipodepsipeptides produced by *Pseudomonas cichorii* SF1-54 and their role in bacterial midrib rot disease of lettuce. Mol Plant Microbe Interact 28:1009–1022. doi:10.1094/MPMI-03-15-0061-R. 25961750

[13] Berti AD, Greve NJ, Christensen QH, Thomas MG. 2007. Identification of a biosynthetic gene cluster and the six associated lipopeptides involved in swarming motility of *Pseudomonas syringae* pv. *tomato* DC3000. J Bacteriol 189:6312–6323. doi:10.1128/JB.00725-07. 17601782 PMC1951903

[14] Burch AY, Zeisler V, Yokota K, Schreiber L, Lindow SE. 2014. The hygroscopic biosurfactant syringafactin produced by *Pseudomonas syringae* enhances fitness on leaf surfaces during fluctuating humidity. Environ Microbiol 16:2086–2098. doi:10.1111/1462-2920.12437. 24571678

[15] Pauwelyn E, Huang C, Ongena M, Leclère V, Jacques P, Bleyaert P, Budzikiewicz H, Schäfer M, Höfte M. 2013. New linear lipopeptides produced by *Pseudomonas cichorii* SF1-54 are involved in virulence, swarming motility, and biofilm formation. Mol Plant Microbe Interact 26:585–598. doi:10.1094/MPMI-11-12-0258-R. 23405865

[16] Götze S, Arp J, Lackner G, Zhang S, Kries H, Klapper M, Garcia-Altares M, Willing K, Günther M, Stallforth P. 2019. Structure elucidation of the syringafactin lipopeptides provides insight in the evolution of nonribosomal peptide synthetases. Chem Sci 10:10979–10990. doi:10.1039/c9sc03633d. 32953002 PMC7472662

[17] Morris CE, Lamichhane JR, Nikolić I, Stanković S, Moury B. 2019. The overlapping continuum of host range among strains in the *Pseudomonas syringae* complex. Phytopathol Res 1:4. doi:10.1186/s42483-018-0010-6.

[18] Hernandez MN, Lindow SE. 2019. *Pseudomonas syringae* increases water availability in leaf microenvironments via production of hygroscopic syringafactin. Appl Environ Microbiol 85:e01014-19. doi:10.1128/AEM.01014-19. 31285194 PMC6715840

[19] Bartoli C, Berge O, Monteil CL, Guilbaud C, Balestra GM, Varvaro L, Jones C, Dangl JL, Baltrus DA, Sands DC, Morris CE. 2014. The *Pseudomonas viridiflava* phylogroups in the *P syringae* species complex are characterized by genetic variability and phenotypic plasticity of pathogenicity-related traits. Environ Microbiol 16:2301–2315. doi:10.1111/1462-2920.12433. 24612372

[20] Price NPJ, Rooney AP, Swezey JL, Perry E, Cohan FM. 2007. Mass spectrometric analysis of lipopeptides from *Bacillus* strains isolated from diverse geographical locations. FEMS Microbiol Lett 271:83–89. doi:10.1111/j.1574-6968.2007.00702.x. 17419767

[21] Mora I, Cabrefiga J, Montesinos E. 2015. Cyclic lipopeptide biosynthetic genes and products, and inhibitory activity of plant-associated *Bacillus* against phytopathogenic bacteria. PLoS One 10:e0127738. doi:10.1371/journal.pone.0127738. 26024374 PMC4449161

[22] De Bruijn I, De Kock MJD, De Waard P, Van Beek TA, Raaijmakers JM. 2008. Massetolide A biosynthesis in *Pseudomonas fluorescens*. J Bacteriol 190:2777–2789. doi:10.1128/JB.01563-07. 17993540 PMC2293227

[23] Dubern J-F, Coppoolse ER, Stiekema WJ, Bloemberg GV. 2008. Genetics and functional characterization of the gene cluster directing the biosynthesis of putisolvin I and II in *Pseudomonas putida* strain PCL1445. Microbiology (Reading) 154:2070–2083. doi:10.1099/mic.0.2008/016444-0. 18599835

[24] Ma Z, Geudens N, Kieu NP, Sinnaeve D, Ongena M, Martins JC, Höfte M. 2016. Biosynthesis, chemical structure, and structure-activity relationship of orfamide lipopeptides produced by *Pseudomonas protegens* and related species. Front Microbiol 7:382. doi:10.3389/fmicb.2016.00382. 27065956 PMC4811929

[25] Penner D, DeVay JE, Backman P. 1969. The influence of syringomycin on ribonucleic synthesis. Plant Physiol 44:806–808. doi:10.1104/pp.44.6.806. 5817186 PMC396167

[26] Segre A, Bachmann RC, Ballio A, Bossa F, Grgurina I, Iacobellis NS, Marino G, Pucci P, Simmaco M, Takemoto J. 1989. The structure of syringomycins A1, E and G. FEBS Lett 255:27–31. doi:10.1016/0014-5793(89)81054-3. 2676599

[27] Scaloni A, Bachmann RC, Takemoto JY, Barra D, Simmaco M, Ballio A. 1994. Stereochemical structure of syringomycin, a phytotoxic metabolite of *Pseudomonas syringae* pv. *syringae*. Nat Prod Lett 4:159–164. doi:10.1080/10575639408043899.

[28] Gross DC, De Vay JE, Stadtman FH. 1977. Chemical properties of syringomycin and syringotoxin: toxigenic peptides produced by *Pseudomonas syringae*. J Appl Bacteriol 43:453–463. doi:10.1111/j.1365-2672.1977.tb00772.x.

[29] Ballio A, Bossa F, Collina A, Gallo M, Iacobellis NS, Paci M, Pucci P, Scaloni A, Segre A, Simmaco M. 1990. Structure of syringotoxin, a bioactive metabolite of *Pseudomonas syringae* pv. *syringae*. FEBS Lett 269:377–380. doi:10.1016/0014-5793(90)81197-v. 2401362

[30] Fukuchi N, Isogai A, Nakayama J, Takayama S, Yamashita S, Suyama K, Suzuki A. 1992. Isolation and structural elucidation of syringostatins, phytotoxins produced by *Pseudomonas syringae pv. syringae* Lilac isolate. J Chem Soc Perkin 1992:875–880.

[31] Isogai A, Fukuchi N, Yamashita S, Suyama K, Suzuki A. 1989. Syringostatins, novel phytotoxins produced by *Pseudomonas syringae* pv. *syringae*. Agric Biol Chem 53:3117–3119. doi:10.1271/bbb1961.53.3117.

[32] Harrison L, Teplow DB, Rinaldi M, Strobel G. 1991. Pseudomycins, a family of novel peptides from *Pseudomonas syringae* possessing broad-spectrum antifungal activity. J Gen Microbiol 137:2857–2865. doi:10.1099/00221287-137-12-2857. 1791440

[33] Ballio A, Bossa F, Di Giorgio D, Ferranti P, Paci M, Pucci P, Scaloni A, Segre A, Strobel GA. 1994. Novel bioactive lipodepsipeptides from *Pseudomonas syringae*: the pseudomycins. FEBS Lett 355:96–100. doi:10.1016/0014-5793(94)01179-6. 7957970

[34] Michelsen CF, Watrous J, Glaring MA, Kersten R, Koyama N, Dorrestein PC, Stougaard P. 2015. Nonribosomal peptides, key biocontrol components for *Pseudomonas fluorescens* In5, isolated from a Greenlandic suppressive soil. mBio 6:e00079-15. doi:10.1128/mBio.00079-15. 25784695 PMC4453515

[35] Ballio A, Barra D, Bossa F, Collina A, Grgurina I, Marino G, Moneti G, Paci M, Pucci P, Segre A, Simmaco M. 1991. Syringopeptins, new phytotoxic lipodepsipeptides of *Pseudomonas syringae* pv. *syringae*. FEBS Lett 291:109–112. doi:10.1016/0014-5793(91)81115-O. 1936237

[36] Grgurina I, Bensaci M, Pocsfalvi G, Mannina L, Cruciani O, Fiore A, Fogliano V, Sorensen KN, Takemoto JY. 2005. Novel cyclic lipodepsipeptide from *Pseudomonas syringae* pv. *lachrymans* strain 508 and syringopeptin antimicrobial activities. Antimicrob Agents Chemother 49:5037–5045. doi:10.1128/AAC.49.12.5037-5045.2005. 16304170 PMC1315969

[37] Komatsu H, Shirakawa T, Uchiyama T, Hoshino T. 2019. Chemical structure of cichorinotoxin, a cyclic lipodepsipeptide that is produced by *Pseudomonas cichorii* and causes varnish spots on lettuce. Beilstein J Org Chem 15:299–309. doi:10.3762/bjoc.15.27. 30800180 PMC6369977

[38] Grgurina I, Mariotti F, Fogliano V, Gallo M, Scaloni A, Iacobellis NS, Lo Cantore P, Mannina L, Van Axel Castelli V, Greco ML, Graniti A. 2002. A new syringopeptin produced by bean strains of *Pseudomonas syringae* pv. *syringae*. Biochim Biophys Acta 1597:81–89. doi:10.1016/s0167-4838(02)00283-2. 12009406

[39] Isogai A, Iguchi H, Nakayama J, Kusai A, Takemoto JY, Suzuki A. 1995. Structural analysis of new syringopeptins by tandem mass spectrometry. Biosci Biotechnol Biochem 59:1374–1376. doi:10.1271/bbb.59.1374. 7670202

[40] Scaloni A, Camoni L, Di Giorgio D, Scortichini M, Cozzolino R, Ballio A. 1997. A new syringopeptin produced by a *Pseudomonas syringae* pv. *syringae* strain isolated from diseased twigs of laurel. Physiol Mol Plant Pathol 51:259–264. doi:10.1006/pmpp.1997.0124.

[41] Fiore A, Mannina L, Sobolev AP, Salzano AM, Scaloni A, Grgurina I, Fullone MR, Gallo M, Swasey C, Fogliano V, Takemoto JY. 2008. Bioactive lipopeptides of ice-nucleating snow bacterium *Pseudomonas syringae* strain 31R1. FEMS Microbiol Lett 286:158–165. doi:10.1111/j.1574-6968.2008.01247.x. 18789127

[42] Lavermicocca P, Sante Iacobellis N, Simmaco M, Graniti A. 1997. Biological properties and spectrum of activity of *Pseudomonas syringae* pv. *syringae* toxins. Physiol Mol Plant Pathol 50:129–140. doi:10.1006/pmpp.1996.0078.

[43] Gerard J, Lloyd R, Barsby T, Haden P, Kelly MT, Andersen RJ. 1997. Massetolides A-H, antimycobacterial cyclic depsipeptides produced by two pseudomonads isolated from marine habitats. J Nat Prod 60:223–229. doi:10.1021/np9606456. 9157190

[44] D’aes J, De Maeyer K, Pauwelyn E, Höfte M. 2010. Biosurfactants in plant-*Pseudomonas* interactions and their importance to biocontrol. Environ Microbiol Rep 2:359–372. doi:10.1111/j.1758-2229.2009.00104.x. 23766108

[45] Geudens N, Martins JC. 2018. Cyclic lipodepsipeptides from *Pseudomonas* spp.—biological Swiss-Army knives. Front Microbiol 9:1867. doi:10.3389/fmicb.2018.01867. 30158910 PMC6104475

[46] Dillon MM, Thakur S, Almeida RND, Wang PW, Weir BS, Guttman DS. 2019. Recombination of ecologically and evolutionarily significant loci maintains genetic cohesion in the *Pseudomonas syringae* species complex. Genome Biol 20:3. doi:10.1186/s13059-018-1606-y. 30606234 PMC6317194

[47] Morris CE, Moury B. 2019. Revisiting the concept of host range of plant pathogens. Annu Rev Phytopathol 57:63–90. doi:10.1146/annurev-phyto-082718-100034. 31082307

[48] Bender CL, Alarcón-Chaidez F, Gross DC. 1999. *Pseudomonas syringae* phytotoxins: mode of action, regulation, and biosynthesis by peptide and polyketide synthetases. Microbiol Mol Biol Rev 63:266–292. doi:10.1128/MMBR.63.2.266-292.1999. 10357851 PMC98966

[49] Demba Diallo M, Monteil CL, Vinatzer BA, Clarke CR, Glaux C, Guilbaud C, Desbiez C, Morris CE. 2012. *Pseudomonas syringae* naturally lacking the canonical type III secretion system are ubiquitous in nonagricultural habitats, are phylogenetically diverse and can be pathogenic. ISME J 6:1325–1335. doi:10.1038/ismej.2011.202. 22237542 PMC3379638

[50] Lindow SE, Brandl MT. 2003. Microbiology of the phyllosphere. Appl Environ Microbiol 69:1875–1883. doi:10.1128/AEM.69.4.1875-1883.2003. 12676659 PMC154815

[51] Baltrus DA, Nishimura MT, Romanchuk A, Chang JH, Mukhtar MS, Cherkis K, Roach J, Grant SR, Jones CD, Dangl JL. 2011. Dynamic evolution of pathogenicity revealed by sequencing and comparative genomics of 19 *Pseudomonas syringae* isolates. PLoS Pathog 7:e1002132. doi:10.1371/journal.ppat.1002132. 21799664 PMC3136466

[52] Hockett KL, Nishimura MT, Karlsrud E, Dougherty K, Baltrus DA. 2014. *Pseudomonas syringae* CC1557: a highly virulent strain with an unusually small type III effector repertoire that includes a novel effector. Mol Plant Microbe Interact 27:923–932. doi:10.1094/MPMI-11-13-0354-R. 24835253

[53] Gross DC. 1985. Regulation of syringomycin synthesis in *Pseudomonas syringae* pv. *syringae* and defined conditions for its production. J Appl Bacteriol 58:167–174. doi:10.1111/j.1365-2672.1985.tb01444.x. 3980301

[54] Bankevich A, Nurk S, Antipov D, Gurevich AA, Dvorkin M, Kulikov AS, Lesin VM, Nikolenko SI, Pham S, Prjibelski AD, Pyshkin AV, Sirotkin AV, Vyahhi N, Tesler G, Alekseyev MA, Pevzner PA. 2012. SPAdes: a new genome assembly algorithm and its applications to single-cell sequencing. J Comput Biol 19:455–477. doi:10.1089/cmb.2012.0021. 22506599 PMC3342519

[55] Blin K, Shaw S, Steinke K, Villebro R, Ziemert N, Lee SY, Medema MH, Weber T. 2019. antiSMASH 5.0: updates to the secondary metabolite genome mining pipeline. Nucleic Acids Res 47:W81–W87. doi:10.1093/nar/gkz310. 31032519 PMC6602434

[56] Stachelhaus T, Mootz HD, Marahiel MA. 1999. The specificity-conferring code of adenylation domains in nonribosomal peptide synthetases. Chem Biol 6:493–505. doi:10.1016/S1074-5521(99)80082-9. 10421756

[57] Röttig M, Medema MH, Blin K, Weber T, Rausch C, Kohlbacher O. 2011. NRPSpredictor2—a web server for predicting NRPS adenylation domain specificity. Nucleic Acids Res 39:W362–W367. doi:10.1093/nar/gkr323. 21558170 PMC3125756

[58] Caradec T, Pupin M, Vanvlassenbroeck A, Devignes M-D, Smaïl-Tabbone M, Jacques P, Leclère V. 2014. Prediction of monomer isomery in florine: a workflow dedicated to nonribosomal peptide discovery. PLoS One 9:e85667. doi:10.1371/journal.pone.0085667. 24465643 PMC3897469

[59] Ziemert N, Podell S, Penn K, Badger JH, Allen E, Jensen PR. 2012. The natural product domain seeker NaPDoS: a phylogeny based bioinformatic tool to classify secondary metabolite gene diversity. PLoS One 7:e34064. doi:10.1371/journal.pone.0034064. 22479523 PMC3315503

