# Supplementary material for: The Diversity of Lipopeptides in the Pseudomonas syringae Complex Parallels Phylogeny and Sheds Light on Structural Diversification during Evolutionary History
Source: Microbiol Spectr. 2022 Oct 26;10(6):e01456-22. doi: 10.1128/spectrum.01456-22 (PMC9769872; doi:10.1128/spectrum.01456-22)
Supplement: Supplemental file 3 — Table S3 and Fig. S1 to S6. Download spectrum.01456-22-s0003.pdf, PDF file, 5.5 MB [file spectrum.01456-22-s0003.pdf]

Bricout et al. The diversity of lipopeptides in the *P. syringae* complex parallels phylogeny and sheds light on structural diversification during evolutionary history

Supplementary Table S3 : Classification and nomenclature proposed for *P. syringae* lipopeptides

Family	Subfamily	Lipopeptide (revised nomenclature)	Lipopeptide (synonym)	Calculated mass (Da)
Factins	Syringafactin	3-OH C10:0 syringafactin [Val6]	Syringafactin A	1081.74
		3-OH C10:0 syringafactin [Leu6]	Syringafactin B	1095.75
		3-OH C10:0 syringafactin [Ile6]	Syringafactin C	1095.75
		3-OH C12:0 syringafactin [Val6]	Syringafactin D	1109.77
		3-OH C12:0 syringafactin [Leu6]	Syringafactin E	1123.78
		3-OH C12:0 syringafactin [Ile6]	Syringafactin F	1123.78
	Cichofactin	3-OH C10:0 cichofactin [Val6]	Cichofactin A	1108.75
		3-OH C12:1 cichofactin [Val6]		1134.76
		3-OH C12:0 cichofactin [Val6]	Cichofactin B	1136.75
		3-OH C14:1 cichofactin [Val6]		1162.79
		3-OH C10:0 cichofactin [Leu6]		1122.76
		3-OH C10:0 cichofactin [Ile6]		1122.76
		3-OH C12:0 cichofactin [Leu6]		1150.79
		3-OH C12:0 cichofactin [Ile6]		1150.79
		3-OH C14:0 cichofactin [Leu6]		1178.83
		3-OH C14:0 cichofactin [Ile6]		1178.83
Mycins	Syringotoxin	3-OH C14:0 syringotoxin	Syringotoxin B	1135.55
		3,4-OH C14:0 syringotoxin		1151.55
		3-OH C16:0 syringotoxin		1163.58
		3,4-OH C16:0 syringotoxin		1179.58
	Syringostatin	C14:0 syringostatin		1162.60
		3-OH C14:0 syringostatin	Syringostatin A	1178.59
		3,4-OH C14:0 syringostatin	Syringostatin B	1194.59
		3-OH C16:0 syringostatin		1206.63
		3,4-OH C16:0 syringostatin		1222.62
	Syringomycin	3-OH C10:0 syringomycin	Syringomycin A1	1196.56
		C12:0 syringomycin		1208.60
		3-OH C12:0 syringomycin	Syringomycin E	1224.59
		C14:0 syringomycin		1236.63
		3-OH C14:0 syringomycin	Syringomycin G	1252.62
		3-OH C16:0 syringomycin		1280.65
	Pseudomycin	3-OH C14:0 pseudomycin	Pseudomycin B	1206.59
		3,4-OH C14:0 pseudomycin	Pseudomycin A	1222.58
		C16:0 pseudomycin		1218.63
		3-OH C16:0 pseudomycin	Pseudomycin C'	1234.62
		3,4-OH C16:0 pseudomycin	Pseudomycin C	1250.62
		3-OH C18:0 pseudomycin		1262.65
		3,4-OH C18:0 pseudomycin		1278.65

**Mycins**	**Syringomycin-2**	C16:1 syringomycin-2		1248.63
		3-OH C16:1 syringomycin-2		1264.62
		3,4-OH C16:1 syringomycin-2		1280.62
		3-OH C18:1 syringomycin-2		1292.65
	**Pseudomycin-2**	3-OH C16:0 pseudomycin-2		1262.65
		3-OH C18:0 pseudomycin-2		1290.68
**Peptins**	**Cichopeptin**	3-OH C12:1 cichopeptin [Leu22]	Cichopeptin A	2064.20
		3-OH C12:1 cichopeptin [Ile22]	Cichopeptin A	2064.20
		3-OH C12:1 cichopeptin [Val22]	Cichopeptin B	2050.18
	**Cichopeptin-2**	3-OH C10:0 cichopeptin-2		2010.15
	**Cichopeptin-3**	3-OH C12:1 cichopeptin-3		2076.19
	**Cichorinotoxin**	3-OH C10:0 cichorinotoxin	Cichorinotoxin	2068.19
	**Syringopeptin 22Phv**	3-OH C10:0 syringopeptin 22Phv	Syringopeptin 22phVA	2129.19
		3-OH C12:0 syringopeptin 22Phv	Syringopeptin 22phVB	2157.22
	**Syringopeptin 22**	3-OH C10:0 syringopeptin 22	Syringopeptin 22A	2143.20
		3-OH C12:0 syringopeptin 22	Syringopeptin 22B	2171.24
	**Syringopeptin 22-2**	3-OH C10:0 syringopeptin 22-2		2145.22
		3-OH C12:0 syringopeptin 22-2		2173.25
	**Syringopeptin 22-3**	3-OH C10:0 syringopeptin 22-3		2159.24
		3-OH C12:0 syringopeptin 22-3		2187.27
	**Syringopeptin SC**	3-OH C10:0 syringopeptin SC	Syringopeptin SC1	2157.22
		3-OH C12:0 syringopeptin SC	Syringopeptin SC2	2185.25
	**Syringopeptin 508**	3-OH C10:0 syringopeptin 508		2159.24
		3-OH C12:0 syringopeptin 508	Syringopeptin 508A	2187.27
		3-OH C14:0 syringopeptin 508	Syringopeptin 508B	2215.30
	**Syringopeptin 25**	3-OH C10:0 syringopeptin 25 [Tyr25]	Syringopeptin 25A	2398.36
		3-OH C12:0 syringopeptin 25 [Tyr25]	Syringopeptin 25B	2426.39
		3-OH C10:0 syringopeptin 25 [Phe25]	[Phe^{25}]-Syringopeptin 25A	2382.37
	**Syringopeptin 25-2**	3-OH C10:0 syringopeptin 25-2		2398.36
		3-OH C12:0 syringopeptin 25-2		2426.39
	**Syringopeptin 25-3**	3-OH C10:0 syringopeptin 25-3		2412.38
	**Syringopeptin 25-4**	3-OH C10:0 syringopeptin 25-4		2440.41
		3-OH C12:0 syringopeptin 25-4		2468.44
	**Syringopeptin 25-5**	3-OH C10:0 syringopeptin 25-5		2442.43
	**Syringopeptin 25-6**	3-OH C10:0 syringopeptin 25-6		2444.40
		3-OH C12:0 syringopeptin 25-6		2472.44

Supplementary Figure S1:

A) BGC organization and amino-acid sequences of the syringafactins of *P. syringae* CVB0040 predicted by bioinformatics analysis. They were the same for *P. syringae* FBP1392, CVB0031, T3W0028, UB0415, USA0087, CST0018, CC0301, UB303, USA011, USA0035, USA0050, CC1466, TA0005, TA0018, CCE0100, CCV0214, CMW0036, CST0002, LAB0081, CSZ0174, JD14, JD15, CFBP2067, CCE0075, CCE0114 and CSZ0296.

B) Fragmentation mass spectrum of the $[M+H]^+$ ion of m/z 1082.8 corresponding to syringafactin A (3-OH C10:0 syringafactin [Val6]) produced by *P. syringae* CVB0040.

C) Fragmentation mass spectrum of the $[M+H]^+$ ion of m/z 1096.8 corresponding to syringafactin B (3-OH C10:0 syringafactin [Leu6]) or syringafactin C (3-OH C10:0 syringafactin [Ile6]) produced by *P. syringae* CVB0040.

D) Fragmentation mass spectrum of the $[M+H]^+$ ion of m/z 1110.9 corresponding to syringafactin D (3-OH C12:0 syringafactin [Val6]) produced by *P. syringae* CVB0040 .

E) Fragmentation mass spectrum of the $[M+H]^+$ ion of m/z 1124.9 corresponding to syringafactin E (3-OH C12:0 syringafactin [Leu6]) or F (3-OH C12:0 syringafactin [Ile6]) produced by *P. syringae* CVB0040.

The fragmentation pattern of the $[M+H]^+$ ion of m/z 1082.8 and 1096.8 were very similar for *P. syringae* strains CVB0040, CVB0031, T3W0028, UB0415, USA0087, CST0018, USA011, USA0035, CCE0100, CCV0214, CST0002, CCE0075, CSZ0296.

The fragmentation pattern of the $[M+H]^+$ ion of m/z 1110.9 were very similar for *P. syringae* strains CVB0040, TA0018, CCE0100, CST0002, CCE0075 and CSZ0296.

The fragmentation pattern of the $[M+H]^+$ ion of m/z 1124.9 were very similar for *P. syringae* strains CVB0040, CST0002, CSZ0296.

b ions are indicated with red circles and arrows, y ions are indicated with blue circles and arrows

A

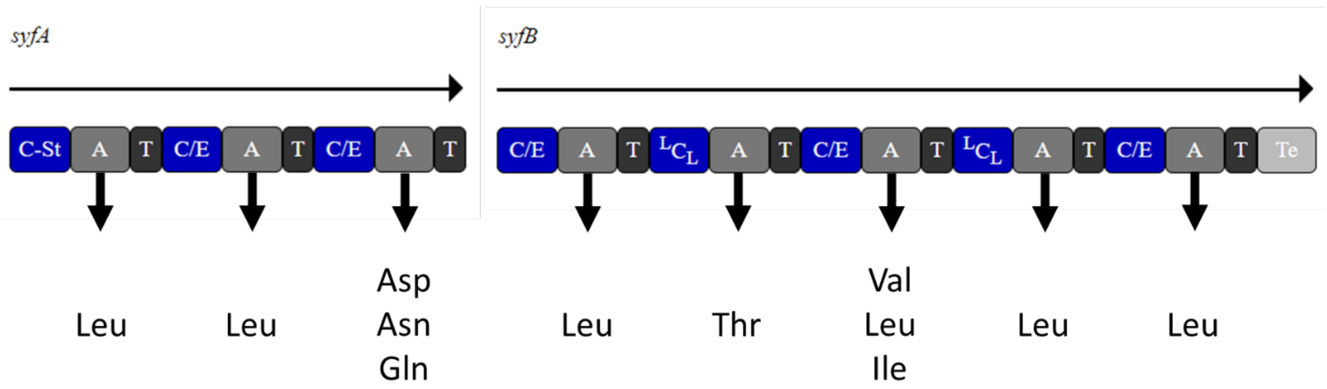

B

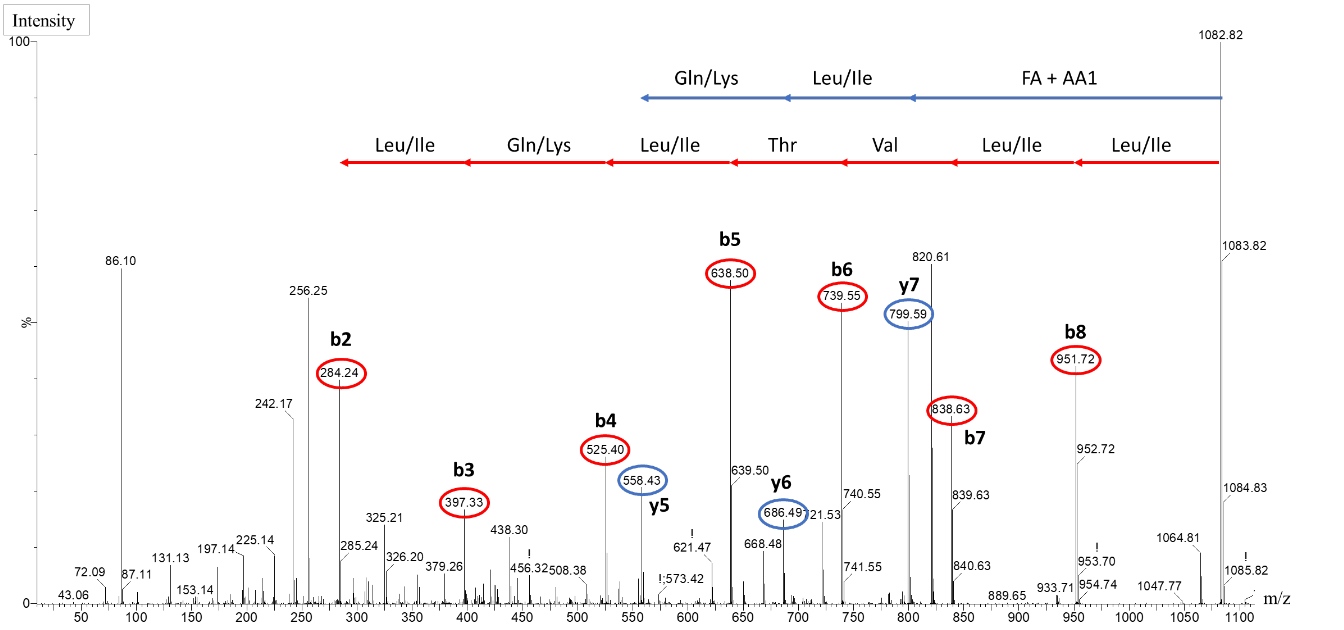

Intensity
Gln/Lys
Leu/Ile
FA + AA1
Leu/Ile
Gln/Lys
Leu/Ile
Thr
Val
Leu/Ile
Leu/Ile
b2
b3
b4
b5
b6
b7
b8
y5
y6
y7
284.24
397.33
525.40
558.43
638.50
686.49
739.55
799.59
838.63
951.72
1082.82
m/z

C

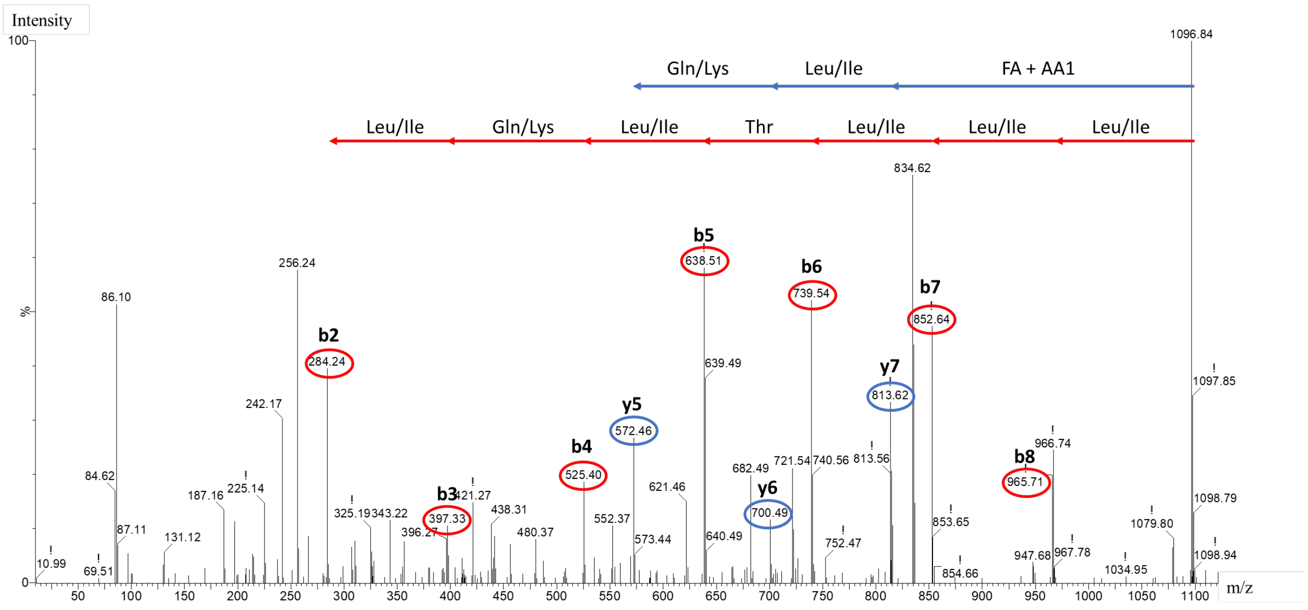

Intensity
Gln/Lys
Leu/Ile
FA + AA1
Leu/Ile
Gln/Lys
Leu/Ile
Thr
Leu/Ile
Leu/Ile
Leu/Ile
b2
b3
b4
b5
b6
b7
b8
y5
y6
y7
284.24
397.33
525.40
572.46
638.51
700.49
739.54
813.62
852.64
965.71
1096.84
m/z

D

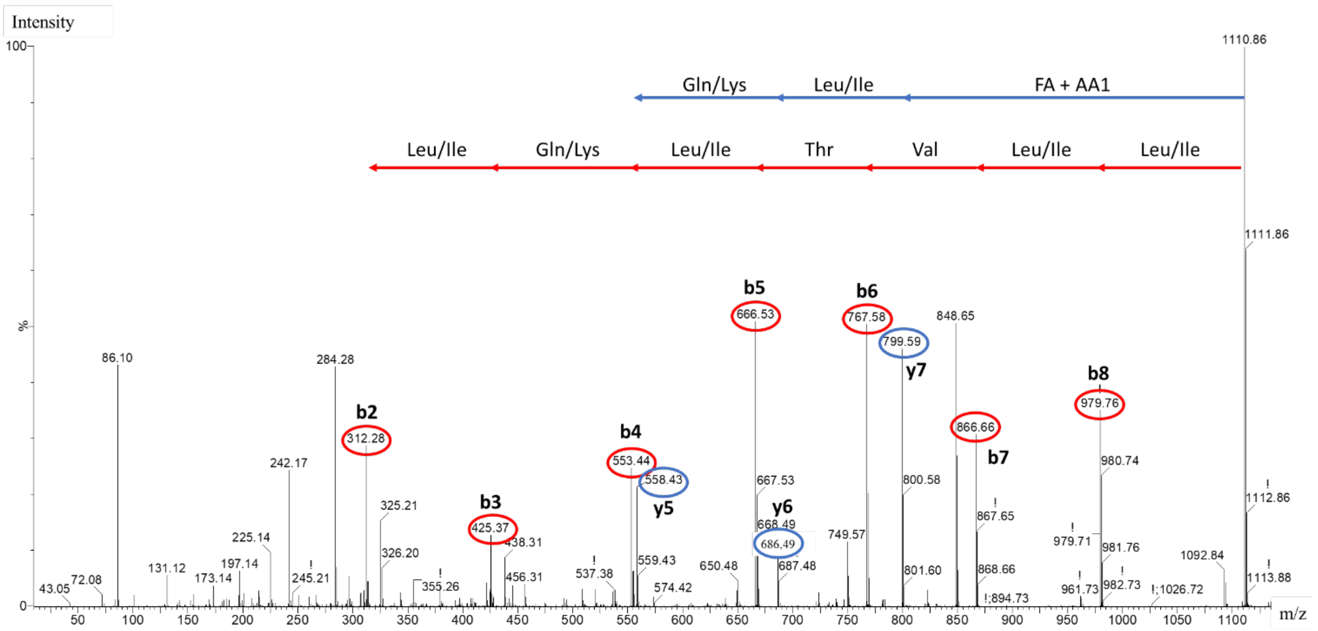
Intensity
100
%
0
m/z
Gln/Lys
Leu/Ile
FA + AA1
Leu/Ile
Gln/Lys
Leu/Ile
Thr
Val
Leu/Ile
Leu/Ile
b2
b3
b4
y5
b5
y6
b6
y7
b7
b8
1110.86
1111.86
1112.86
1113.88
1092.84
1026.72
982.73
981.76
980.74
979.76
979.71
961.73
894.73
868.66
867.65
866.66
848.65
801.60
800.58
799.59
767.58
749.57
687.48
686.49
668.49
667.53
666.53
650.48
574.42
559.43
558.43
553.44
537.38
456.31
438.31
425.37
355.26
326.20
325.21
312.28
284.28
245.21
242.17
225.14
197.14
173.14
131.12
86.10
72.08
43.05

E

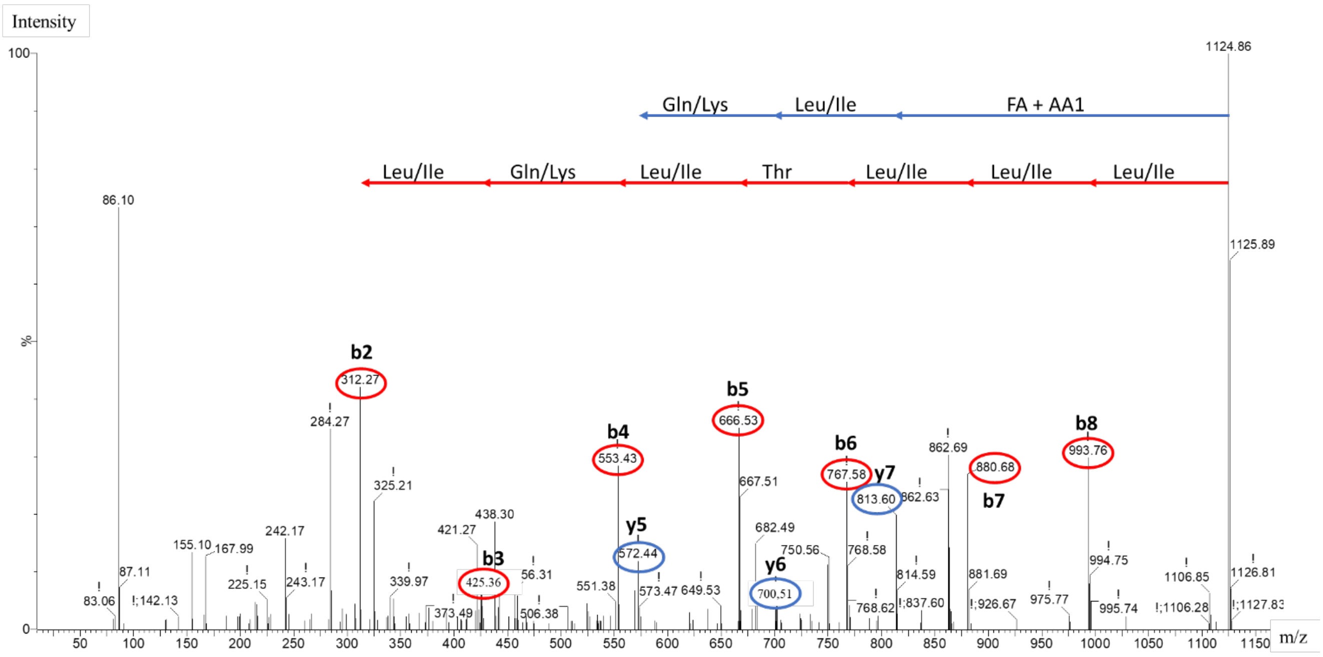
Intensity
100
%
0
m/z
Gln/Lys
Leu/Ile
FA + AA1
Leu/Ile
Gln/Lys
Leu/Ile
Thr
Leu/Ile
Leu/Ile
Leu/Ile
b2
b3
b4
y5
b5
y6
b6
y7
b7
b8
1124.86
1125.89
1126.81
1127.83
1106.85
1106.28
995.74
994.75
993.76
975.77
926.67
881.69
880.68
862.69
862.63
837.60
814.59
813.60
768.62
768.58
767.58
750.56
700.51
682.49
667.51
666.53
649.53
573.47
572.44
553.43
551.38
506.38
438.30
425.36
421.27
373.49
339.97
325.21
312.27
284.27
243.17
242.17
225.15
167.99
155.10
142.13
87.11
86.10
83.06

Supplementary Figure S2:

A) BGC organization and amino-acid sequences of the cichofactins of P. cichorii CFBP4407 predicted by bioinformatics analysis. They were the same for *P. syringae* CMO0085, CST0099, GAW0197, CEB0041, LAB0023, CMW0012, LNW0029 and CMW0010.

B) Fragmentation mass spectrum of the $[M+H]^+$ ion of m/z 1109.8 corresponding to cichofactin A (3-OH C10:0 cichofactin [Val6]) produced by *P. cichorii* CFBP4407.

C) Fragmentation mass spectrum of the $[M+H]^+$ ion of m/z 1137.9 corresponding to cichofactin B (3-OH C12:0 cichofactin [Val6]) produced by *P. cichorii* CFBP4407.

D) Fragmentation mass spectrum of the $[M+H]^+$ ion of m/z 1123.9 corresponding to 3-OH C10:0 cichofactin [Leu6] or 3-OH C10:0 cichofactin [Ile6] produced by *P. cichorii* CFBP4407.

E) Fragmentation mass spectrum of the $[M+H]^+$ ion of m/z 1151.9 corresponding to 3-OH C12:0 cichofactin [Leu6] or 3-OH C12:0 cichofactin [Ile6] produced by *P. cichorii* CFBP4407.

F) Fragmentation mass spectrum of the $[M+H]^+$ ion of m/z 1135.9 corresponding to 3-OH C12:1 cichofactin [Val6] produced by *P. cichorii* CFBP4407.

G) Fragmentation mass spectrum of the $[M+H]^+$ ion of m/z 1163.9 corresponding to 3-OH C14:1 cichofactin [Val6] produced by *P. cichorii* CFBP4407.

The fragmentation patterns of the $[M+H]^+$ ion of m/z 1109.8, 1137.9, 1123.9 and 1151.9 were very similar for *P. cichorii* strains CFBP4407, CST0099 and 83.1.
The fragmentation patterns of the $[M+H]^+$ ion of m/z 1135.9 and 1163.9 were very similar for *P. cichorii* CFBP4407 and 83.1.

b ions are indicated with red circles and arrows, y ions are indicated with blue circles and arrows

A

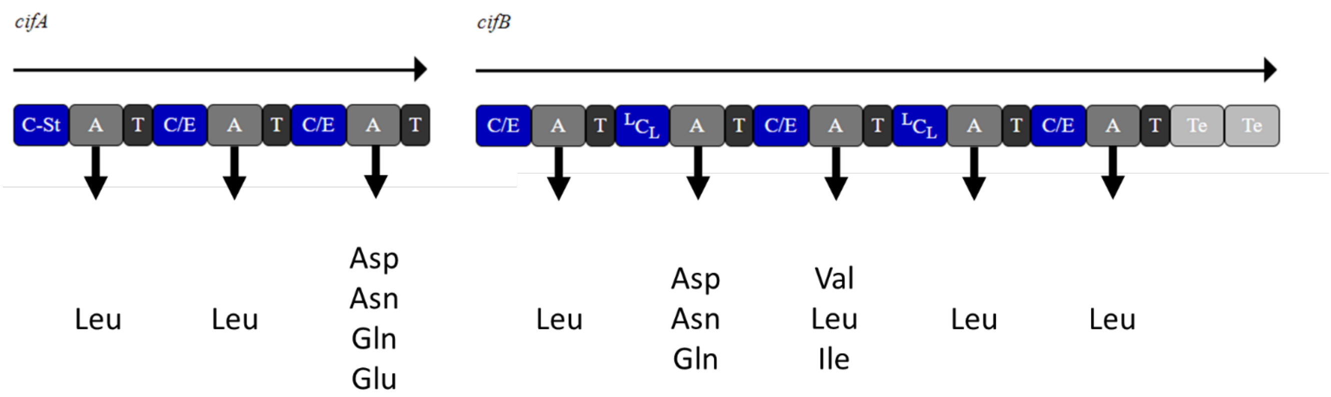

B

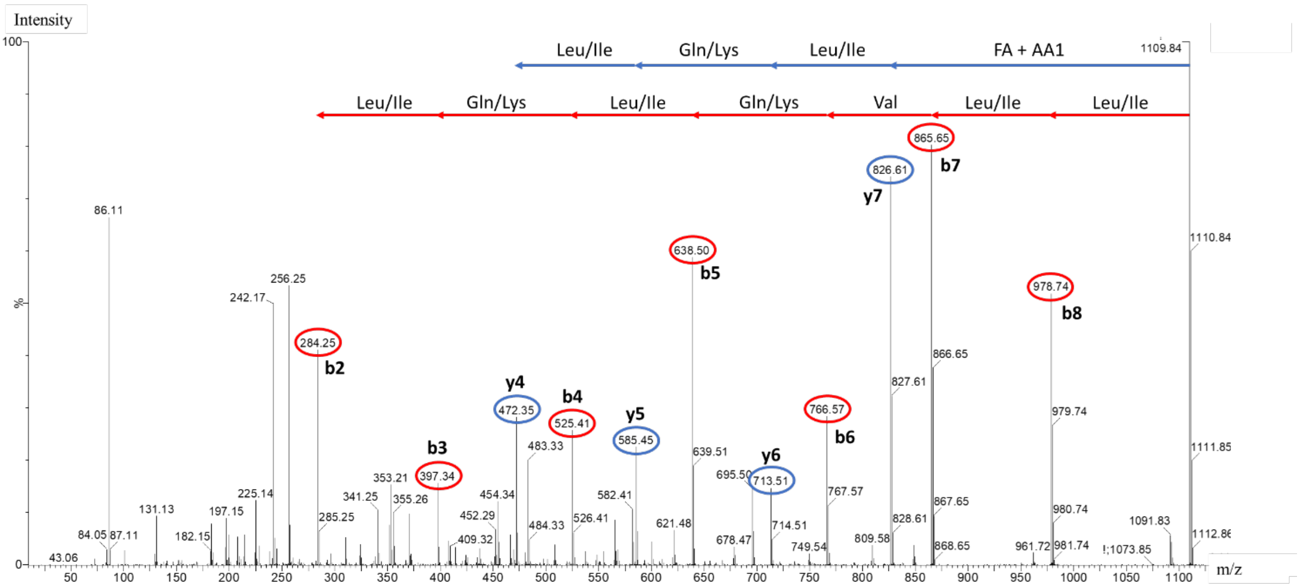
Intensity
100
%
0
m/z
Leu/Ile
Gln/Lys
Leu/Ile
FA + AA1
Leu/Ile
Gln/Lys
Leu/Ile
Gln/Lys
Val
Leu/Ile
Leu/Ile
1109.84
865.65
b7
826.61
y7
86.11
1110.84
638.50
b5
978.74
b8
256.25
242.17
284.25
b2
866.65
827.61
y4
472.35
b4
525.41
y5
585.45
766.57
b6
979.74
b3
397.34
y6
713.51
1111.85
483.33
639.51
353.21
695.50
225.14
341.25
355.26
454.34
582.41
767.57
867.65
131.13
197.15
452.29
526.41
980.74
285.25
484.33
621.48
714.51
828.61
1091.83
84.05
87.11
182.15
409.32
678.47
809.58
1112.86
749.54
868.65
961.72
981.74
1073.85
43.06

C

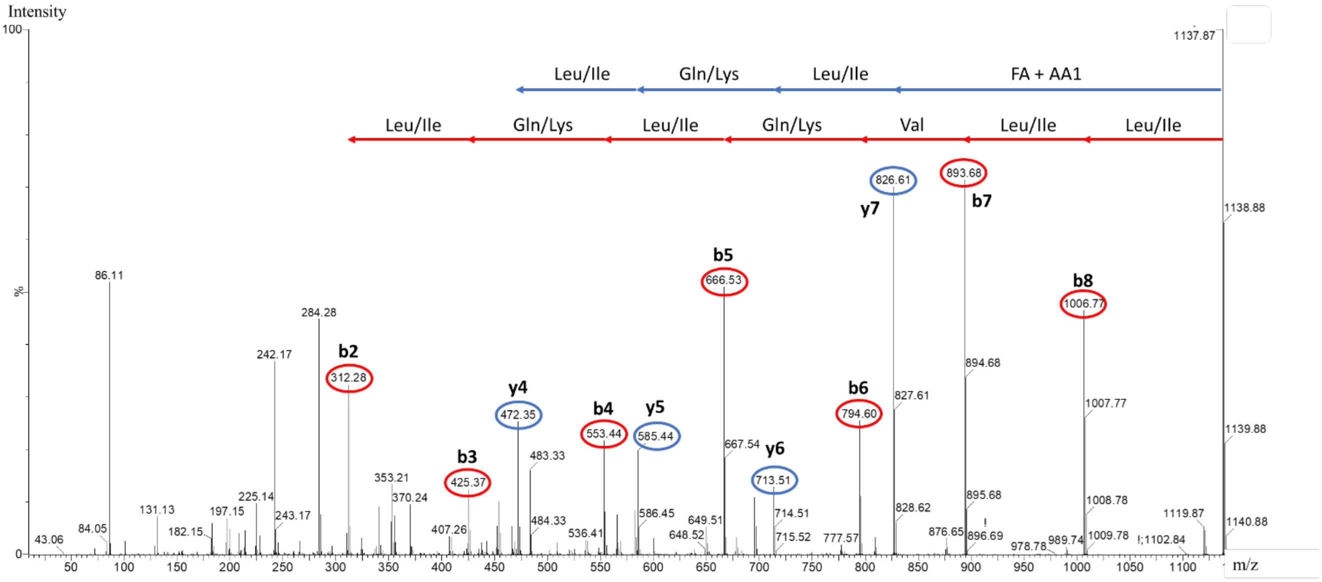
Intensity
100
%
0
m/z
1137.87
Leu/Ile
Gln/Lys
Leu/Ile
FA + AA1
Leu/Ile
Gln/Lys
Leu/Ile
Gln/Lys
Val
Leu/Ile
Leu/Ile
826.61
y7
893.68
b7
1138.88
b5
666.53
b8
1006.77
86.11
284.28
b2
312.28
242.17
894.68
y4
472.35
b6
794.60
827.61
1007.77
b4
553.44
y5
585.44
1139.88
483.33
667.54
y6
713.51
b3
425.37
353.21
370.24
225.14
895.68
1008.78
131.13
197.15
243.17
586.45
714.51
828.62
1119.87
1140.88
84.05
182.15
407.26
484.33
536.41
649.51
648.52
715.52
777.57
876.65
896.69
1009.78
43.06
978.78
989.74
1102.84

D

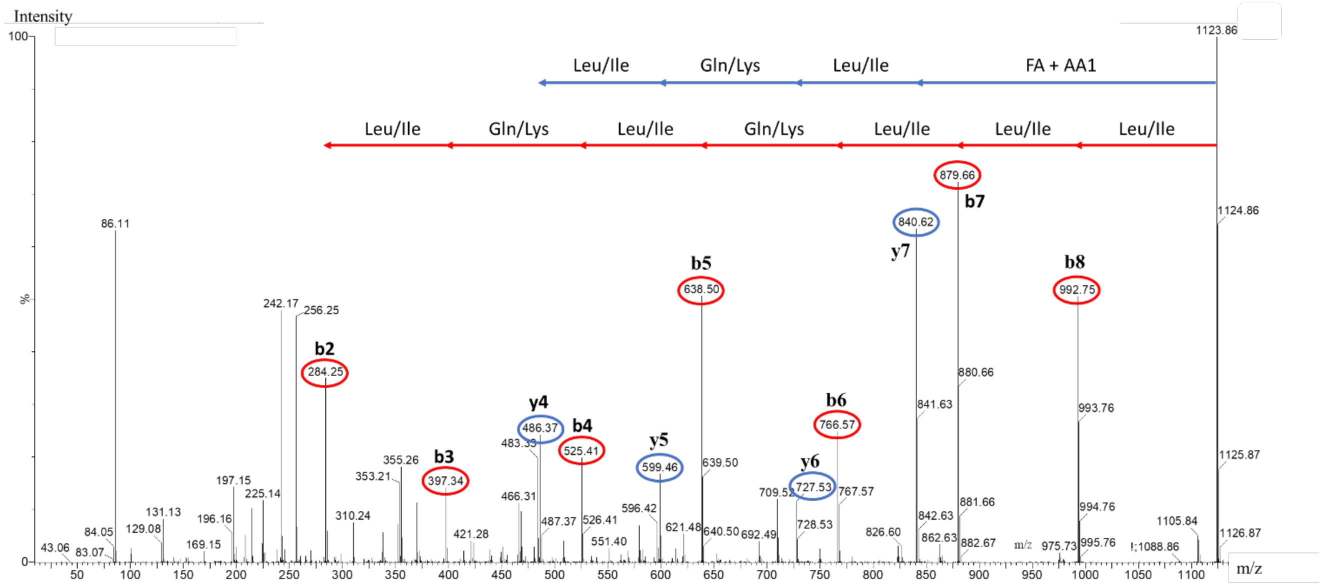
Intensity
%
m/z
Leu/Ile
Gln/Lys
Leu/Ile
FA + AA1
Leu/Ile
Gln/Lys
Leu/Ile
Gln/Lys
Leu/Ile
Leu/Ile
Leu/Ile
b2
284.25
b3
397.34
y4
486.37
b4
525.41
y5
599.46
b5
638.50
y6
727.53
b6
766.57
y7
840.62
b7
879.66
b8
992.75
1123.86
1124.86
86.11
242.17
256.25

E

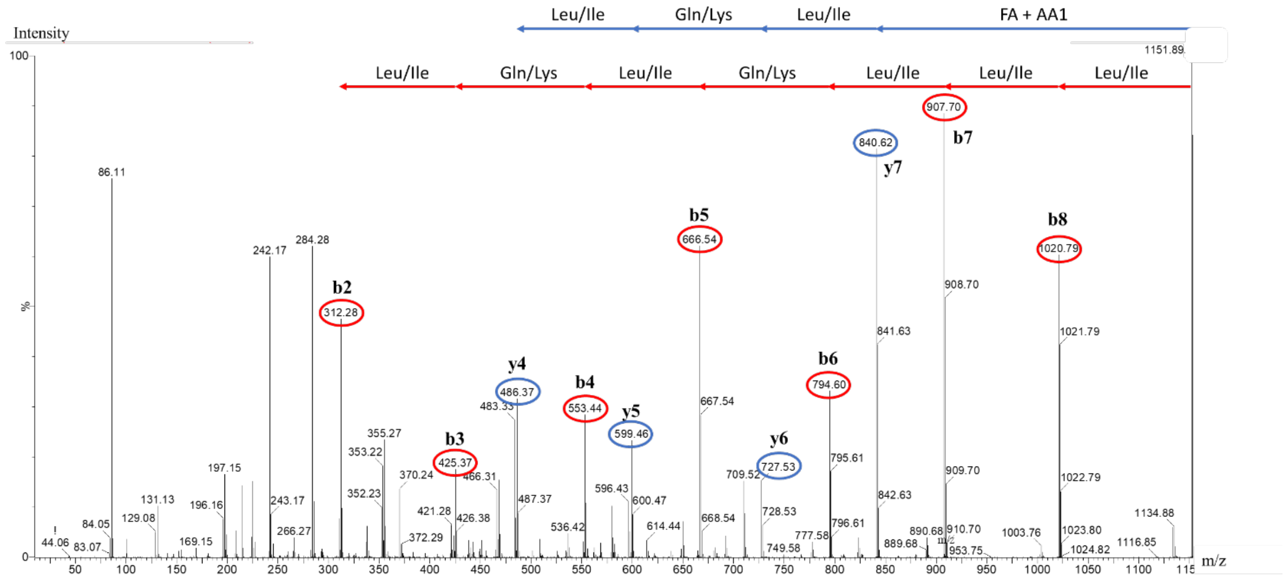
Intensity
%
m/z
Leu/Ile
Gln/Lys
Leu/Ile
FA + AA1
Leu/Ile
Gln/Lys
Leu/Ile
Gln/Lys
Leu/Ile
Leu/Ile
Leu/Ile
b2
312.28
b3
425.37
y4
486.37
b4
553.44
y5
599.46
b5
666.54
y6
727.53
b6
794.60
y7
840.62
b7
907.70
b8
1020.79
1151.89
86.11
242.17
284.28

F

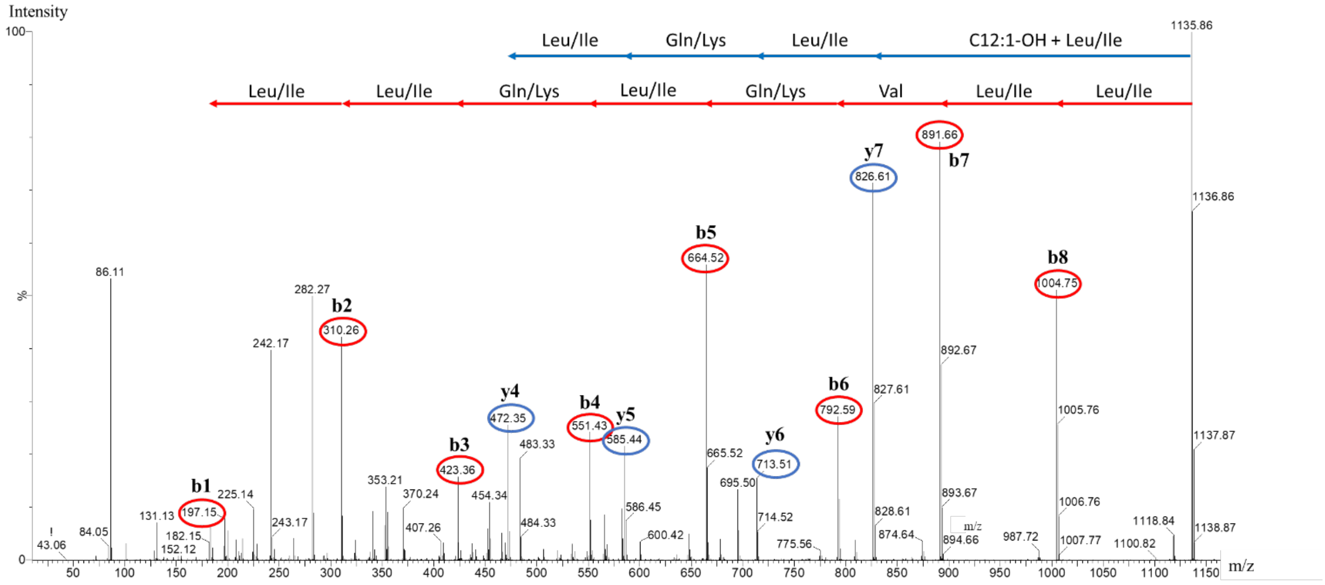

Intensity
100
%
0
1135.86
Leu/Ile
Gln/Lys
Leu/Ile
C12:1-OH + Leu/Ile
Leu/Ile
Leu/Ile
Gln/Lys
Leu/Ile
Gln/Lys
Val
Leu/Ile
Leu/Ile
891.66
b7
y7
826.61
1136.86
b5
664.52
b8
1004.75
86.11
282.27
b2
310.26
242.17
892.67
b6
792.59
827.61
y4
472.35
b4
551.43
y5
585.44
1005.76
483.33
b3
423.36
665.52
y6
713.51
1137.87
b1
197.15
225.14
353.21
370.24
454.34
695.50
586.45
714.52
828.61
893.67
1006.76
131.13
243.17
484.33
1118.84
84.05
182.15
152.12
43.06
407.26
600.42
775.56
874.64
894.66
987.72
1007.77
1100.82
1138.87
m/z

G

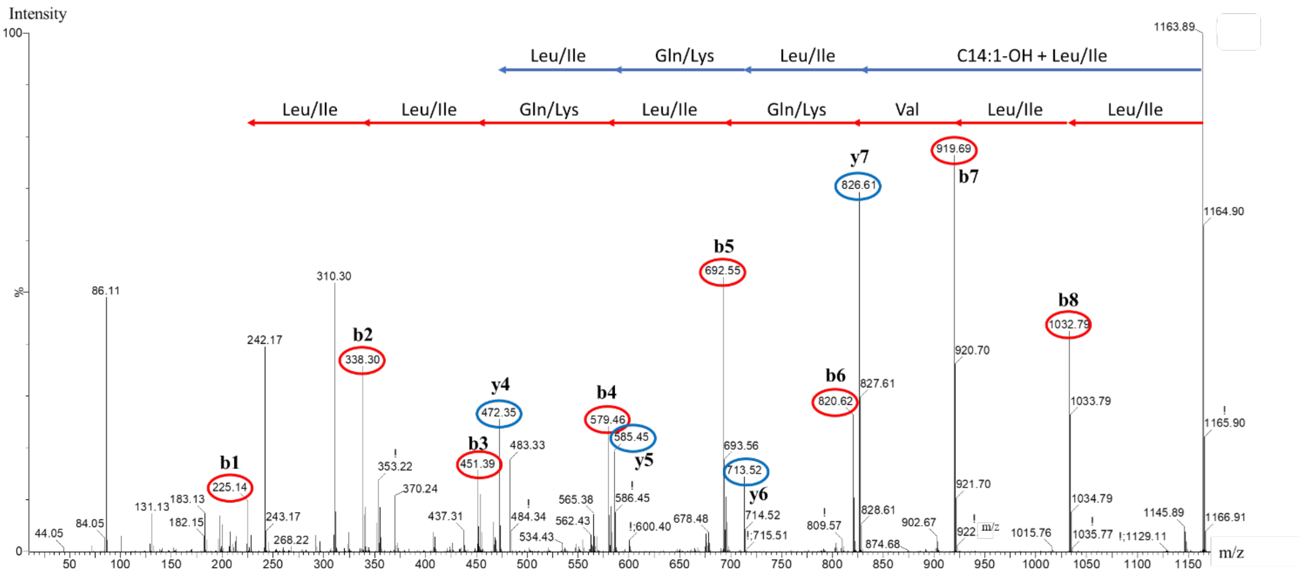

Intensity
100
%
0
1163.89
Leu/Ile
Gln/Lys
Leu/Ile
C14:1-OH + Leu/Ile
Leu/Ile
Leu/Ile
Gln/Lys
Leu/Ile
Gln/Lys
Val
Leu/Ile
Leu/Ile
919.69
b7
y7
826.61
1164.90
b5
692.55
310.30
86.11
b8
1032.79
242.17
b2
338.30
920.70
b6
820.62
827.61
1033.79
y4
472.35
b4
579.46
585.45
y5
483.33
b3
451.39
693.56
713.52
y6
353.22
370.24
b1
225.14
1165.90
921.70
183.13
131.13
182.15
84.05
44.05
243.17
268.22
437.31
484.34
534.43
565.38
562.43
586.45
600.40
678.48
714.52
715.51
809.57
828.61
874.68
902.67
922
1015.76
1034.79
1035.77
1129.11
1145.89
1166.91
m/z

Supplementary Figure S3:

A) BGC organization and amino-acid sequences of the syringotoxin of *P. syringae* CST0099 predicted by bioinformatics analysis. They were the same for *P. syringae* UB0415, USA0087, UB303, CMO0085, GAW0197, TA0005, TA0018 and CCV0214. Fragmentation mass spectrum of the $[M+H]^+$ ion of m/z 1136.6 corresponding to syringotoxin B (3-OH C14:0 syringotoxin) produced by *P. syringae* CST0099.

B) BGC organization and amino-acid sequences of the syringostatin of *P. syringae* CC1466 predicted by bioinformatics analysis. They were the same for *P. syringae* CFBP1392 and CVB0040. Fragmentation mass spectrum of the $[M+H]^+$ ion of m/z 1179.8 corresponding to syringostatin A (3-OH C14:0 syringostatin) produced by *P. syringae* CC1466.

C) BGC organization and amino-acid sequences of the syringomycin of *P. syringae* CVB0031 predicted by bioinformatics analysis. They were the same for *P. syringae* CST0018, CC0301, USA011 and CCE0100. Fragmentation mass spectrum of the $[M+H]^+$ ion of m/z 1225.7 corresponding to syringomycin E (3-OH C12:0 syringomycin) produced by *P. syringae* CVB0031.

D) BGC organization and amino-acid sequences of the pseudomycin of *P. cichorii* CFBP4407 predicted by bioinformatics analysis. They were the same for *P. syringae* T3W0028. Fragmentation mass spectrum of the $[M+H]^+$ ion of m/z 1235.7 corresponding to pseudomycin C' (3-OH C16:0 pseudomycin) produced by *P. cichorii* CFBP4407.

E) BGC organization and amino-acid sequences of the syringomycin-2 of *P. syringae* USA0050 predicted by bioinformatics. They were the same for *P. syringae* USA0035. Fragmentation mass spectrum of the $[M+H]^+$ ion of m/z 1265.8 corresponding to 3-OH C16:1 syringomycin-2 produced by *P. syringae* USA0050.

F) BGC organization and amino-acid sequences of the pseudomycin-2 of *P. cichorii* 83.1. predicted by bioinformatics analysis and fragmentation mass spectrum of the $[M+H]^+$ ion of m/z 1263.7 corresponding to 3-OH C16:0 pseudomycin-2 produced by *P. cichorii* 83.1.

b ions are indicated with red circles and arrows, y ions are indicated with blue circles and arrows

A

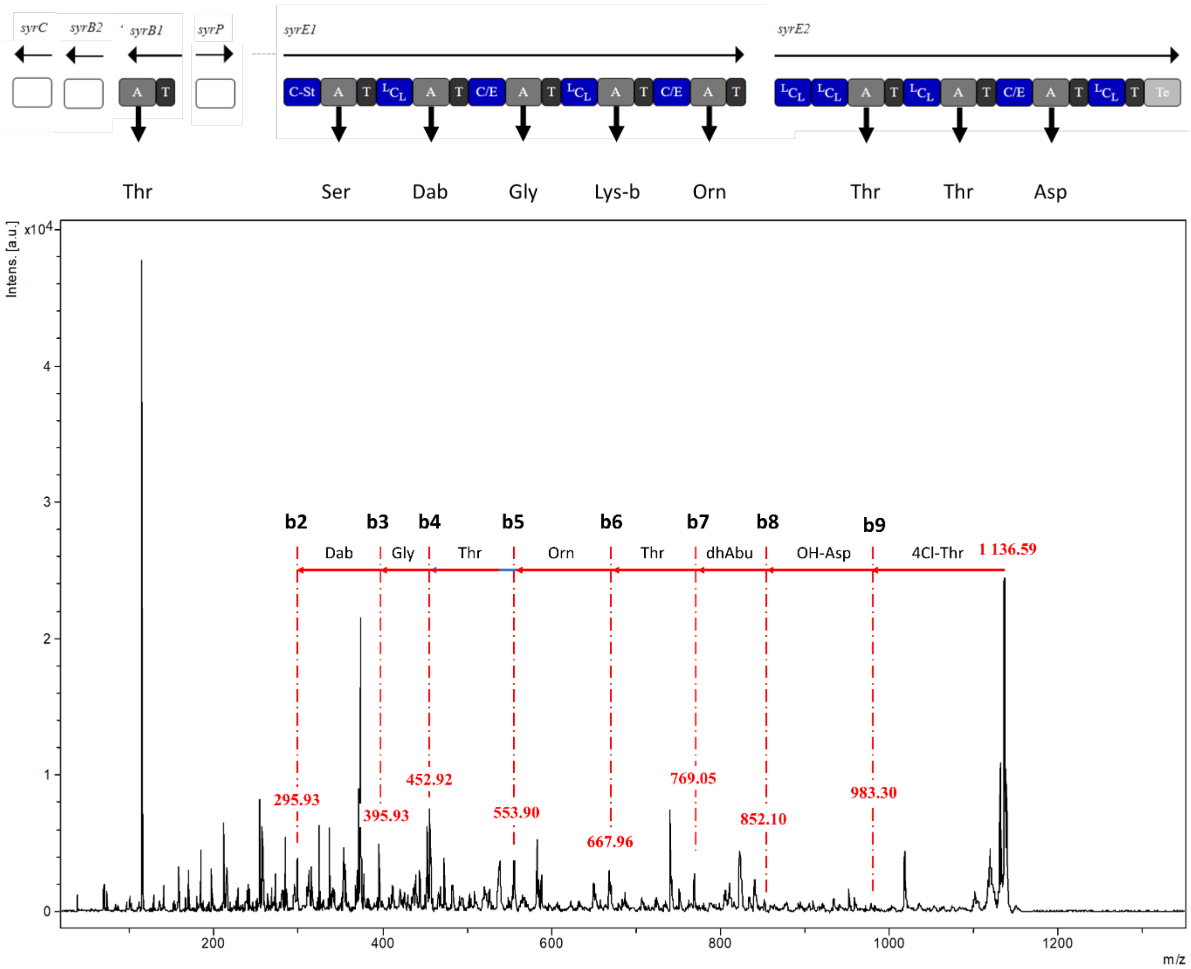

syrC
syrB2
syrB1
syrP
syrE1
syrE2
C-St
A
T
C/E
Te
Thr
Ser
Dab
Gly
Lys-b
Orn
Thr
Thr
Asp
Intens. [a.u.]
x10^4
b2
b3
b4
b5
b6
b7
b8
b9
Dab
Gly
Thr
Orn
Thr
dhAbu
OH-Asp
4Cl-Thr
1 136.59
295.93
395.93
452.92
553.90
667.96
769.05
852.10
983.30
m/z

B

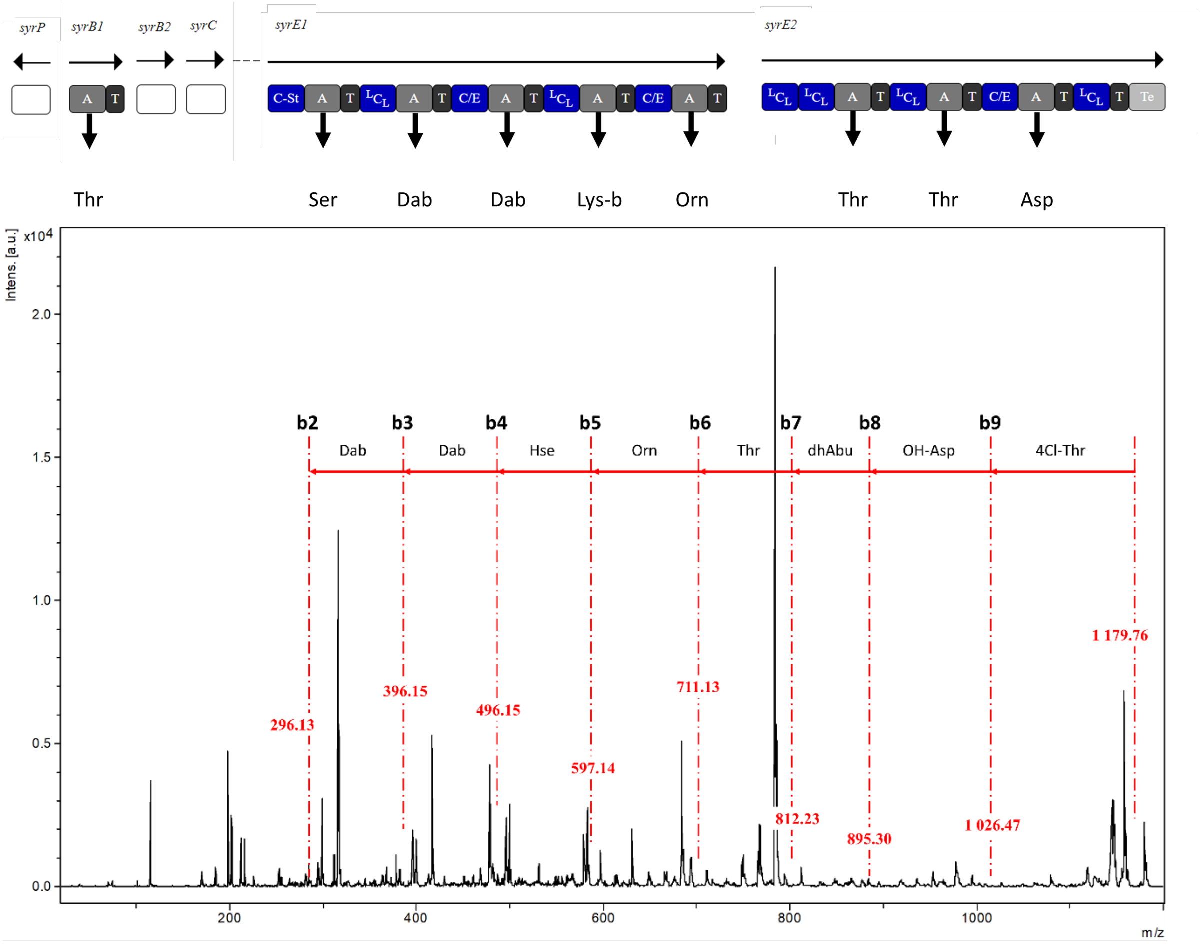
syrP
syrB1
syrB2
syrC
syrE1
syrE2
A T
C-St A T LCL A T C/E A T LCL A T C/E A T
LCL LCL A T LCL A T C/E A T LCL T Te
Thr
Ser
Dab
Dab
Lys-b
Orn
Thr
Thr
Asp
Intens. [a.u.]
x10^4
2.0
1.5
1.0
0.5
0.0
b2
b3
b4
b5
b6
b7
b8
b9
Dab
Dab
Hse
Orn
Thr
dhAbu
OH-Asp
4Cl-Thr
296.13
396.15
496.15
597.14
711.13
812.23
895.30
1 026.47
1 179.76
200
400
600
800
1000
m/z

C

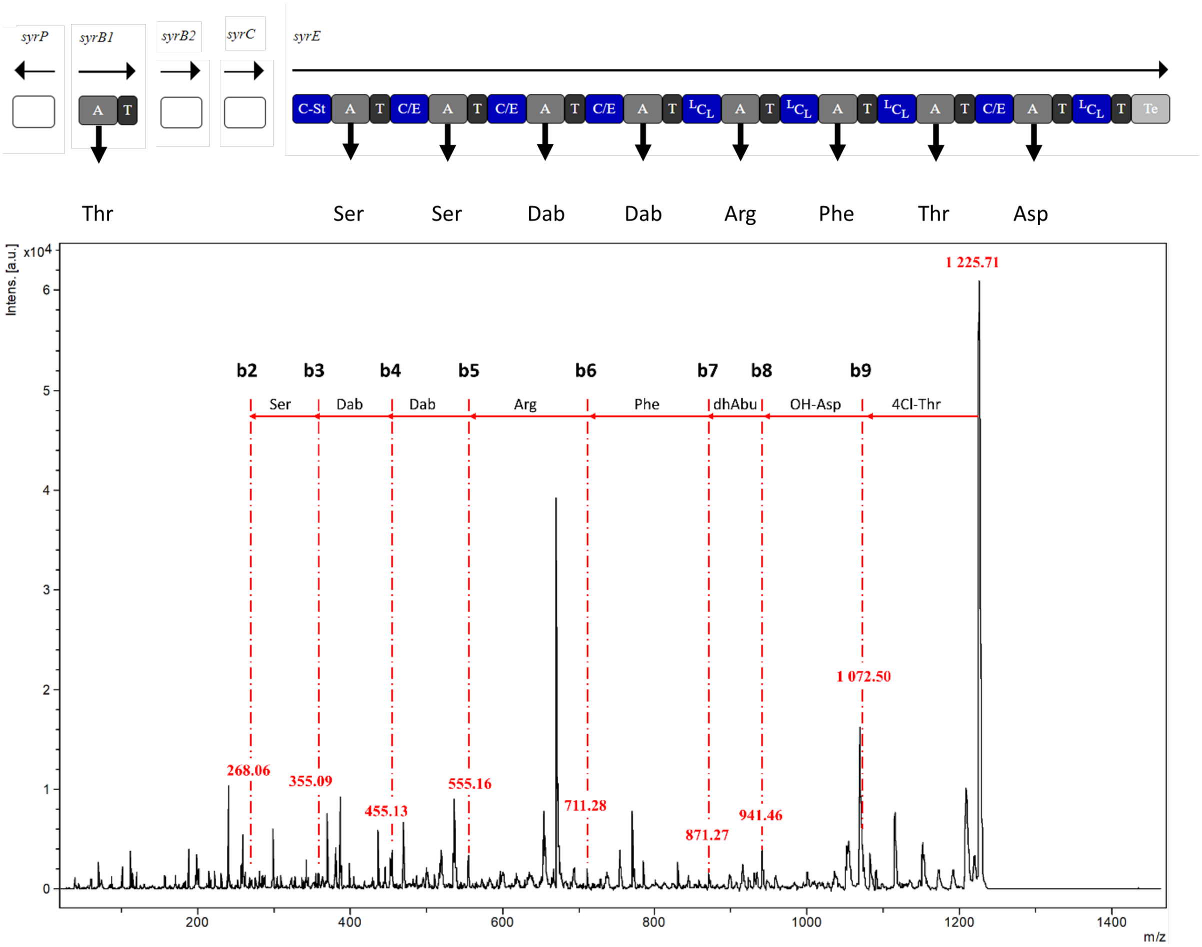

syrP
syrB1
syrB2
syrC
syrE
A T
C-St A T C/E A T C/E A T C/E A T LCL A T LCL A T LCL A T C/E A T LCL T Te
Thr
Ser
Ser
Dab
Dab
Arg
Phe
Thr
Asp
Intens. [a.u.]
x10^4
b2 b3 b4 b5 b6 b7 b8 b9
Ser Dab Dab Arg Phe dhAbu OH-Asp 4Cl-Thr
1 225.71
1 072.50
268.06
355.09
455.13
555.16
711.28
871.27
941.46
m/z

D

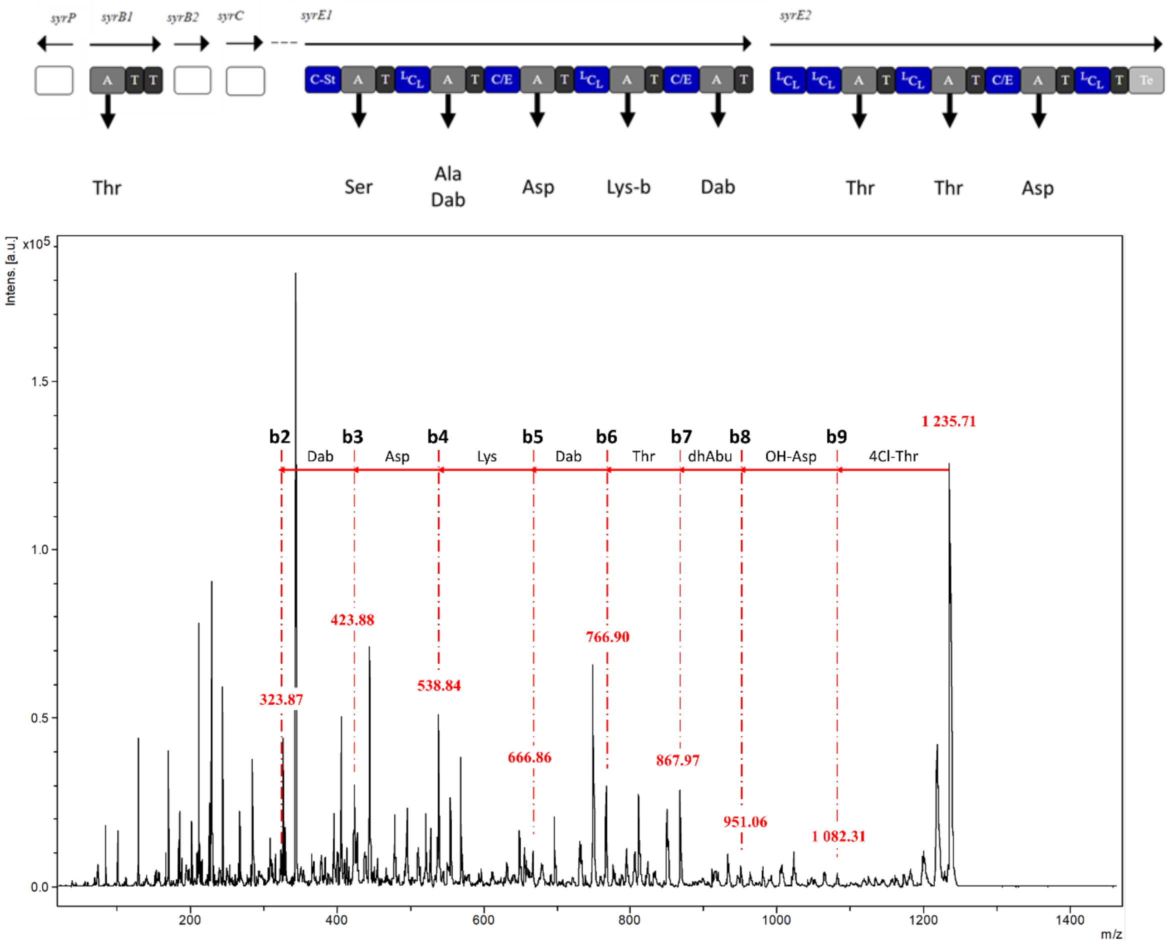

syrP
syrB1
syrB2
syrC
syrE1
syrE2
C-St
C/E
Te
Thr
Ser
Ala
Dab
Asp
Lys-b
Dab
Thr
Thr
Asp
Intens. [a.u.]
x10^5
b2
b3
b4
b5
b6
b7
b8
b9
Dab
Asp
Lys
Dab
Thr
dhAbu
OH-Asp
4Cl-Thr
1 235.71
323.87
423.88
538.84
666.86
766.90
867.97
951.06
1 082.31
m/z

E

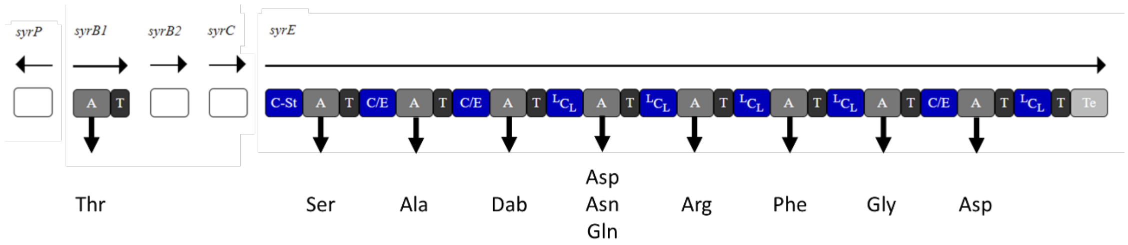

syrP
syrB1
syrB2
syrC
syrE
A
T
C-St
C/E
$^{L}C_{L}$
Te
Thr
Ser
Ala
Dab
Asp
Asn
Gln
Arg
Phe
Gly
Asp

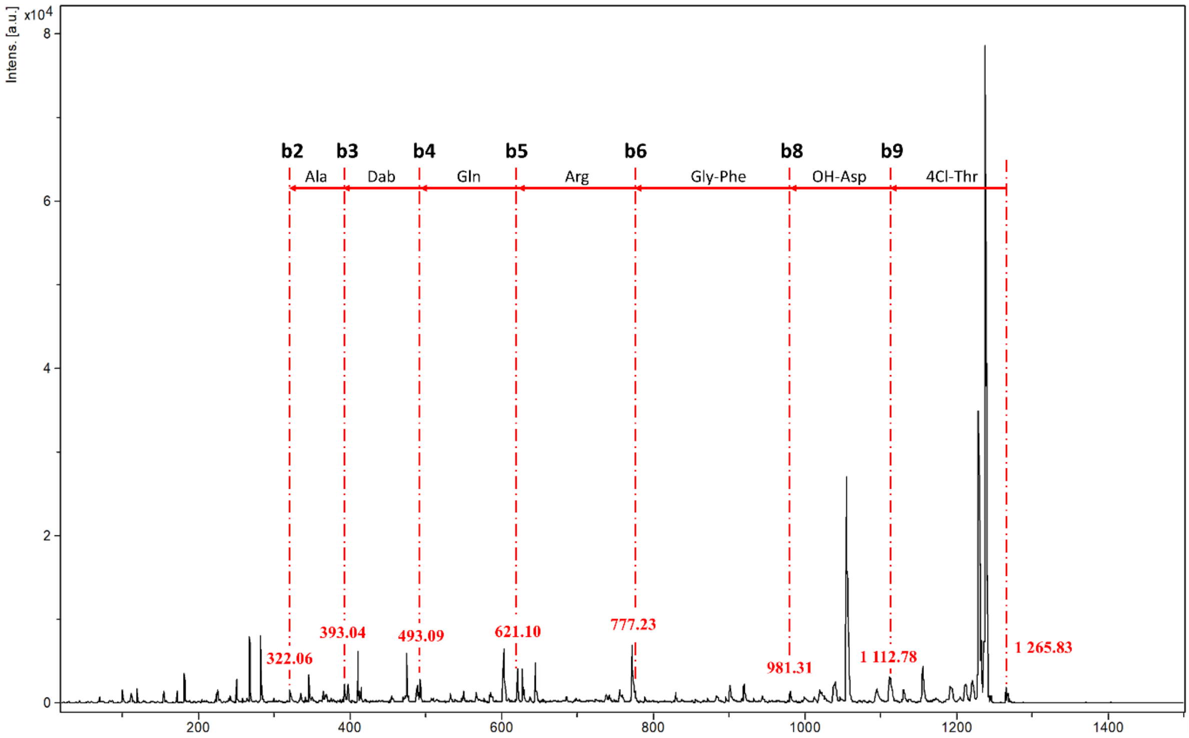

Intens. [a.u.]
x10^4
b2
b3
b4
b5
b6
b8
b9
Ala
Dab
Gln
Arg
Gly-Phe
OH-Asp
4Cl-Thr
322.06
393.04
493.09
621.10
777.23
981.31
1 112.78
1 265.83
200
400
600
800
1000
1200
1400
0
2
4
6
8

F

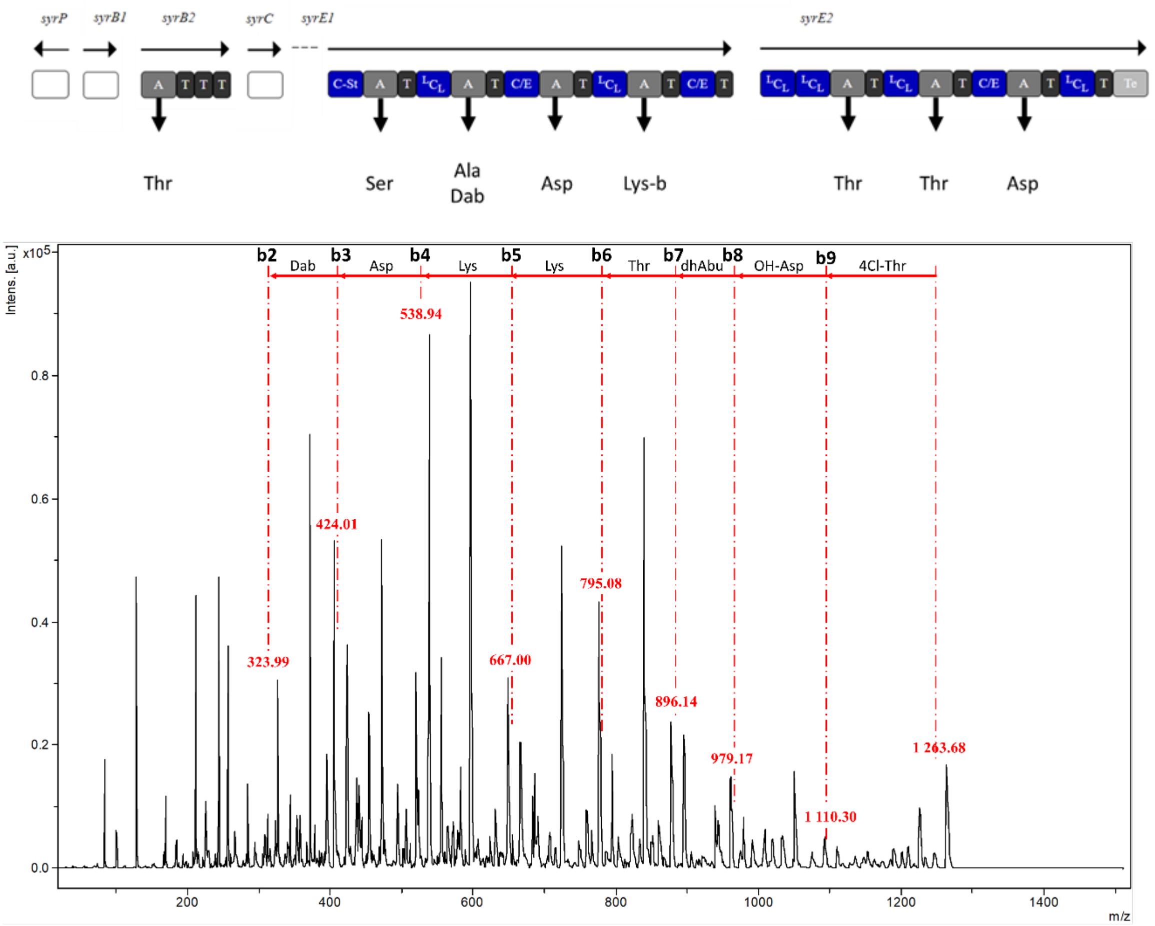
syrP
syrB1
syrB2
syrC
syrE1
syrE2
Thr
Ser
Ala
Dab
Asp
Lys-b
Thr
Thr
Asp
b2
b3
b4
b5
b6
b7
b8
b9
Dab
Asp
Lys
Lys
Thr
dhAbu
OH-Asp
4Cl-Thr
323.99
424.01
538.94
667.00
795.08
896.14
979.17
1 110.30
1 263.68
Intens. [a.u.]
x10^5
m/z

Supplementary Figure S4:
A) BGC organization and amino-acid sequences of the cichopeptin-2 of *P. syringae* GAW0197 predicted by bioinformatics analysis. They were the same for *P. syringae* CMO0085 and CST0099. Fragmentation mass spectrum of the $[M+H]^+$ ion of m/z 2011.2 corresponding to 3-OH C10:0 cichopeptin-2 produced by *P. syringae* GAW0197.
B) BGC organization and amino-acid sequences of the cichopeptin-3 of *P.cichorii* 83.1 predicted by bioinformatics analysis and fragmentation mass spectrum of the $[M+H]^+$ ion of m/z 2077.3 corresponding to 3-OH C12:1 cichopeptin-3 produced by *P.cichorii* 83.1.
C) BGC organization and amino-acid sequences of the cichorinotoxin of *P.cichorii* CFBP4407. predicted by bioinformatics analysis and fragmentation mass spectrum of the $[M+H]^+$ ion of m/z 2069.3 corresponding to cichorinotoxin (3-OH C10:0 cichorinotoxin) produced by *P.cichorii* CFBP4407.
D) BGC organization and amino-acid sequences of the syringopeptin 22 of *P.syringae* USA011 predicted by bioinformatics analysis. They were the same for *P. syringae* CC0301, USA0035, USA0050, CMO0085 and CCE0100. Fragmentation mass spectrum of the $[M+H]^+$ ion of m/z 2144.4 corresponding to syringopeptin 22A (3-OH C10:0 syringopeptin 22) produced by *P.syringae* USA011.
E) BGC organization and amino-acid sequences of syringopeptin 22-2 of *P. syringae* CCV0214 predicted by bioinformatics analysis. Fragmentation mass spectrum of the $[M+H]^+$ ion of m/z 2146.4 corresponding to 3-OH C10:0 syringopeptin 22-2 produced by *P. syringae* CCV0214.
F) BGC organization and amino-acid sequences of syringopeptin 22-3 of *P.syringae* CVB0031. predicted by bioinformatics analysis. Fragmentation mass spectrum of the $[M+H]^+$ ion of m/z 2160.2 corresponding to 3-OH C10:0 syringopeptin 22-3 produced by *P.syringae* CVB0031.
G) BGC organization and amino-acid sequences of syringopeptin 508 of *P. syringae* CST0077 predicted by bioinformatics analysis for *P.syringae* CST0018 followed by the Fragmentation mass spectrum of the $[M+H]^+$ ion of m/z 2188,2 corresponding to syringopeptin 508A (3-OH C12:0 syringopeptin 508) produced by *P. syringae* CST0077.

b ions are indicated with red circles and arrows, y ions are indicated with blue circles and arrows

A

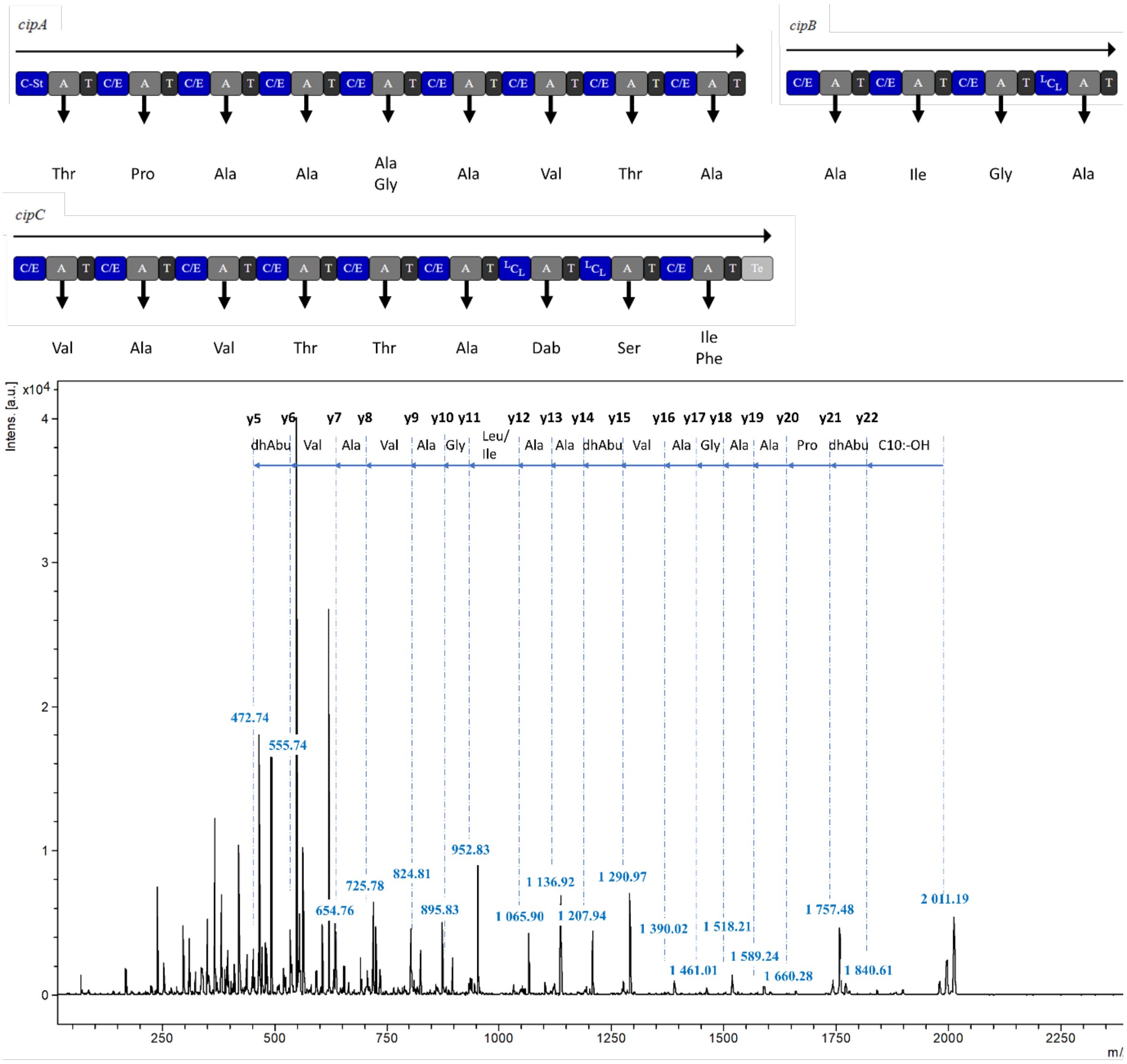

cipA
C-St A T C/E A T C/E A T C/E A T C/E A T C/E A T C/E A T C/E A T C/E A T
Thr Pro Ala Ala Ala Gly Ala Val Thr Ala
cipB
C/E A T C/E A T C/E A T LCL A T
Ala Ile Gly Ala
cipC
C/E A T C/E A T C/E A T C/E A T C/E A T C/E A T LCL A T LCL A T C/E A T Te
Val Ala Val Thr Thr Ala Dab Ser Ile Phe
Intens. [a.u.]
x10^4
y5 y6 y7 y8 y9 y10 y11 y12 y13 y14 y15 y16 y17 y18 y19 y20 y21 y22
dhAbu Val Ala Val Ala Gly Leu/Ile Ala Ala dhAbu Val Ala Gly Ala Ala Pro dhAbu C10:-OH
472.74
555.74
654.76
725.78
824.81
895.83
952.83
1 065.90
1 136.92
1 207.94
1 290.97
1 390.02
1 461.01
1 518.21
1 589.24
1 660.28
1 757.48
1 840.61
2 011.19
m/z

B

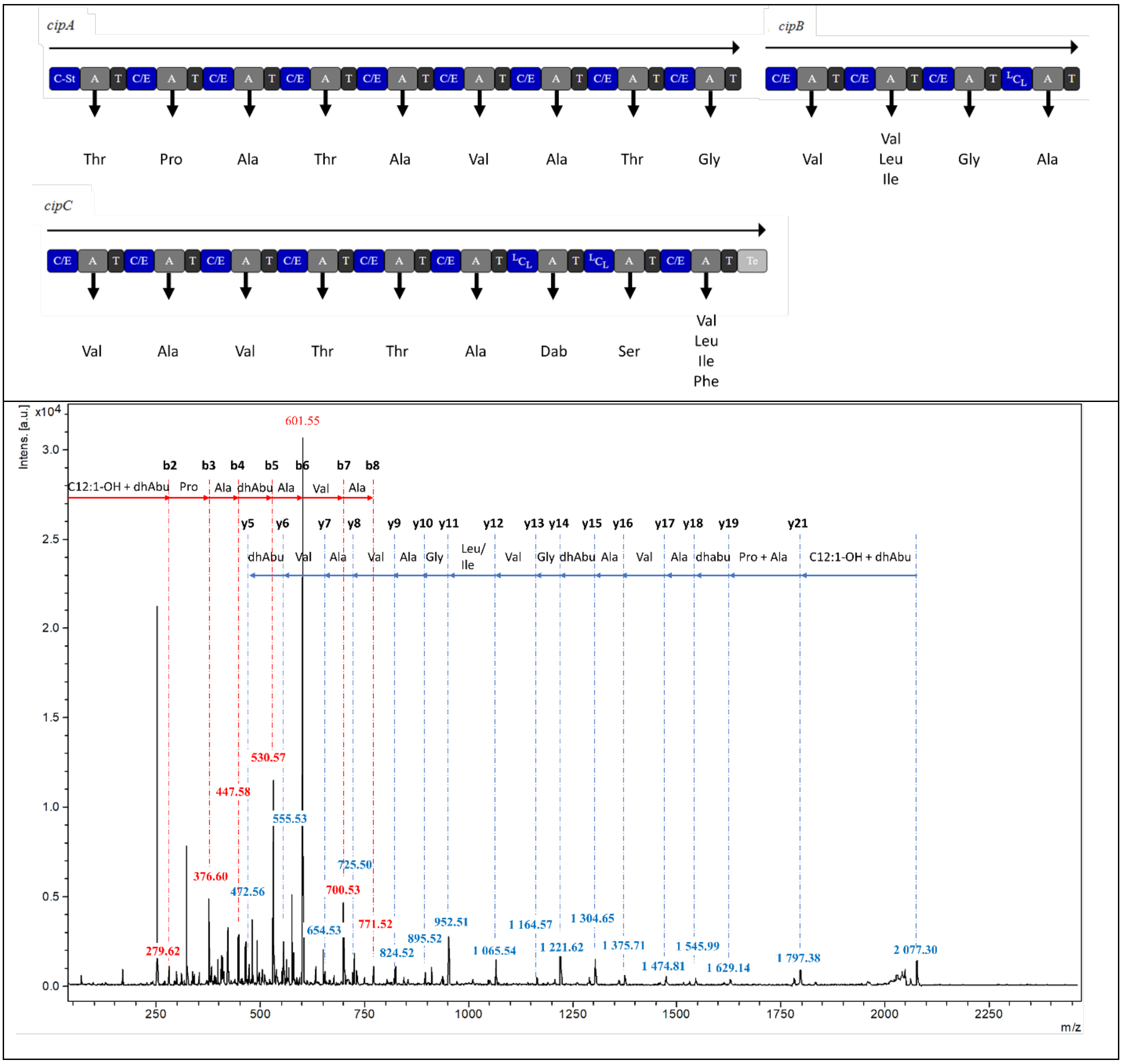
cipA
C-St A T C/E A T C/E A T C/E A T C/E A T C/E A T C/E A T C/E A T C/E A T
Thr Pro Ala Thr Ala Val Ala Thr Gly
cipB
C/E A T C/E A T C/E A T LCL A T
Val
Val Leu Ile
Gly
Ala
cipC
C/E A T C/E A T C/E A T C/E A T C/E A T C/E A T LCL A T LCL A T C/E A T Te
Val Ala Val Thr Thr Ala Dab Ser
Val Leu Ile Phe
x10^4
Intens. [a.u.]
601.55
b2 b3 b4 b5 b6 b7 b8
C12:1-OH + dhAbu Pro Ala dhAbu Ala Val Ala
y5 y6 y7 y8 y9 y10 y11 y12 y13 y14 y15 y16 y17 y18 y19 y21
dhAbu Val Ala Val Ala Gly Leu/ Ile Val Gly dhAbu Ala Val Ala dhabu Pro + Ala C12:1-OH + dhAbu
530.57
447.58
555.53
376.60
472.56
725.50
700.53
279.62
654.53
771.52
824.52
895.52
952.51
1 065.54
1 164.57
1 221.62
1 304.65
1 375.71
1 474.81
1 545.99
1 629.14
1 797.38
2 077.30
m/z

C

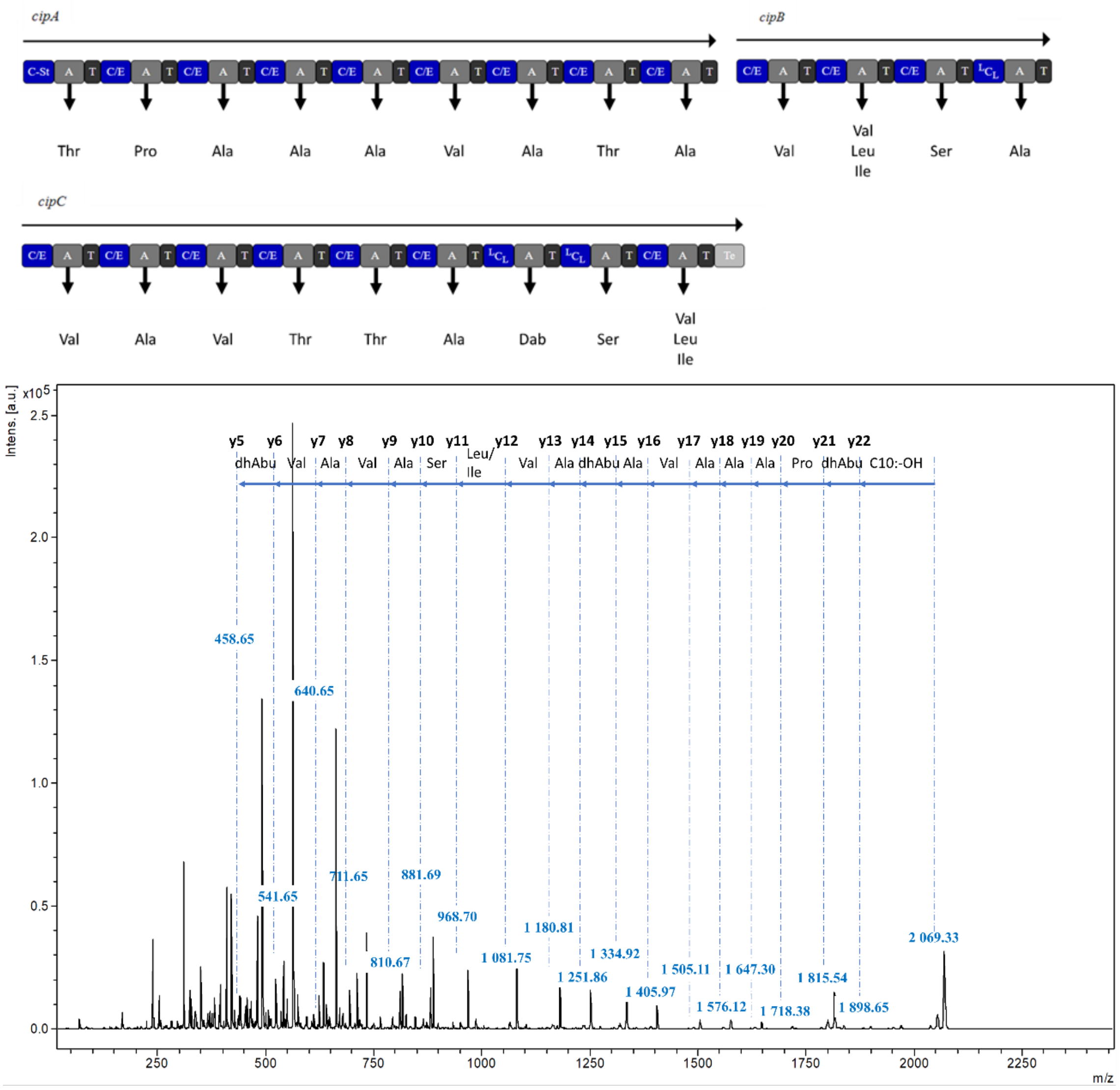
cipA
C-St A T C/E A T C/E A T C/E A T C/E A T C/E A T C/E A T C/E A T C/E A T
Thr Pro Ala Ala Ala Val Ala Thr Ala
cipB
C/E A T C/E A T C/E A T LCL A T
Val
Val Leu Ile
Ser
Ala
cipC
C/E A T C/E A T C/E A T C/E A T C/E A T C/E A T LCL A T LCL A T C/E A T Te
Val Ala Val Thr Thr Ala Dab Ser
Val Leu Ile
Intens. [a.u.]
x10^5
y5 y6 y7 y8 y9 y10 y11 y12 y13 y14 y15 y16 y17 y18 y19 y20 y21 y22
dhAbu Val Ala Val Ala Ser Leu/Ile Val Ala dhAbu Ala Val Ala Ala Ala Pro dhAbu C10:-OH
458.65
640.65
541.65
711.65
881.69
968.70
810.67
1 081.75
1 180.81
1 251.86
1 334.92
1 405.97
1 505.11
1 576.12
1 647.30
1 718.38
1 815.54
1 898.65
2 069.33
m/z

D

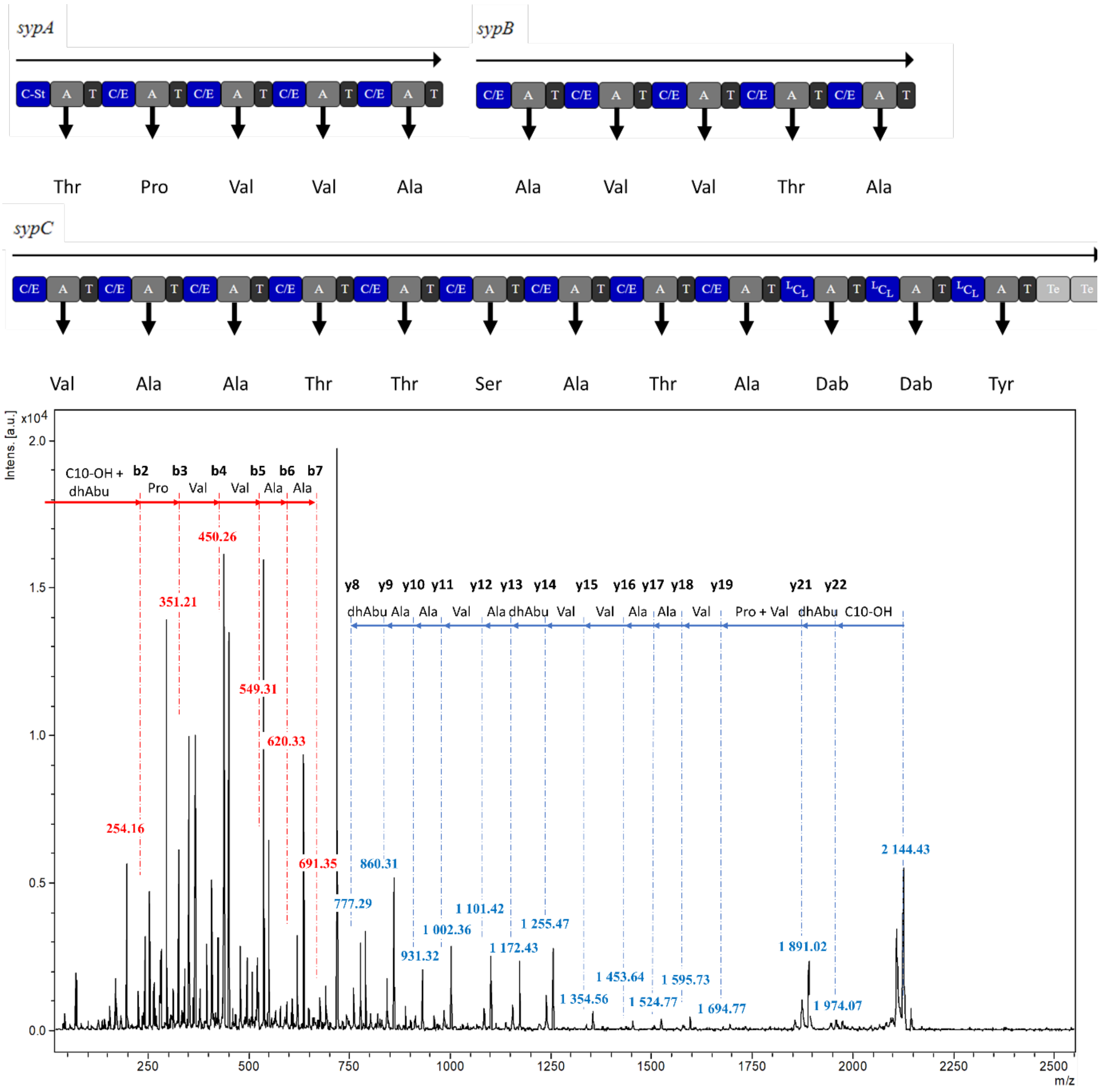

sypA
C-St A T C/E A T C/E A T C/E A T C/E A T
Thr Pro Val Val Ala
sypB
C/E A T C/E A T C/E A T C/E A T C/E A T
Ala Val Val Thr Ala
sypC
Val Ala Ala Thr Thr Ser Ala Thr Ala Dab Dab Tyr
Intens. [a.u.]
x10^4
C10-OH + dhAbu
b2 b3 b4 b5 b6 b7
Pro Val Val Ala Ala
254.16
351.21
450.26
549.31
620.33
691.35
y8 y9 y10 y11 y12 y13 y14 y15 y16 y17 y18 y19 y21 y22
dhAbu Ala Ala Val Ala dhAbu Val Val Ala Ala Val Pro + Val dhAbu C10-OH
777.29
860.31
931.32
1 002.36
1 101.42
1 172.43
1 255.47
1 354.56
1 453.64
1 524.77
1 595.73
1 694.77
1 891.02
1 974.07
2 144.43
m/z

E

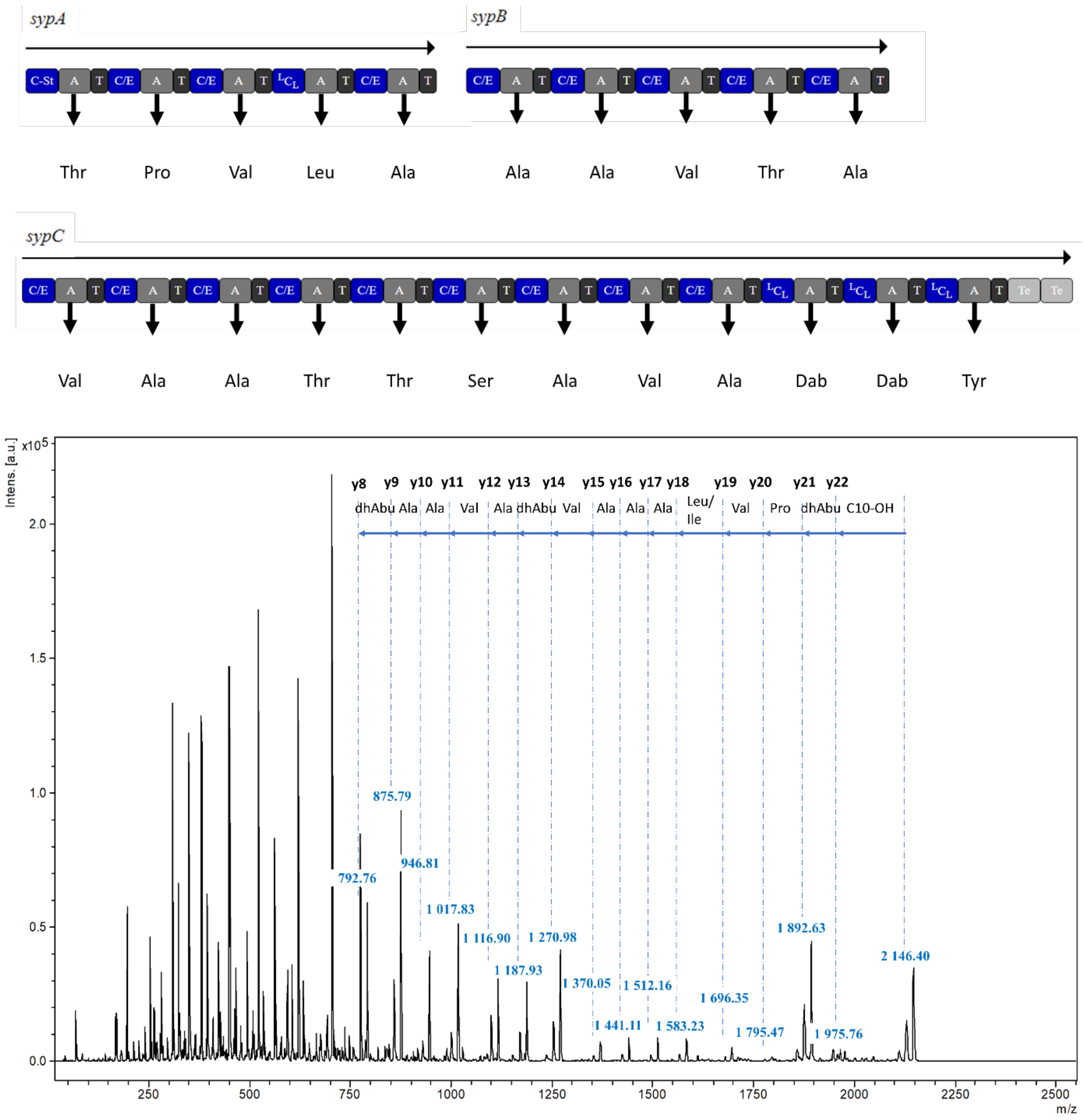

sypA
C-St A T C/E A T C/E A T LCL A T C/E A T
Thr Pro Val Leu Ala
sypB
C/E A T C/E A T C/E A T C/E A T C/E A T
Ala Ala Val Thr Ala
sypC
C/E A T C/E A T C/E A T C/E A T C/E A T C/E A T C/E A T C/E A T C/E A T LCL A T LCL A T LCL A T Te Te
Val Ala Ala Thr Thr Ser Ala Val Ala Dab Dab Tyr
y8 y9 y10 y11 y12 y13 y14 y15 y16 y17 y18 y19 y20 y21 y22
dhAbu Ala Ala Val Ala dhAbu Val Ala Ala Ala Leu/Ile Val Pro dhAbu C10-OH
792.76
875.79
946.81
1 017.83
1 116.90
1 187.93
1 270.98
1 370.05
1 441.11
1 512.16
1 583.23
1 696.35
1 795.47
1 892.63
1 975.76
2 146.40
Intens. [a.u.]
x10^5
m/z

F

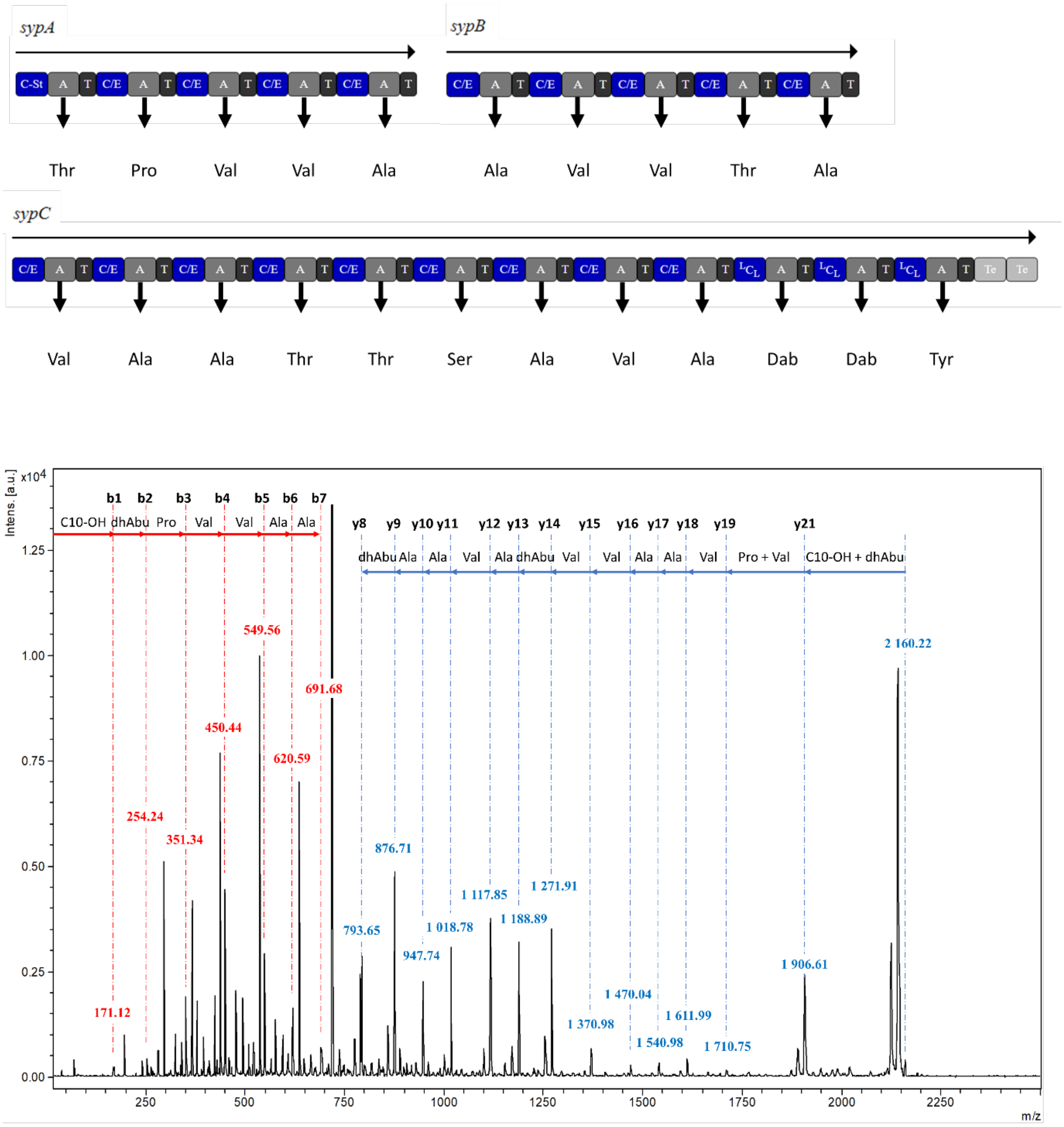

sypA
C-St A T C/E A T C/E A T C/E A T C/E A T
Thr Pro Val Val Ala
sypB
C/E A T C/E A T C/E A T C/E A T C/E A T
Ala Val Val Thr Ala
sypC
Val Ala Ala Thr Thr Ser Ala Val Ala Dab Dab Tyr
Intens. [a.u.]
x10^4
b1 b2 b3 b4 b5 b6 b7
C10-OH dhAbu Pro Val Val Ala Ala
y8 y9 y10 y11 y12 y13 y14 y15 y16 y17 y18 y19 y21
dhAbu Ala Ala Val Ala dhAbu Val Val Ala Ala Val Pro + Val C10-OH + dhAbu
171.12
254.24
351.34
450.44
549.56
620.59
691.68
793.65
876.71
947.74
1 018.78
1 117.85
1 188.89
1 271.91
1 370.98
1 470.04
1 540.98
1 611.99
1 710.75
1 906.61
2 160.22
m/z

G

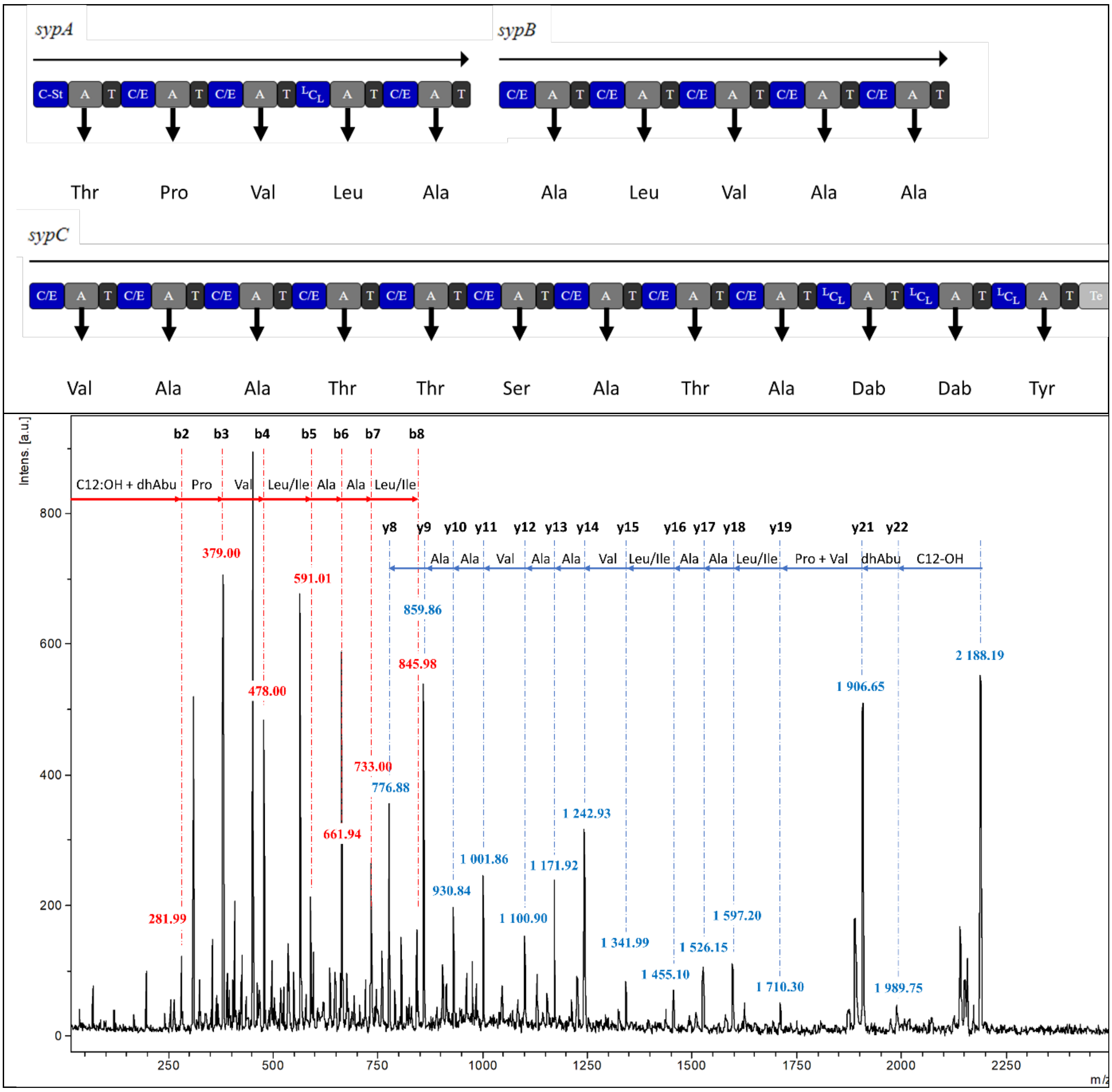

sypA
sypB
sypC
C-St A T C/E A T C/E A T LCL A T C/E A T
C/E A T C/E A T C/E A T C/E A T C/E A T
C/E A T C/E A T C/E A T C/E A T C/E A T C/E A T C/E A T C/E A T C/E A T LCL A T LCL A T LCL A T Te
Thr Pro Val Leu Ala Ala Leu Val Ala Ala
Val Ala Ala Thr Thr Ser Ala Thr Ala Dab Dab Tyr
Intens. [a.u.]
b2 b3 b4 b5 b6 b7 b8
C12:OH + dhAbu Pro Val Leu/Ile Ala Ala Leu/Ile
y8 y9 y10 y11 y12 y13 y14 y15 y16 y17 y18 y19 y21 y22
Ala Ala Val Ala Ala Val Leu/Ile Ala Ala Leu/Ile Pro + Val dhAbu C12-OH
281.99
379.00
478.00
591.01
661.94
733.00
845.98
776.88
859.86
930.84
1 001.86
1 100.90
1 171.92
1 242.93
1 341.99
1 455.10
1 526.15
1 597.20
1 710.30
1 906.65
1 989.75
2 188.19
0
200
400
600
800
250
500
750
1000
1250
1500
1750
2000
2250
m/z

Supplementary Figure S5:
A) Fragmentation mass spectrum of the $[M+H]^+$ ion of m/z 2399.5 corresponding to syringopeptin 25A (3-OH C10:0 syringopeptin 25 [Tyr25] produced by *P.syringae* CSZ0188.
B) BGC organization and amino-acid sequences of the syringopeptin 25-2 of *P. syringae* T3W0028 predicted by bioinformatics analysis and fragmentation mass spectrum of the $[M+H]^+$ ion of m/z 2399.9 corresponding to 3-OH C10:0 syringopeptin 25-2 produced by P. *syringae* T3W0028.
C) BGC organization and amino-acid sequences of the syringopeptin 25-3 of *P.syringae* UB0415 predicted by bioinformatics analysis. They were the same for *P. syringae* CFBP1392. Fragmentation mass spectrum of the $[M+H]^+$ ion of m/z 2413.8 corresponding to 3-OH C10:0 syringopeptin 25-3 produced by *P.syringae* UB0415.
D) BGC organization and amino-acid sequences of the syringopeptin 25-4 of *P. syringae* TA0005 predicted by bioinformatics analysis. They were the same for *P. syringae* TA0018. Fragmentation mass spectrum of the $[M+H]^+$ ion of m/z 2441.7 corresponding to 3-OH C10:0 syringopeptin 25-4 produced by *P. syringae* TA0005.
E) BGC organization and amino-acid sequences of the syringopeptin 25-5 of *P. syringae* USA0087 predicted by bioinformatics analysis and fragmentation mass spectrum of the $[M+H]^+$ ion of m/z 2443.6 corresponding to 3-OH C10:0 syringopeptin 25-5 produced by *P.syringae* USA0087.
F) BGC organization and amino-acid sequences of the syringopeptin 25-6 of *P. syringae* CVB0040 predicted by bioinformatics analysis. They were the same for *P. syringae* CVB0040 and CC1466. Fragmentation mass spectrum of the $[M+H]^+$ ion of m/z 2445.3 corresponding to 3-OH C10:0 syringopeptin 25-6 produced by *P.syringae* CC1466.

b ions are indicated with red circles and arrows, y ions are indicated with blue circles and arrows

A

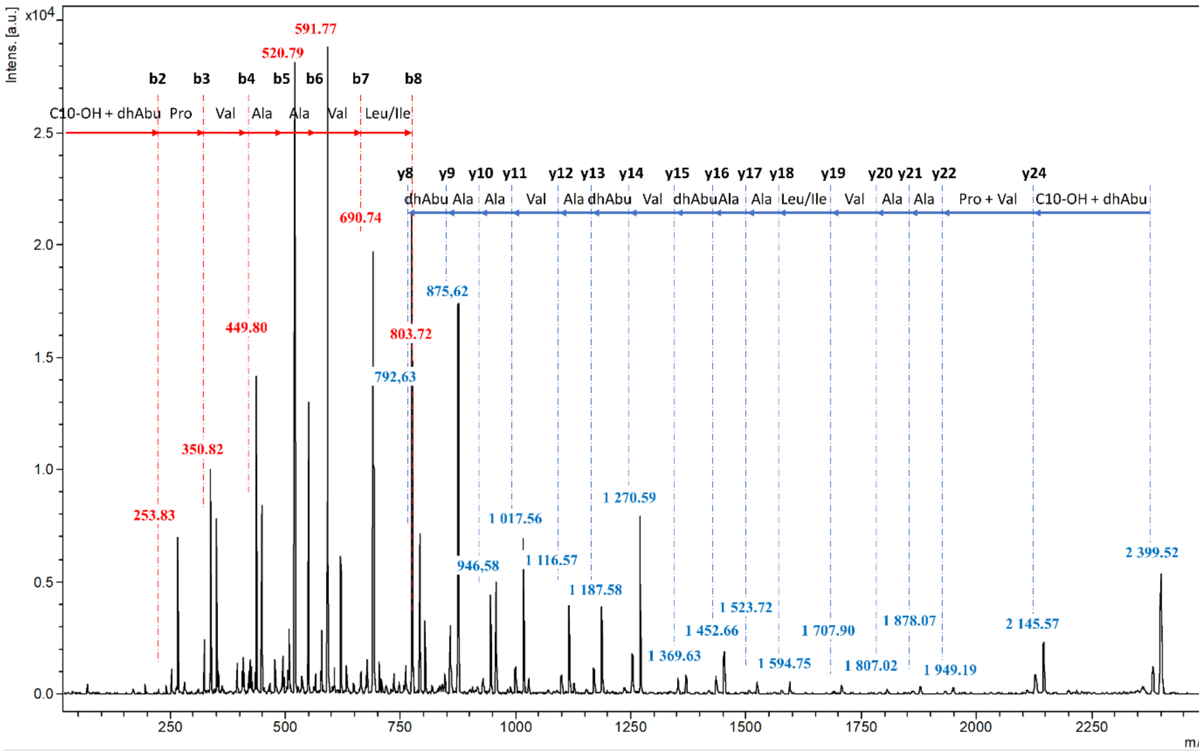

Intens. [a.u.]
x10^4
b2
b3
b4
b5
b6
b7
b8
C10-OH + dhAbu
Pro
Val
Ala
Ala
Val
Leu/Ile
253.83
350.82
449.80
520.79
591.77
690.74
803.72
y8
y9
y10
y11
y12
y13
y14
y15
y16
y17
y18
y19
y20
y21
y22
y24
dhAbu
Ala
Ala
Val
Ala
dhAbu
Val
dhAbu
Ala
Ala
Leu/Ile
Val
Ala
Ala
Pro + Val
C10-OH + dhAbu
792,63
875,62
946,58
1 017.56
1 116.57
1 187.58
1 270.59
1 369.63
1 452.66
1 523.72
1 594.75
1 707.90
1 807.02
1 878.07
1 949.19
2 145.57
2 399.52
m/

B

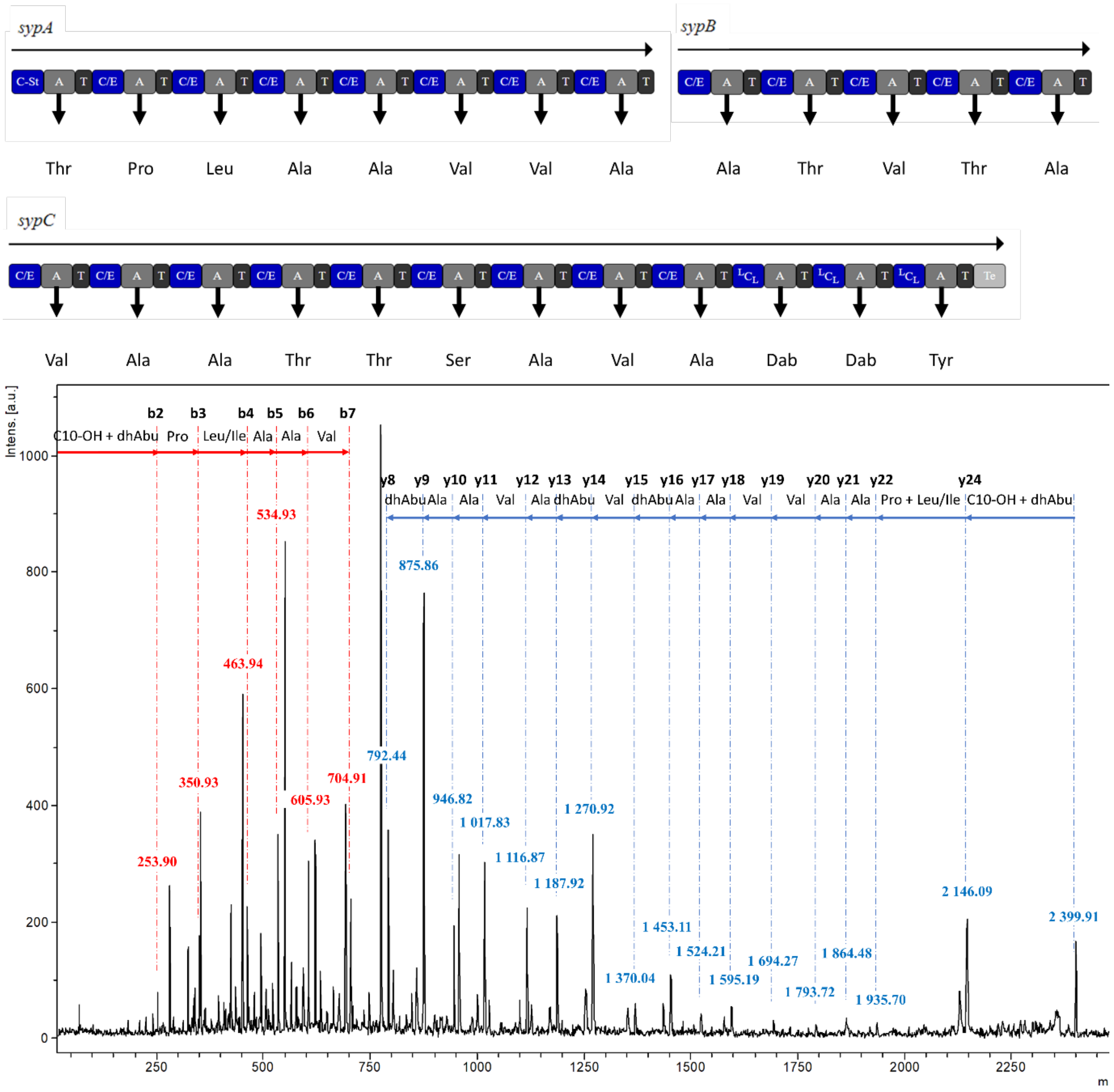
sypA
C-St A T C/E A T C/E A T C/E A T C/E A T C/E A T C/E A T C/E A T
Thr Pro Leu Ala Ala Val Val Ala
sypB
C/E A T C/E A T C/E A T C/E A T C/E A T
Ala Thr Val Thr Ala
sypC
C/E A T C/E A T C/E A T C/E A T C/E A T C/E A T C/E A T C/E A T C/E A T LCL A T LCL A T LCL A T Te
Val Ala Ala Thr Thr Ser Ala Val Ala Dab Dab Tyr
Intens. [a.u.]
b2 b3 b4 b5 b6 b7
C10-OH + dhAbu Pro Leu/Ile Ala Ala Val
y8 y9 y10 y11 y12 y13 y14 y15 y16 y17 y18 y19 y20 y21 y22 y24
dhAbu Ala Ala Val Ala dhAbu Val dhAbu Ala Ala Val Val Ala Ala Pro + Leu/Ile C10-OH + dhAbu
253.90
350.93
463.94
534.93
605.93
704.91
792.44
875.86
946.82
1 017.83
1 116.87
1 187.92
1 270.92
1 370.04
1 453.11
1 524.21
1 595.19
1 694.27
1 793.72
1 864.48
1 935.70
2 146.09
2 399.91
0
200
400
600
800
1000
250
500
750
1000
1250
1500
1750
2000
2250
m

C

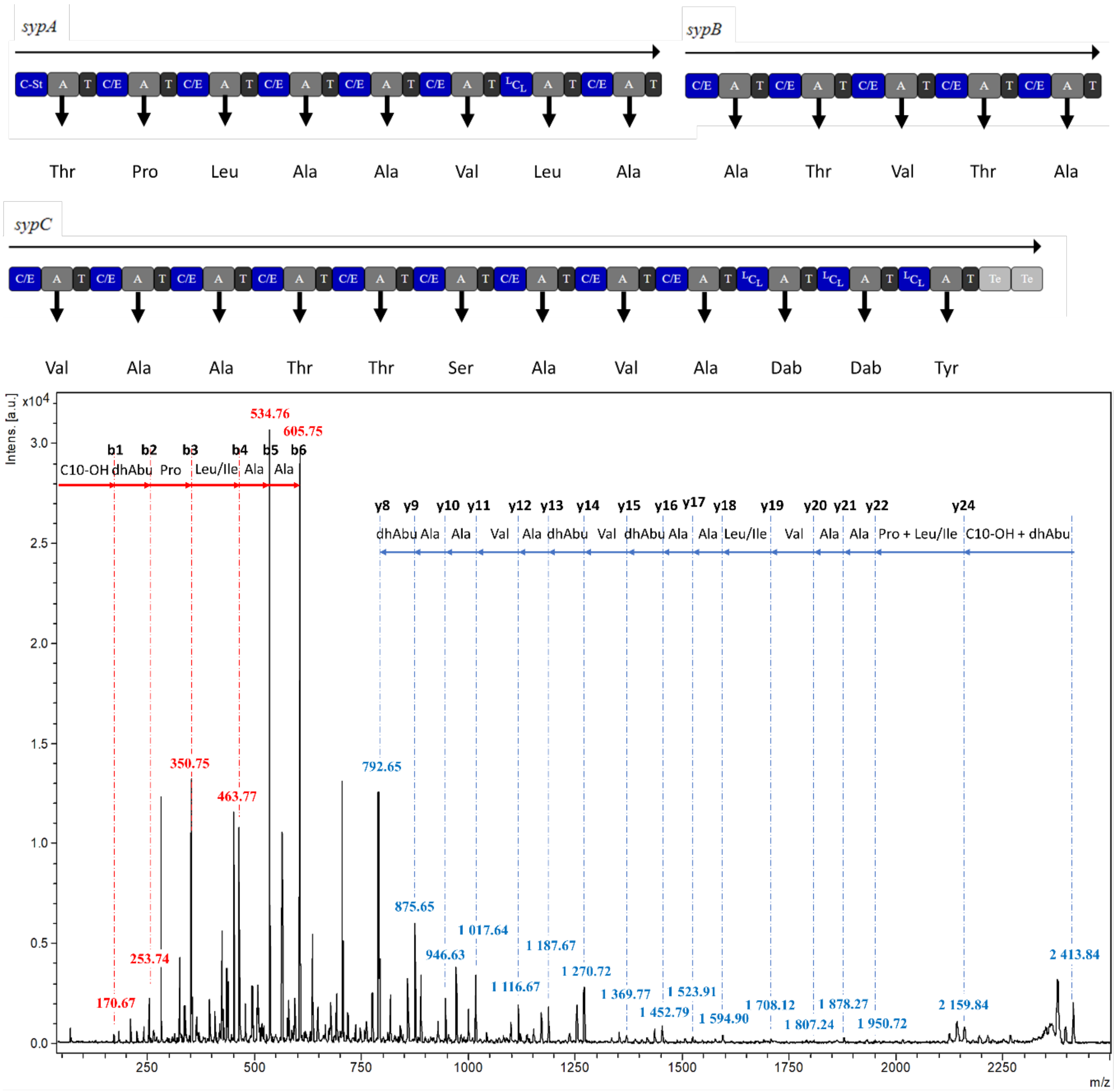

sypA
C-St A T C/E A T C/E A T C/E A T C/E A T C/E A T LCL A T C/E A T
Thr Pro Leu Ala Ala Val Leu Ala
sypB
C/E A T C/E A T C/E A T C/E A T C/E A T
Ala Thr Val Thr Ala
sypC
Val Ala Ala Thr Thr Ser Ala Val Ala Dab Dab Tyr
Te Te
Intens. [a.u.]
x10^4
b1 b2 b3 b4 b5 b6
C10-OH dhAbu Pro Leu/Ile Ala Ala
y8 y9 y10 y11 y12 y13 y14 y15 y16 y17 y18 y19 y20 y21 y22 y24
dhAbu Ala Ala Val Ala dhAbu Val dhAbu Ala Ala Leu/Ile Val Ala Ala Pro + Leu/Ile C10-OH + dhAbu
534.76
605.75
350.75
463.77
253.74
170.67
792.65
875.65
946.63
1 017.64
1 116.67
1 187.67
1 270.72
1 369.77
1 452.79
1 523.91
1 594.90
1 708.12
1 807.24
1 878.27
1 950.72
2 159.84
2 413.84
m/z

D

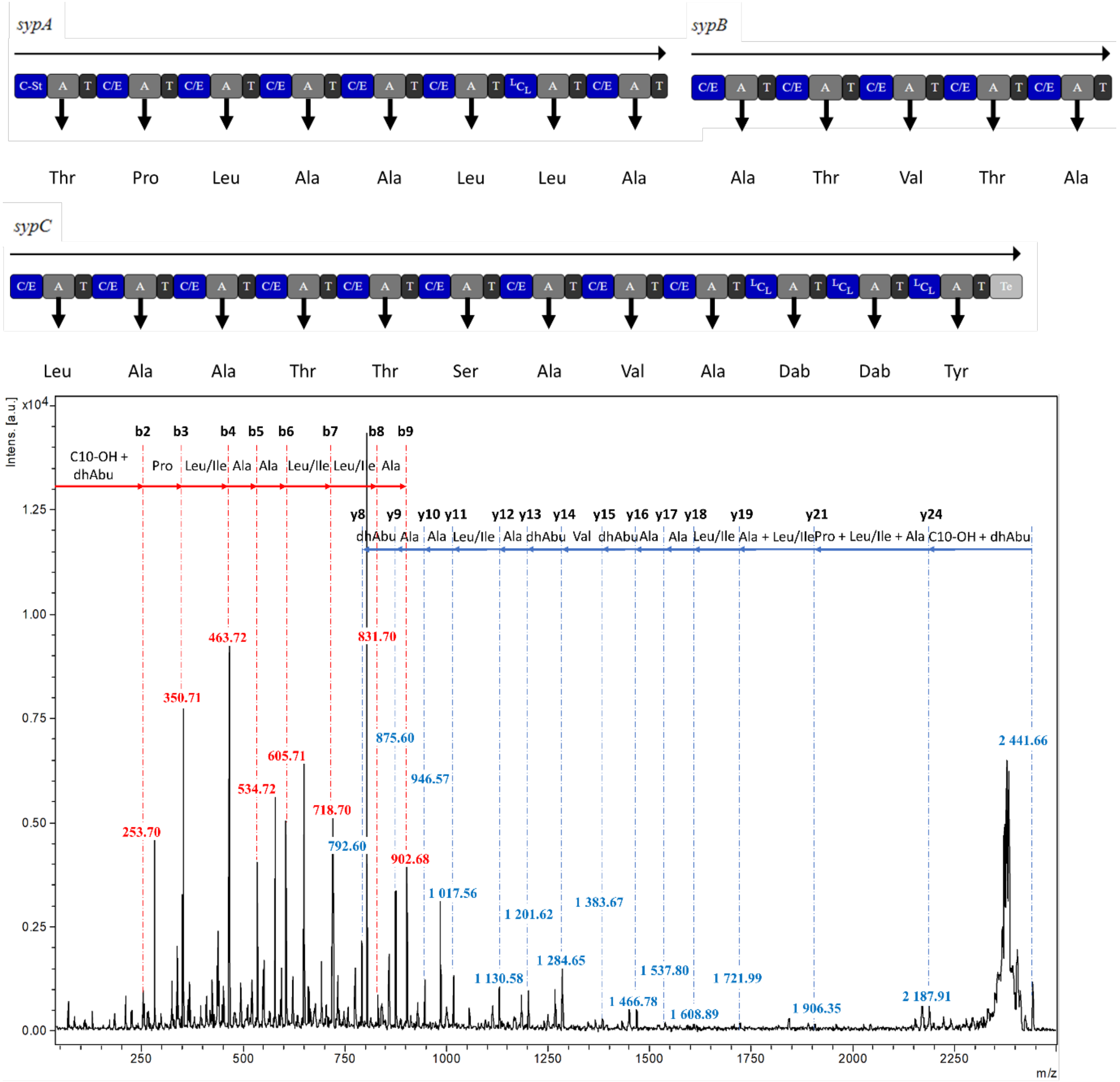
sypA
sypB
sypC
Thr
Pro
Leu
Ala
Ala
Leu
Leu
Ala
Ala
Thr
Val
Thr
Ala
Leu
Ala
Ala
Thr
Thr
Ser
Ala
Val
Ala
Dab
Dab
Tyr
Intens. [a.u.]
x10^4
C10-OH + dhAbu
b2 b3 b4 b5 b6 b7 b8 b9
Pro Leu/Ile Ala Ala Leu/Ile Leu/Ile Ala
y8 y9 y10 y11 y12 y13 y14 y15 y16 y17 y18 y19 y21 y24
dhAbu Ala Ala Leu/Ile Ala dhAbu Val dhAbu Ala Ala Leu/Ile Ala + Leu/Ile Pro + Leu/Ile + Ala C10-OH + dhAbu
253.70
350.71
463.72
534.72
605.71
718.70
831.70
902.68
792.60
875.60
946.57
1 017.56
1 130.58
1 201.62
1 284.65
1 383.67
1 466.78
1 537.80
1 608.89
1 721.99
1 906.35
2 187.91
2 441.66
m/z

E

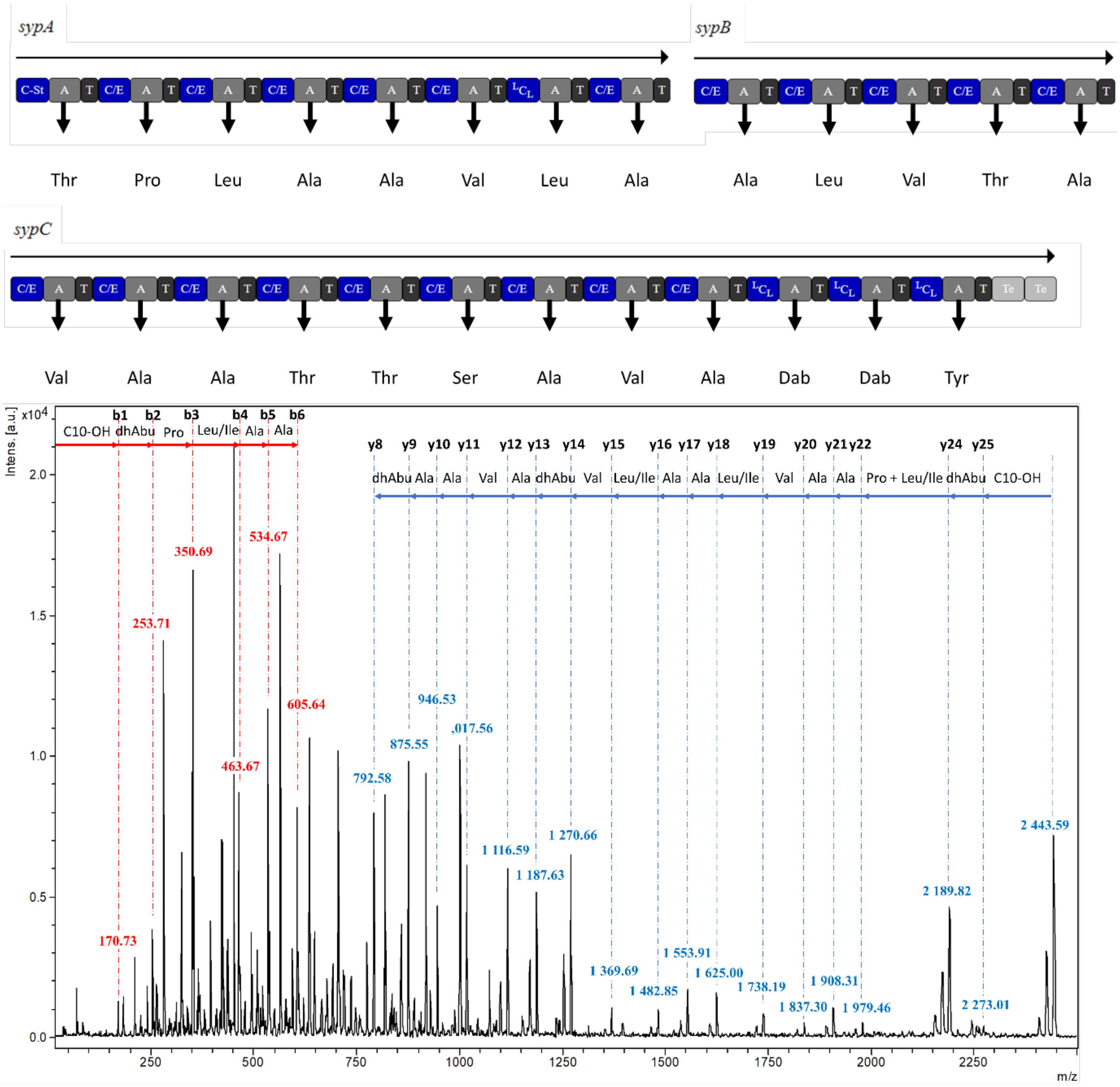

sypA
C-St A T C/E A T C/E A T C/E A T C/E A T C/E A T ᴸCL A T C/E A T
Thr Pro Leu Ala Ala Val Leu Ala
sypB
C/E A T C/E A T C/E A T C/E A T C/E A T
Ala Leu Val Thr Ala
sypC
C/E A T C/E A T C/E A T C/E A T C/E A T C/E A T C/E A T C/E A T C/E A T ᴸCL A T ᴸCL A T ᴸCL A T Te Te
Val Ala Ala Thr Thr Ser Ala Val Ala Dab Dab Tyr
x10^4
Intens. [a.u.]
b1 b2 b3 b4 b5 b6
C10-OH dhAbu Pro Leu/Ile Ala Ala
y8 y9 y10 y11 y12 y13 y14 y15 y16 y17 y18 y19 y20 y21 y22 y24 y25
dhAbu Ala Ala Val Ala dhAbu Val Leu/Ile Ala Ala Leu/Ile Val Ala Ala Pro + Leu/Ile dhAbu C10-OH
170.73
253.71
350.69
463.67
534.67
605.64
792.58
875.55
946.53
1 017.56
1 116.59
1 187.63
1 270.66
1 369.69
1 482.85
1 553.91
1 625.00
1 738.19
1 837.30
1 908.31
1 979.46
2 189.82
2 273.01
2 443.59
0.0 0.5 1.0 1.5 2.0
250 500 750 1000 1250 1500 1750 2000 2250
m/z

F

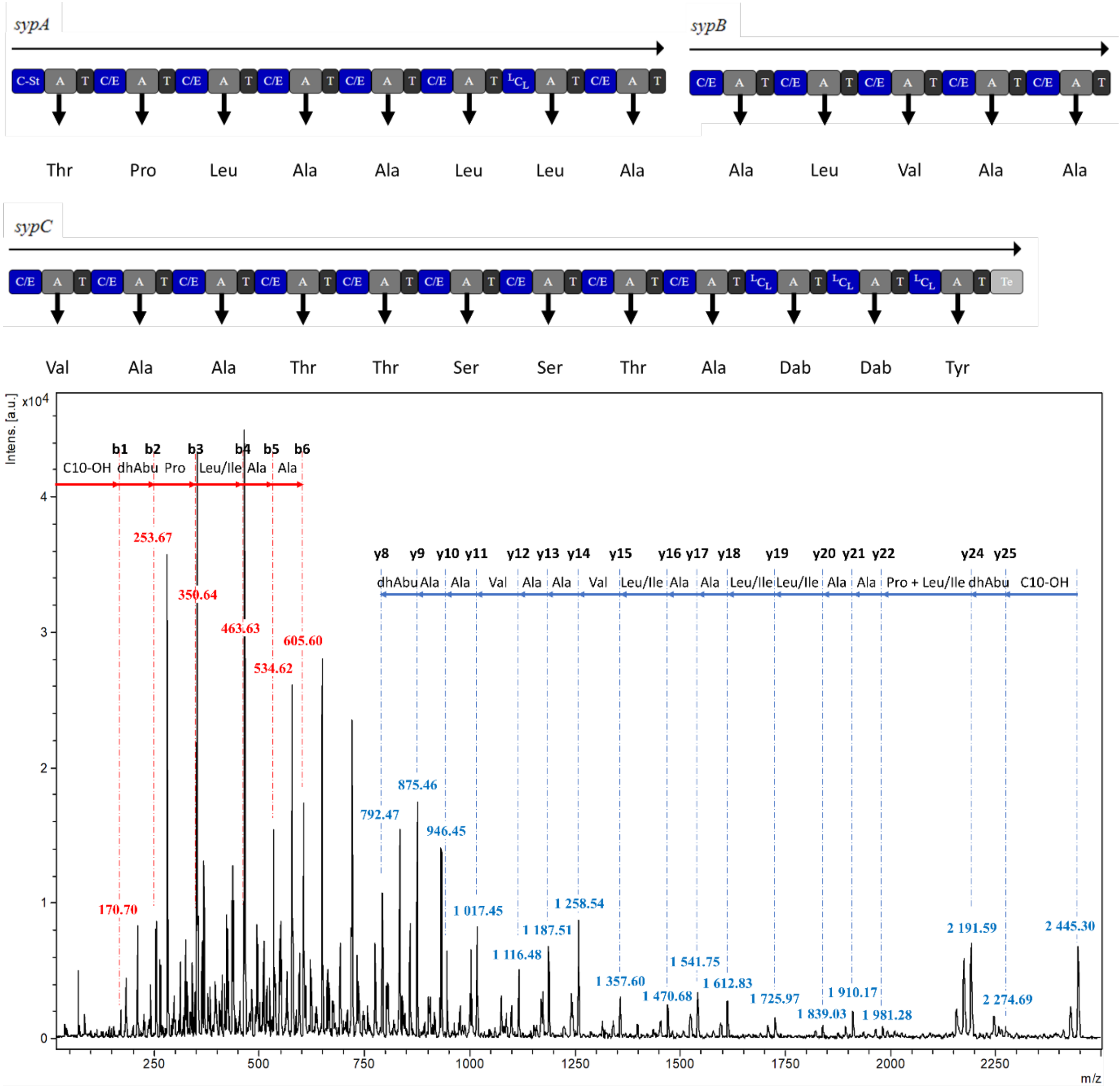
sypA
C-St A T C/E A T C/E A T C/E A T C/E A T C/E A T C/E A T $^{L}C_{L}$ A T C/E A T
Thr Pro Leu Ala Ala Leu Leu Ala
sypB
C/E A T C/E A T C/E A T C/E A T C/E A T
Ala Leu Val Ala Ala
sypC
C/E A T C/E A T C/E A T C/E A T C/E A T C/E A T C/E A T C/E A T C/E A T C/E A T $^{L}C_{L}$ A T $^{L}C_{L}$ A T $^{L}C_{L}$ A T Te
Val Ala Ala Thr Thr Ser Ser Thr Ala Dab Dab Tyr
Intens. [a.u.]
x10^{4}
b1 b2 b3 b4 b5 b6
C10-OH dhAbu Pro Leu/Ile Ala Ala
253.67
350.64
463.63
605.60
534.62
170.70
y8 y9 y10 y11 y12 y13 y14 y15 y16 y17 y18 y19 y20 y21 y22 y24 y25
dhAbu Ala Ala Val Ala Ala Val Leu/Ile Ala Ala Leu/Ile Leu/Ile Ala Ala Pro + Leu/Ile dhAbu C10-OH
875.46
792.47
946.45
1 017.45
1 258.54
1 187.51
1 116.48
1 357.60
1 541.75
1 470.68
1 612.83
1 725.97
1 910.17
1 839.03
1 981.28
2 191.59
2 445.30
2 274.69
0 1 2 3 4
250 500 750 1000 1250 1500 1750 2000 2250
m/z

Supplementary Figure S6: Putative chemical structures of factins (A), mycins (B) and peptins (C) characterized in this study

Supplementary Figure S6A

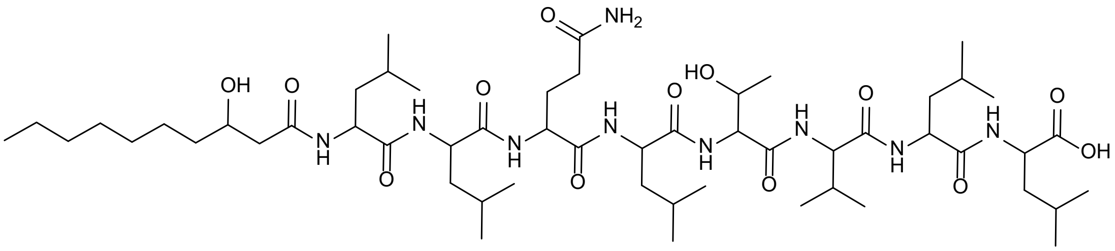

Syringafactin A = 3-OH C10:0 Syringafactin [Val6]
Molecular formula: $C_{54}H_{99}N_9O_{13}$
Exact mass: 1 081.74 Da

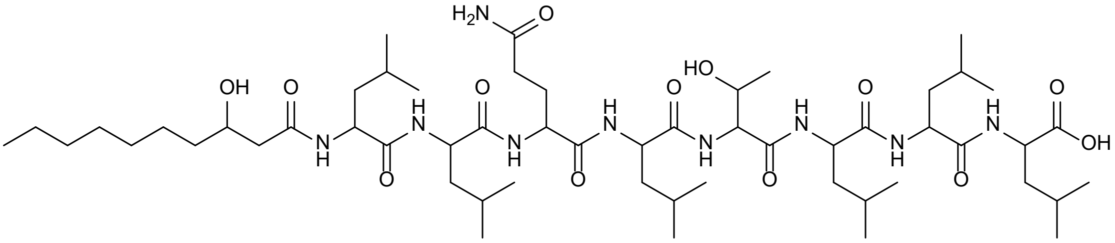

Syringafactin B = 3-OH C10:0 Syringafactin [Leu6]
Molecular formula: $C_{55}H_{101}N_9O_{13}$
Exact mass: 1 095.75 Da

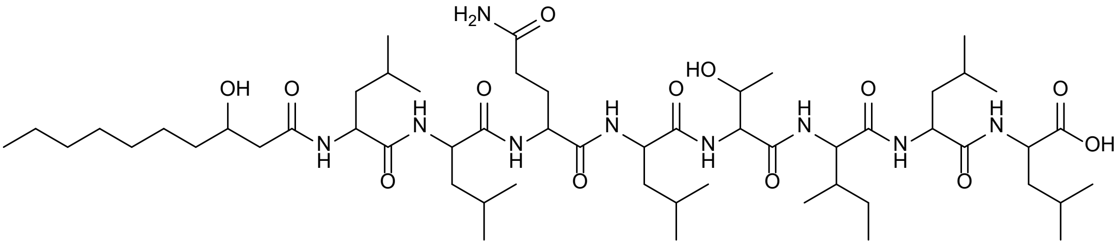

Syringafactin C = 3-OH C10:0 Syringafactin [Ile6]
Molecular formula: $C_{55}H_{101}N_9O_{13}$
Exact mass: 1 095.75 Da

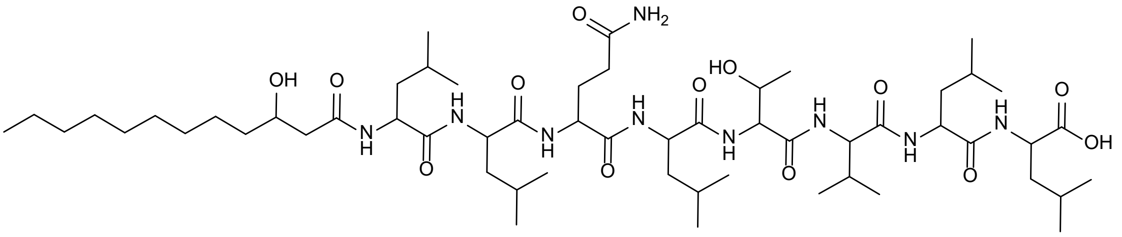

Syringafactin D = 3-OH C12:0 Syringafactin [Val6]
Molecular formula: $C_{56}H_{103}N_9O_{13}$
Exact mass: 1 109.77 Da

Supplementary Figure S6A continued.

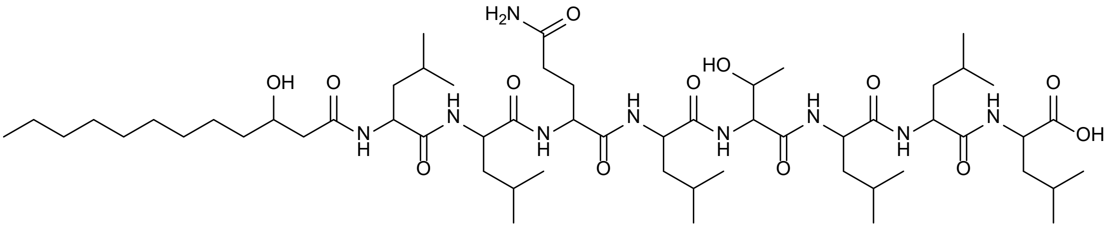

Syringafactin E = 3-OH C12:0 Syringafactin [Leu6]
Molecular formula: $C_{57}H_{105}N_9O_{13}$
Exact mass: 1 123.78 Da

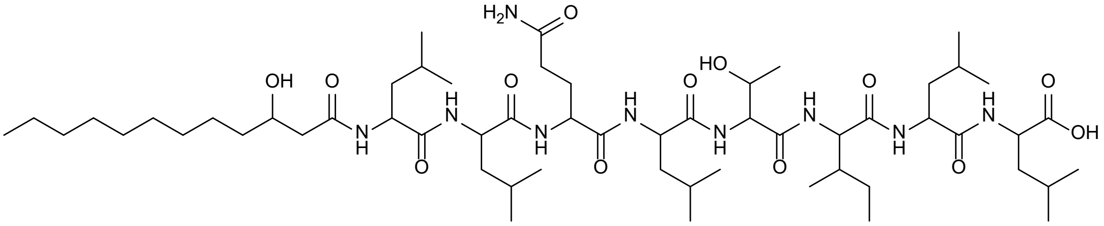

Syringafactin F = 3-OH C12:0 Syringafactin [Ile6]
Molecular formula: $C_{57}H_{105}N_9O_{13}$
Exact mass: 1 123.78 Da

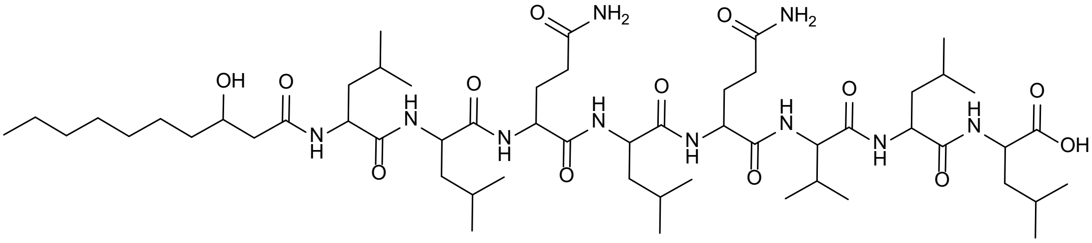

Cichofactin A = 3-OH C10:0 Cichofactin [Val6]
Molecular formula: $C_{55}H_{100}N_{10}O_{13}$
Exact mass: 1 108.75 Da

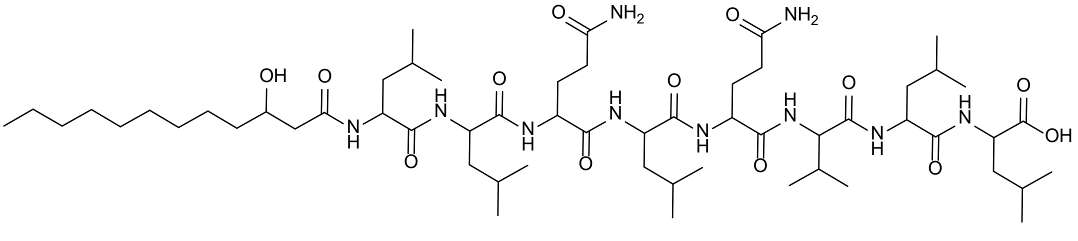

Cichofactin B = 3-OH C12:0 Cichofactin [Val6]
Molecular formula: $C_{57}H_{104}N_{10}O_{13}$
Exact mass: 1 136.78 Da

Supplementary Figure S6A continued.

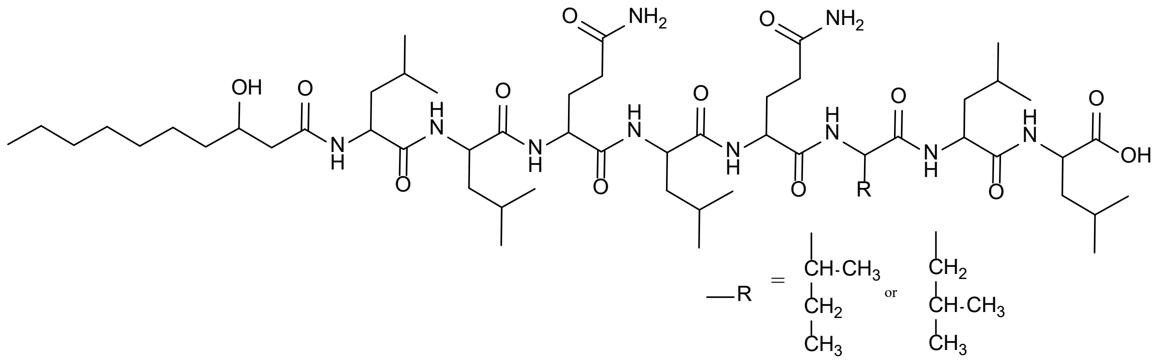

3-OH C10:0 Cichofactin [Leu6] or 3-OH C10:0 Cichofactin [Ile6]

Molecular formula: $C_{56}H_{102}N_{10}O_{13}$

Exact mass: 1 122.76 Da

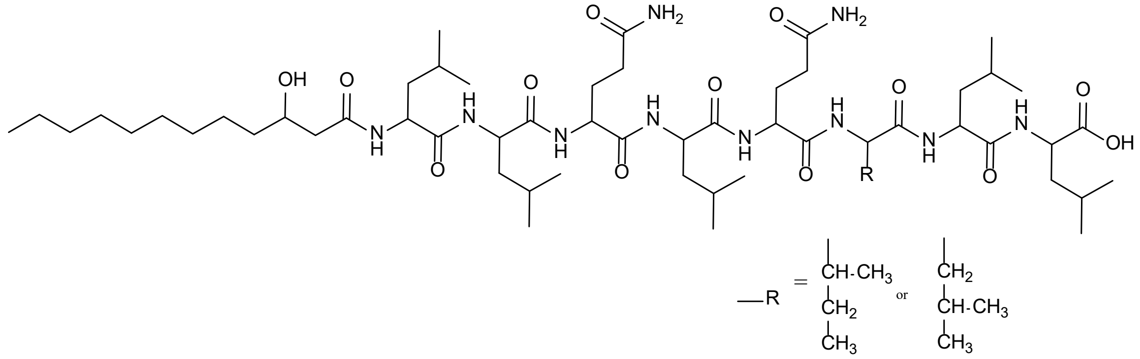

3-OH C12:0 Cichofactin [Leu6] or 3-OH C12:0 [Ile6]

Molecular formula: $C_{58}H_{106}N_{10}O_{13}$

Exact mass: 1 150.79 Da

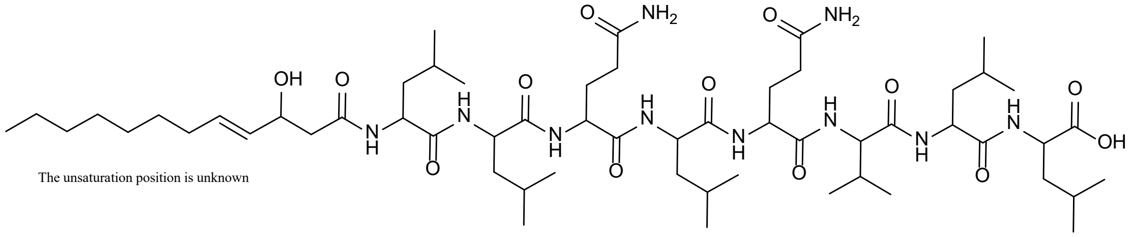

3-OH C12:1 Cichofactin [Val6]

Molecular formula: $C_{57}H_{102}N_{10}O_{13}$

Exact mass: 1 134.76 Da

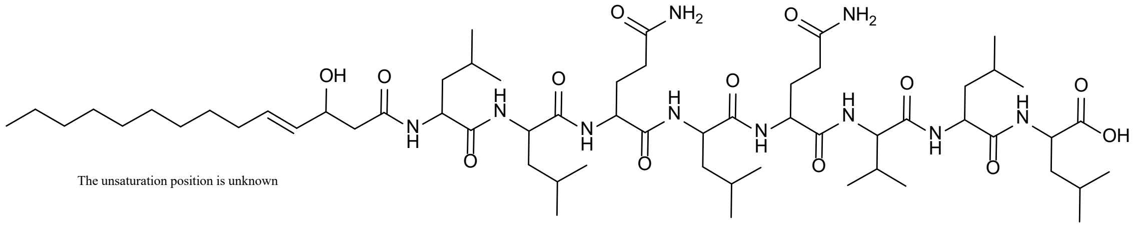

3-OH C14:1 Cichofactin [Val6]

Molecular formula: $C_{59}H_{106}N_{10}O_{13}$

Exact mass: 1 162.79 Da

Supplementary Figure S6B

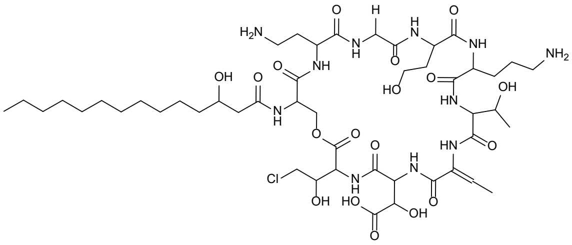

Syringotoxin B = 3-OH C14:0
Syringotoxin
Molecular formula: $C_{48}H_{82}ClN_{11}O_{18}$
Exact mass: 1 135.55 Da

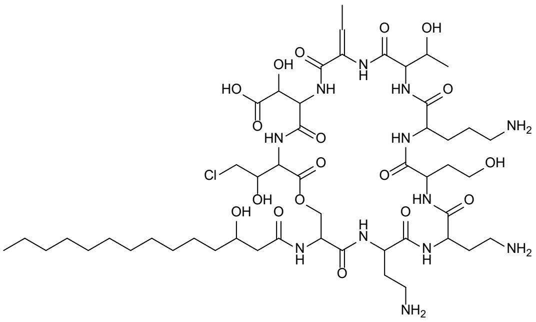

Syringostatin A = 3-OH C14:0
Syringostatin
Molecular formula: $C_{50}H_{87}ClN_{12}O_{18}$
Exact mass: 1 178.59 Da

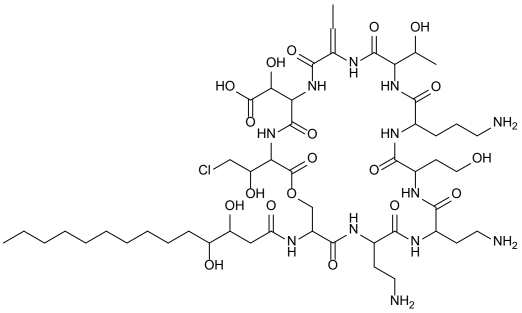

Syringostatin B = 3,4-OH C14:0
Syringostatin
Molecular formula: $C_{50}H_{87}ClN_{12}O_{19}$
Exact mass: 1 194.59 Da

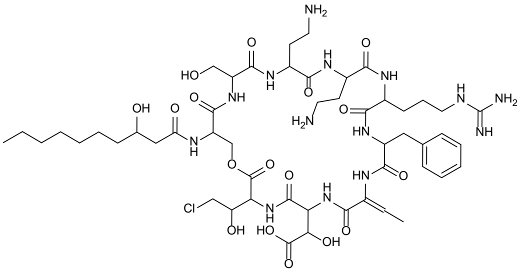

Syringomycin A1 = 3-OH C10:0
Syringomycin
Molecular formula: $C_{51}H_{81}ClN_{14}O_{17}$
Exact mass: 1 196.56 Da

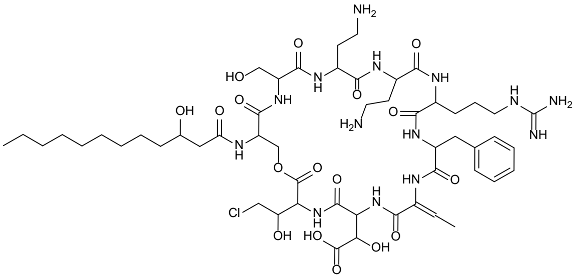

Syringomycin E = 3-OH C12:0
Syringomycin
Molecular formula: $C_{53}H_{85}ClN_{14}O_{17}$
Exact mass: 1 224.59 Da

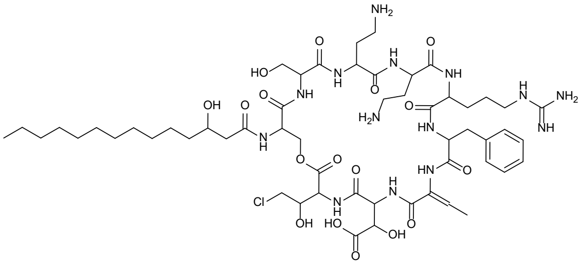

Syringomycin G = 3-OH C14:0
Syringomycin
Molecular formula: $C_{55}H_{89}ClN_{14}O_{17}$
Exact mass: 1 252.62 Da

Supplementary Figure S6B continued.

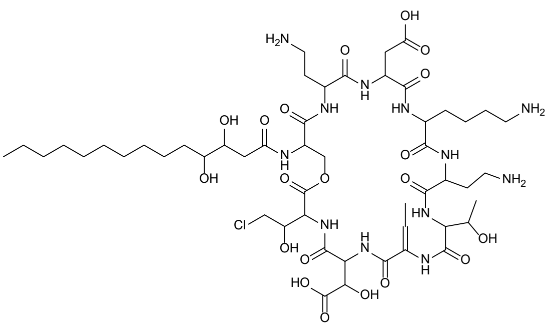

Pseudomycin A = 3,4-OH C14:0 Pseudomycin
Molecular formula: $C_{51}H_{87}ClN_{12}O_{20}$
Exact mass: 1 222.58 Da

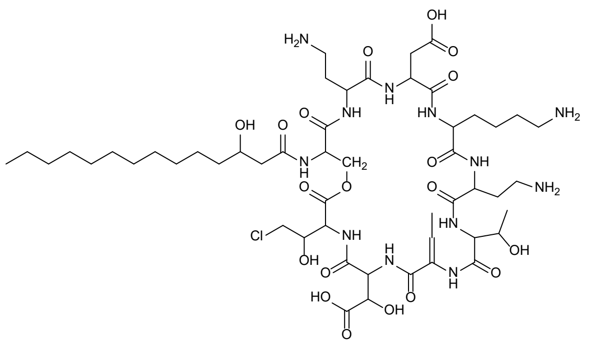

Pseudomycin B = 3-OH C14:0 Pseudomycin
Molecular formula: $C_{51}H_{87}ClN_{12}O_{19}$
Exact mass: 1 206.59 Da

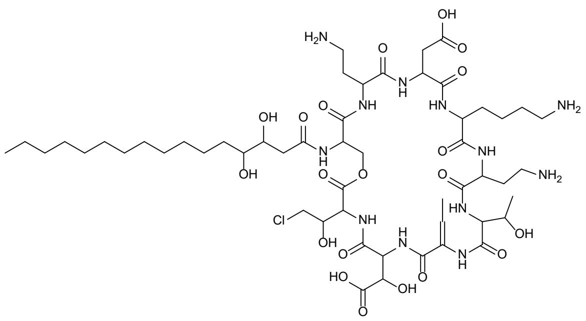

Pseudomycin C = 3,4-OH C16:0 Pseudomycin
Molecular formula: $C_{53}H_{91}ClN_{12}O_{20}$
Exact mass: 1 250.62 Da

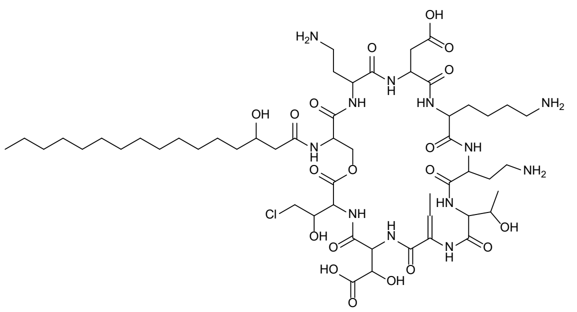

Pseudomycin C' = 3-OH C16:0 Pseudomycin
Molecular formula: $C_{53}H_{91}ClN_{12}O_{19}$
Exact mass: 1 234.62 Da

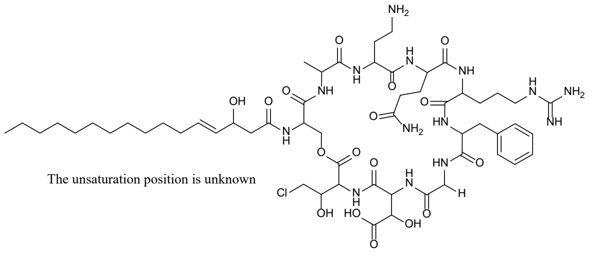

3-OH C16:1 Syringomycin-2
Molecular formula: $C_{56}H_{89}ClN_{14}O_{17}$
Exact mass: 1 264.62 Da

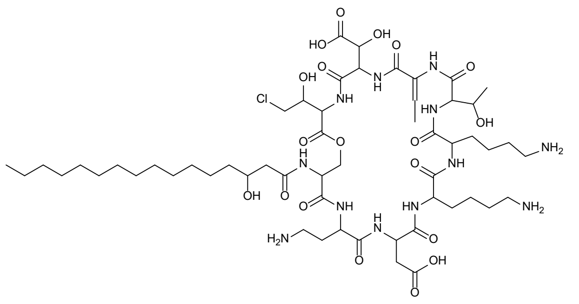

3-OH C16:0 Pseudomycin-2
Molecular formula: $C_{55}H_{95}ClN_{12}O_{19}$
Exact mass: 1 262.65 Da

Supplementary Figure S6C

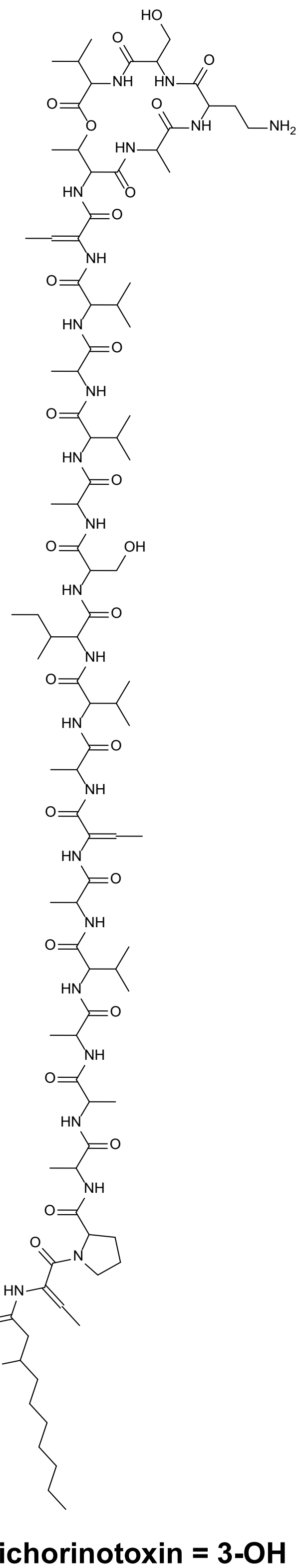

Cichorinotoxin = 3-OH C10:0 Cichorinotoxin
Molecular formula:
$C_{96}H_{161}N_{23}O_{27}$
Exact mass: 2 068.19 Da

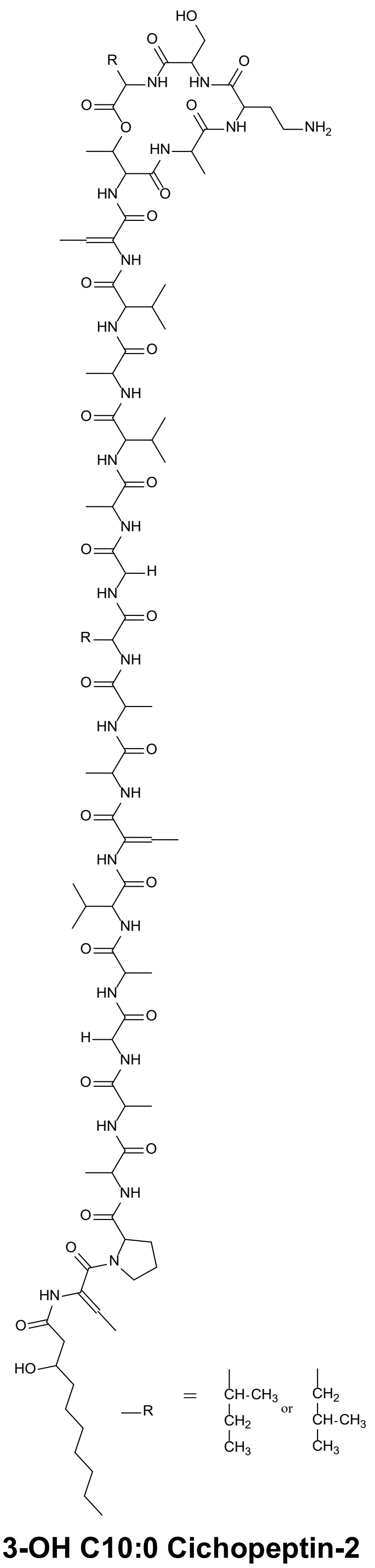

3-OH C10:0 Cichopeptin-2
Molecular formula:
$C_{93}H_{155}N_{23}O_{26}$
Exact mass: 2 010.15 Da

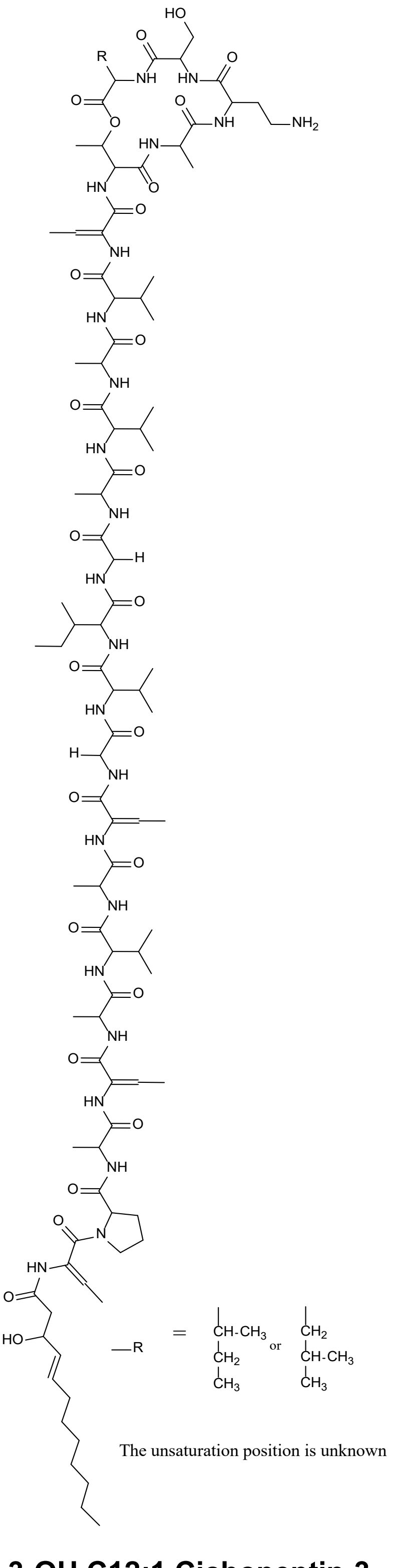

3-OH C12:1 Cichopeptin-3
Molecular formula:
$C_{98}H_{161}N_{23}O_{26}$
Exact mass: 2 076.20 Da

Supplementary Figure S6C continued.

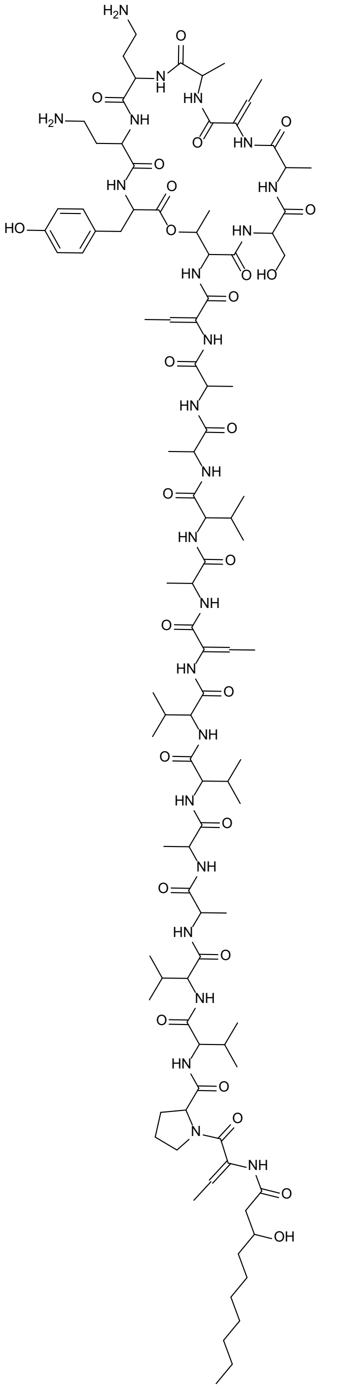

Syringopeptin 22A = 3-OH C10:0
Syringopeptin 22
Molecular formula: $C_{101}H_{162}N_{24}O_{27}$
Exact mass: 2 143.20 Da

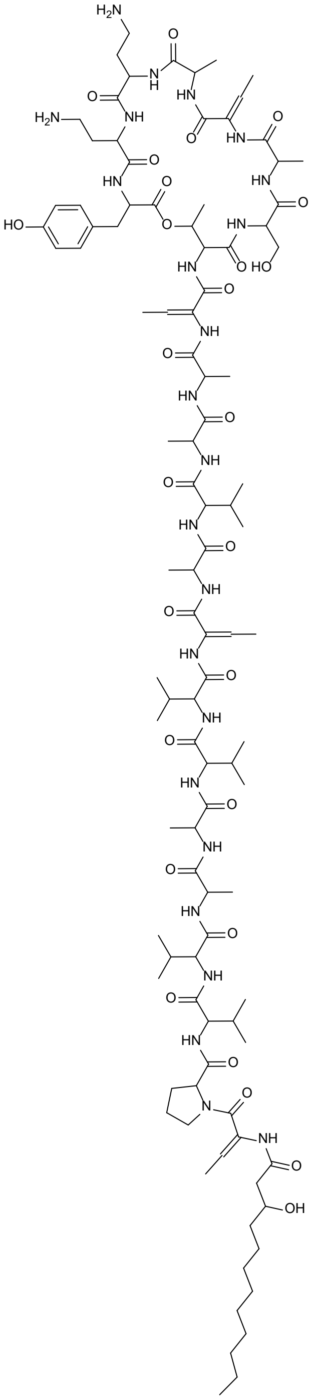

Syringopeptin 22B = 3-OH C12:0
Syringopeptin 22
Molecular formula: $C_{103}H_{166}N_{24}O_{27}$
Exact mass: 2 171.24 Da

Supplementary Figure S6C continued.

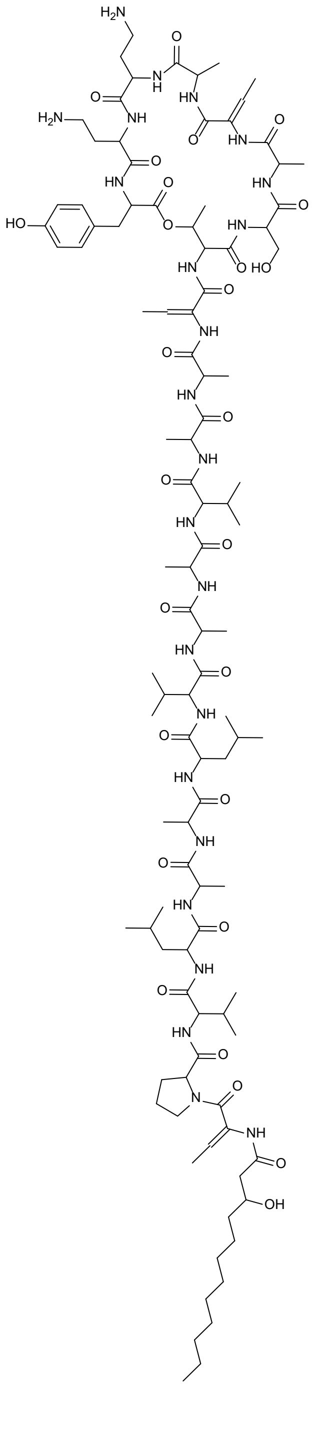

Syringopeptin 508A = 3-OH C12:0
Syringopeptin 508
Molecular formula: $C_{104}H_{170}N_{24}O_{27}$
Exact mass: 2 187.27 Da

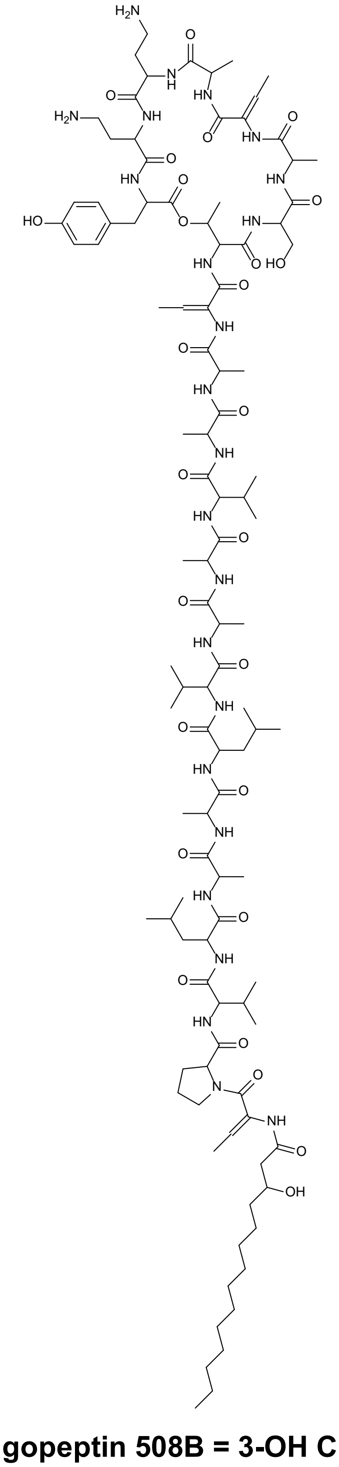

Syringopeptin 508B = 3-OH C14:0
Syringopeptin 508
Molecular formula: $C_{106}H_{174}N_{24}O_{27}$
Exact mass: 2 215.30 Da

Supplementary Figure S6C continued.

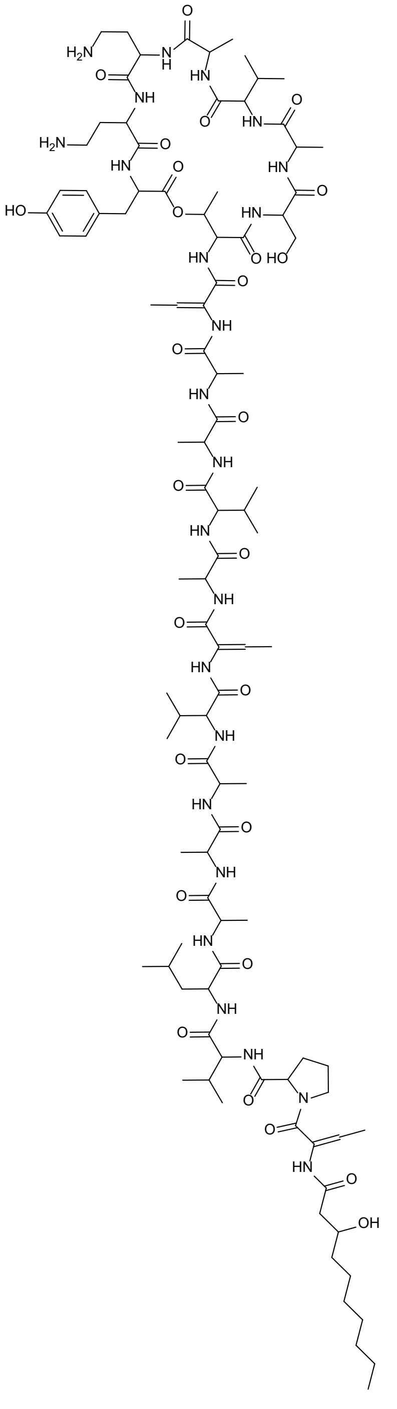

3-OH C10:0 Syringopeptin 22-2
Molecular formula: $C_{101}H_{164}N_{24}O_{27}$
Exact mass: 2 145.22 Da

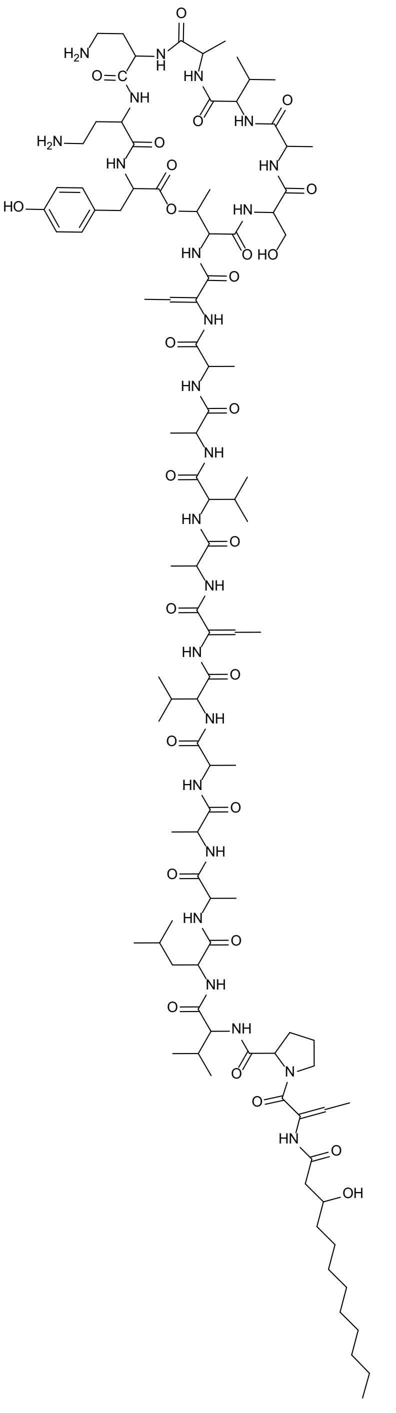

3-OH C12:0 Syringopeptin 22-2
Molecular formula: $C_{103}H_{168}N_{24}O_{27}$
Exact mass: 2 173.25 Da

Supplementary Figure S6C continued.

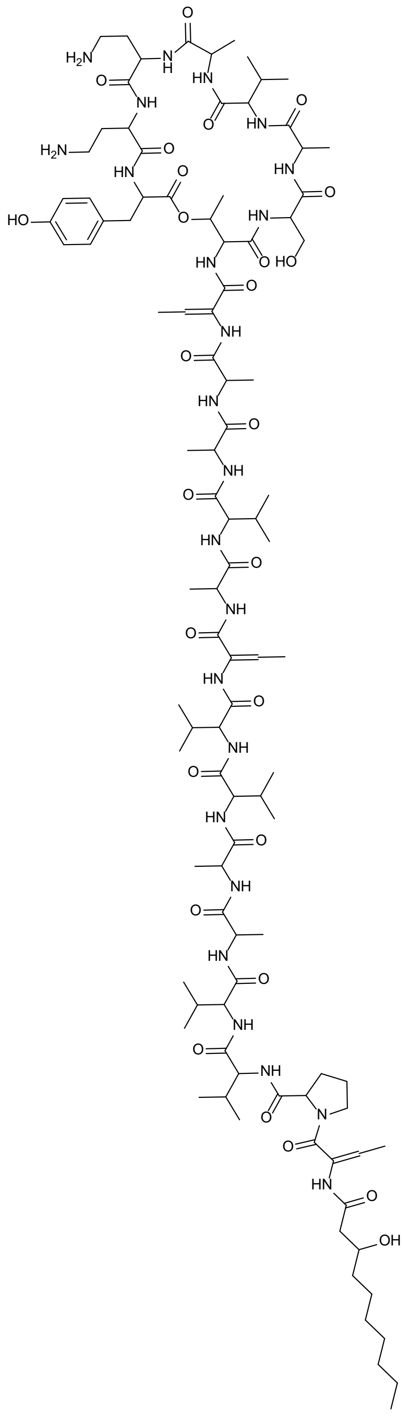

3-OH C10:0 Syringopeptin 22-3
Molecular formula: $C_{102}H_{166}N_{24}O_{27}$
Exact mass: 2 159.24 Da

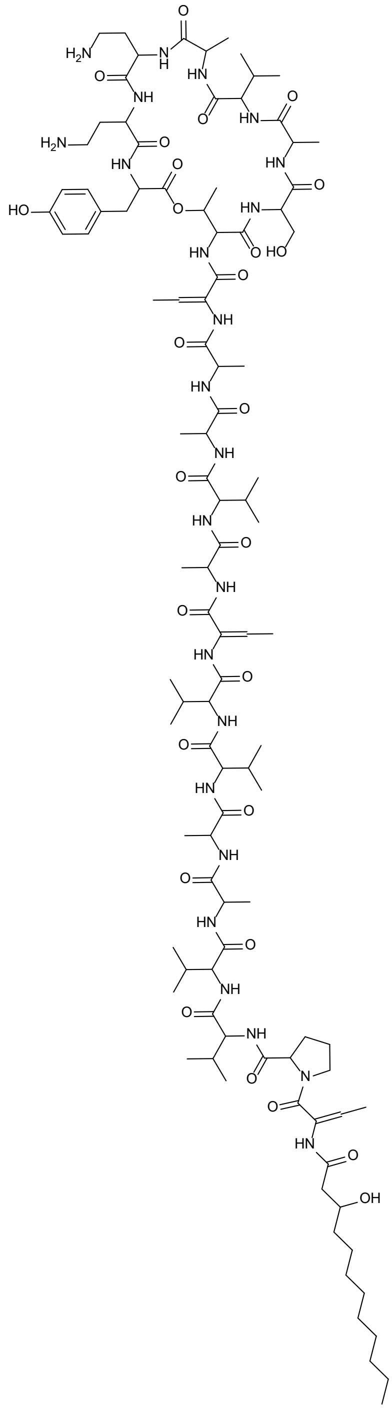

3-OH C12:0 Syringopeptin 22-3
Molecular formula: $C_{104}H_{170}N_{24}O_{27}$
Exact mass: 2 187.27 Da

Supplementary Figure S6C continued.

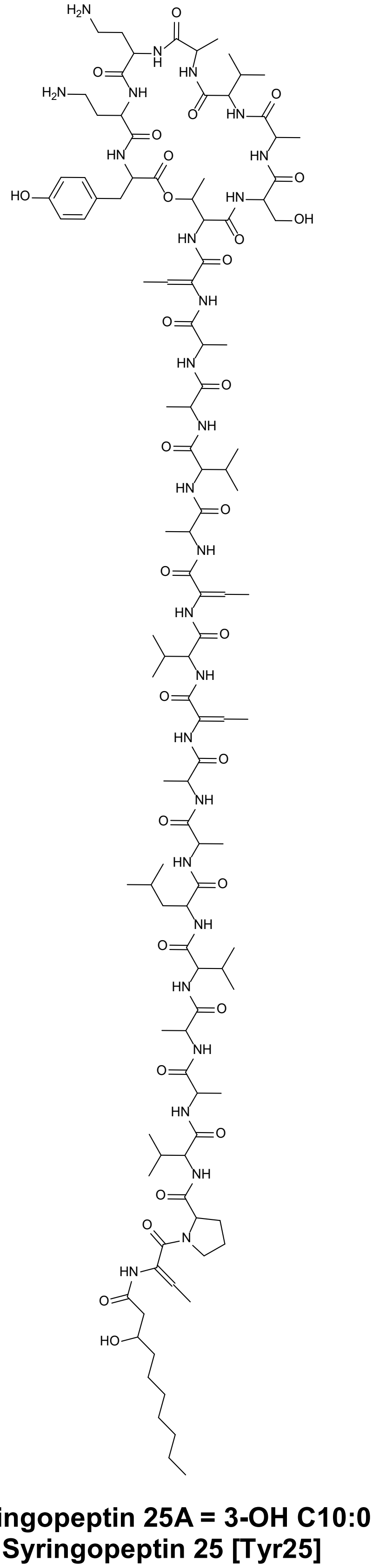

Syringopeptin 25A = 3-OH C10:0
Syringopeptin 25 [Tyr25]
Molecular formula: $C_{113}H_{183}N_{27}O_{30}$
Exact mass: 2 398.36 Da

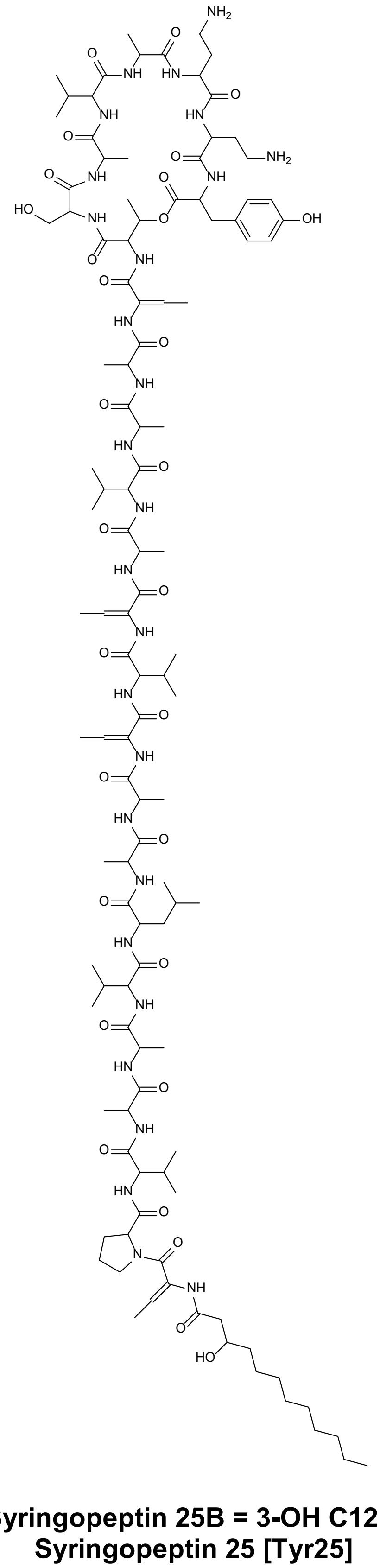

Syringopeptin 25B = 3-OH C12:0
Syringopeptin 25 [Tyr25]
Molecular formula: $C_{115}H_{187}N_{27}O_{30}$
Exact mass: 2 426.39 Da

Supplementary Figure S6C continued.

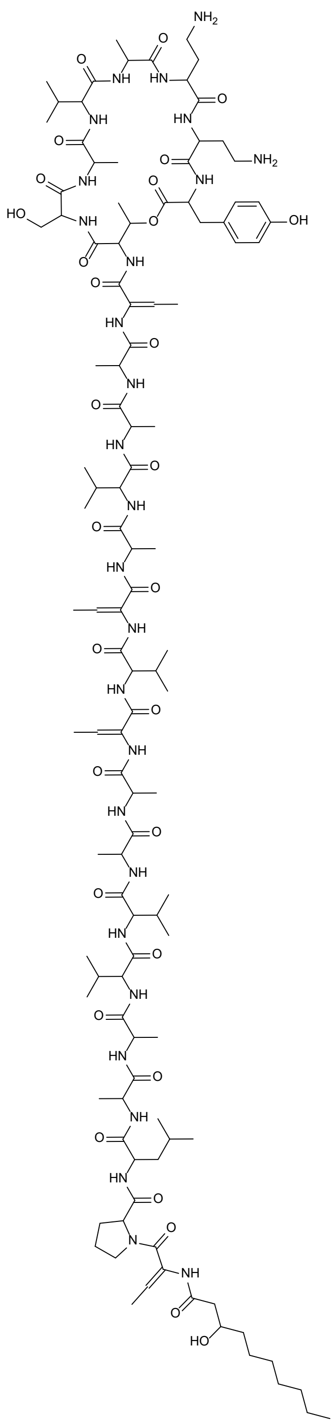

3-OH C10:0 Syringopeptin 25-2
Molecular formula: $C_{113}H_{183}N_{27}O_{30}$
Exact mass: 2 398.36 Da

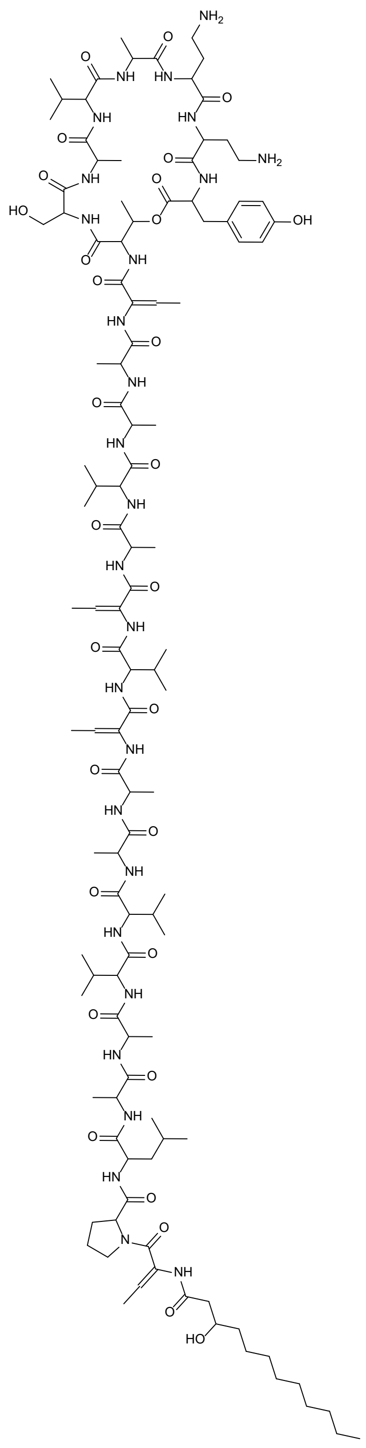

3-OH C12:0 Syringopeptin 25-2
Molecular formula: $C_{115}H_{187}N_{27}O_{30}$
Exact mass: 2 426.39 Da

Supplementary Figure S6C continued.

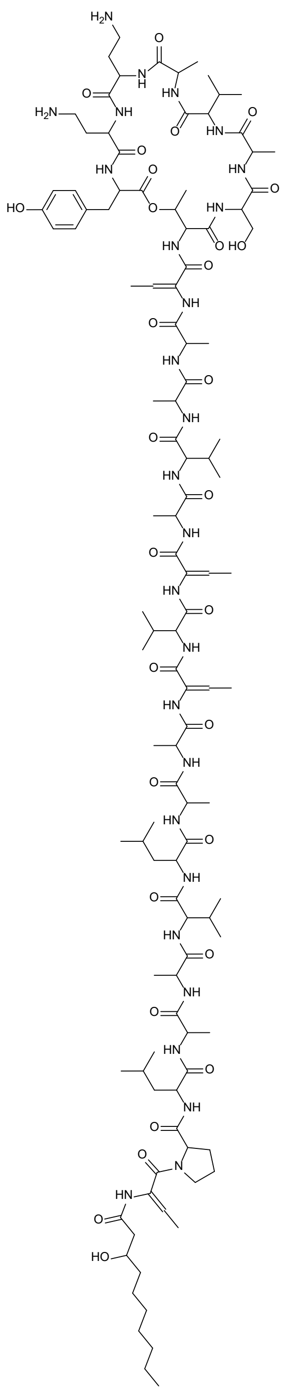

3-OH C10:0 Syringopeptin 25-3
Molecular formula: $C_{114}H_{185}N_{27}O_{30}$
Exact mass: 2 412.38 Da

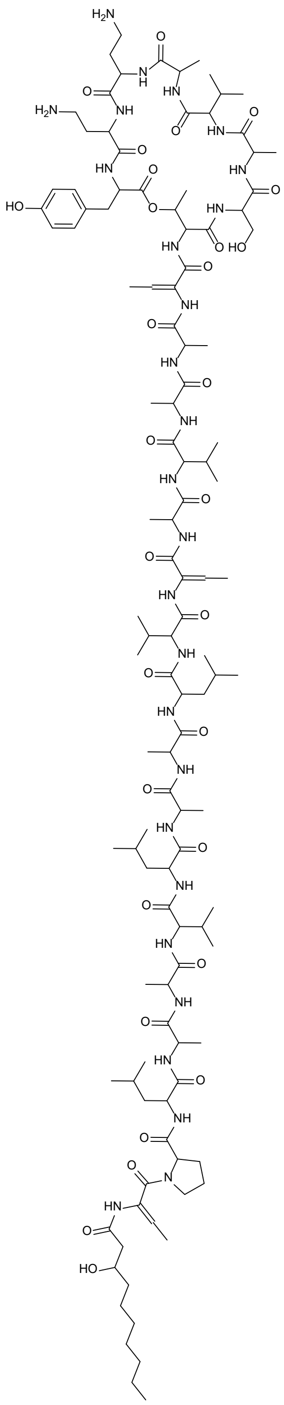

3-OH C10:0 Syringopeptin 25-5
Molecular formula: $C_{116}H_{191}N_{27}O_{30}$
Exact mass: 2 442.43 Da

Supplementary Figure S6C continued.

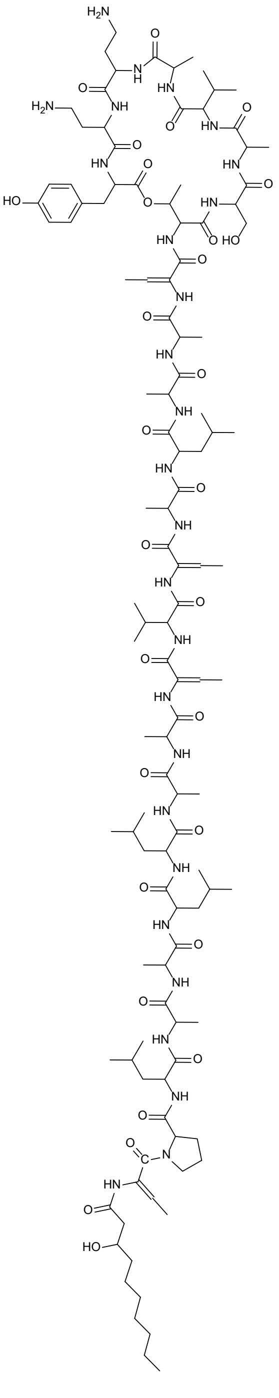

3-OH C10:0 Syringopeptin 25-4
Molecular formula: $C_{116}H_{189}N_{27}O_{30}$
Exact mass: 2 440.41 Da

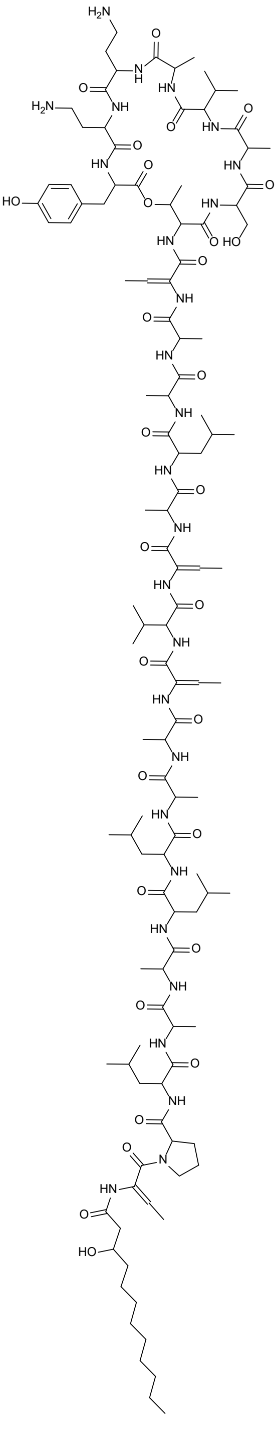

3-OH C12:0 Syringopeptin 25-4
Molecular formula: $C_{118}H_{193}N_{27}O_{30}$
Exact mass: 2 468.44 Da

Supplementary Figure S6Ccontinued.

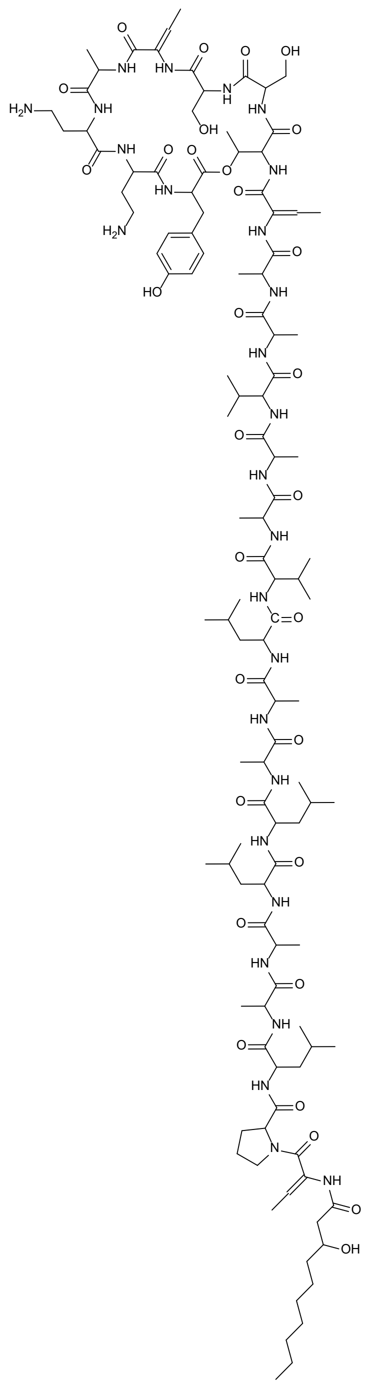

3-OH C10:0 Syringopeptin 25-6
Molecular formula: $C_{115}H_{189}N_{27}O_{31}$
Exact mass: 2 444.40 Da

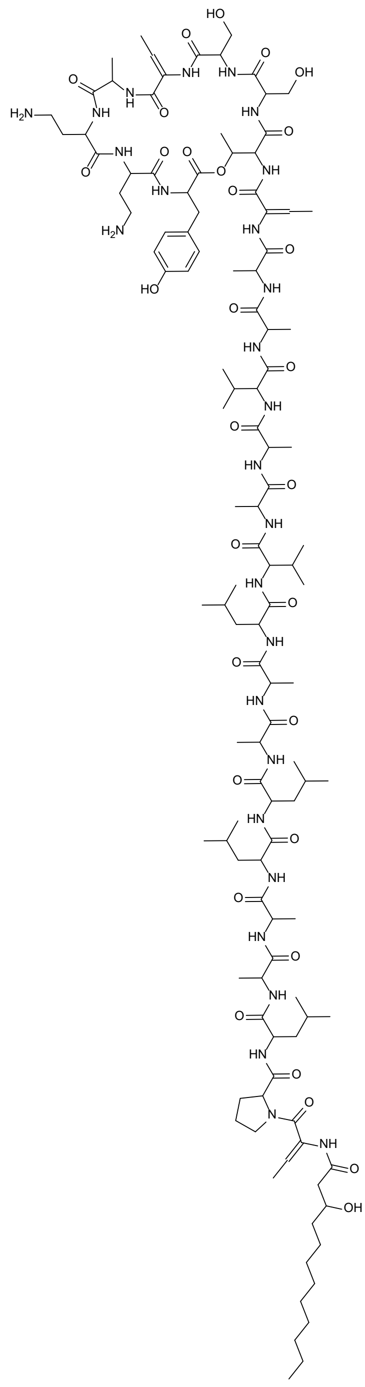

3-OH C12:0 Syringopeptin 25-6
Molecular formula: $C_{117}H_{193}N_{27}O_{31}$
Exact mass: 2 472.44 Da
